# Identification of differentially methylated *BRCA1* and *CRISP2* DNA regions as blood surrogate markers for cardiovascular disease

**DOI:** 10.1038/s41598-017-03434-0

**Published:** 2017-07-11

**Authors:** Geoffrey Istas, Ken Declerck, Maria Pudenz, Katarzyna Szarc vel Szic, Veronica Lendinez-Tortajada, Montserrat Leon-Latre, Karen Heyninck, Guy Haegeman, Jose A. Casasnovas, Maria Tellez-Plaza, Clarissa Gerhauser, Christian Heiss, Ana Rodriguez-Mateos, Wim Vanden Berghe

**Affiliations:** 1 Division of Cardiology, Pulmonology, and Vascular Medicine, Medical Faculty, Düsseldorf University, Düsseldorf, Germany; 2 Laboratory of Protein chemistry, Proteomics and Epigenetic Signaling (PPES), Department of Biomedical Sciences, Faculty of Pharmaceutical, Biomedical and Veterinary Sciences, Antwerp University, Antwerp (Wilrijk), Belgium; 3 Workgroup Cancer Chemoprevention and Epigenomics, Division of Epigenomics and Cancer Risk Factors, German Cancer Research Center (DKFZ), Heidelberg, Germany; 4 Division of Hematology, Oncology and Stem Cell Transplantation, Center for Translational Cell Research, The University Medical Center Freiburg, Freiburg, Germany; 5 Genomic and Genetic Diagnosis Unit, Institute for Biomedical Research Hospital Clinic de Valencia, Valencia, Spain; 6 Servicio Aragones de Salud, Zaragoza, Spain; 7 IIS de Aragon, Zaragoza, Spain; 8 Laboratory of Eukaryotic Gene Expression and Signal Transduction LEGEST, Department of Biochemistry and Microbiology, Ghent University, Gent, Belgium; 9 Instituto Aragonés de Ciencias de Salud, Zaragoza, Spain; 10 Universidad de Zaragoza, Zaragoza, Spain; 11 Workgroup Cardiometabolic and Renal Risk, Institute for Biomedical Research Hospital Clinic de Valencia, Valencia, Spain; 12 Division of Diabetes and Nutritional Sciences, Faculty of Life Sciences and Medicine, King’s College, London, UK

## Abstract

Genome-wide Illumina InfiniumMethylation 450 K DNA methylation analysis was performed on blood samples from clinical atherosclerosis patients (n = 8) and healthy donors (n = 8) in the LVAD study (NCT02174133, NCT01799005). Multiple differentially methylated regions (DMR) could be identified in atherosclerosis patients, related to epigenetic control of cell adhesion, chemotaxis, cytoskeletal reorganisations, cell proliferation, cell death, estrogen receptor pathways and phagocytic immune responses. Furthermore, a subset of 34 DMRs related to impaired oxidative stress, DNA repair, and inflammatory pathways could be replicated in an independent cohort study of donor-matched healthy and atherosclerotic human aorta tissue (n = 15) and human carotid plaque samples (n = 19). Upon integrated network analysis, *BRCA1* and *CRISP2* DMRs were identified as most central disease-associated DNA methylation biomarkers. Differentially methylated *BRCA1* and *CRISP2* regions were verified by MassARRAY Epityper and pyrosequencing assays and could be further replicated in blood, aorta tissue and carotid plaque material of atherosclerosis patients. Moreover, methylation changes at *BRCA1* and *CRISP2* specific CpG sites were consistently associated with subclinical atherosclerosis measures (coronary calcium score and carotid intima media thickness) in an independent sample cohort of middle-aged men with subclinical cardiovascular disease in the Aragon Workers’ Health Study (n = 24). Altogether, *BRCA1* and *CRISP2* DMRs hold promise as novel blood surrogate markers for early risk stratification and CVD prevention.

## Introduction

According to the World Health Organization (WHO), cardiovascular diseases (CVD) account for the highest mortality numbers with approximately 30% of all deaths worldwide (http://www.who.int/gho/ncd/en/). Atherosclerosis is the major principle underlying CVDs. At predisposed areas of the vascular tree, including the branching points of coronary and carotid arteries, localized accumulation of fatty deposits and inflammation reactions contribute to plaque development and progression eventually leading to impaired blood flow resulting in CVDs i.e. coronary artery disease and cerebrovascular disease^1^.

The development of an atherosclerotic lesion is a slow and silent process making early stage diagnosis difficult^2^. Early detection of individuals in the process of developing atherosclerosis might be essential for cardiovascular prevention. Approximately 60% of individuals categorized as at low risk for cardiovascular disease based on traditional risk factors prediction equations had subclinical atherosclerosis^3, 4^. Thus, other factors not traditionally included in risk scales are likely to be involved in atherogenesis.

Preclinical evidence supports that aberrant monocyte-macrophage differentiation contributes to vascular wall inflammation in patients at high risk for atherosclerosis^5, 6^. CpG DNA methylation is involved in the epigenetic differentiation and regulation of leukocyte specific gene expression profiles, including the expression of soluble mediators and surface molecules that direct margination, adhesion, and migration of blood leukocytes in vascular tissues^7^. While very little is known about the human leukocyte DNA methylome and its potential causal role in cardiovascular disease, blood DNA methylation markers may contribute to the diagnosis of atherosclerosis patients. Recent studies have illustrated the feasibility of DNA methylation profiling using peripheral blood to identify CVD specific surrogate biomarkers^8–15^. Candidate-gene approaches identified significant associations between leukocyte DNA methylation and atherosclerosis, whereas the results for the association between global DNA methylation and atherosclerosis were not always consistent^9–12^. Previous studies however, evaluated differentially methylated sites using samples from individuals with clinical cardiovascular disease, but did not examine the potential role of DNA methylation regions as a marker of subclinical disease.

Therefore, we characterized genome-wide DNA methylation profiles of blood samples of atherosclerosis patients versus healthy individuals from the LVAD study (Impact of Left Ventricular Assist Devices Implantation on Micro- and Macrovascular Function, (NCT02174133) and identified promising atherosclerosis-related epigenetic biomarkers. For the selected regions, we validated these whole blood DNA methylation profiles using publicly available Illumina InfiniumMethylation 450 K data from carotid normal and atherosclerotic plaque samples. Additionally, we compared CVD associated DNA methylation changes with aging and/or immune cell epigenotypes. Finally, we explored the role of promising regions as potential predictors of subclinical atherosclerosis in a subsample of 24 individuals that participated in the Aragon Workers Health Study (AWHS). The AWHS is a prospective cohort that aims to characterize the factors associated with metabolic abnormalities and imaging-based subclinical atherosclerosis measures in a middle-aged population free of clinical cardiovascular disease^3, 16^.

## Results

### Peripheral blood of atherosclerosis patients reveals no statistically significant changes in global DNA methylation in comparison to healthy individuals

Significant differences in clinical parameters were observed between the two study groups. C-reactive protein, haemoglobin concentration and triglycerides were significantly lower in the healthy individuals as compared to atherosclerosis patients (Table 1). The atherosclerosis patients were generally older than the healthy individuals (Table 1).

**Table 1:** Volunteer characteristics.

Clinical parameters	Healthy	Atherosclerosis	P value
	Mean ± SD	Mean ± SD	
Coronary artery disease	No	Yes	
Arterial hypertension	No	Yes	
Arrhythmia	No	Yes	
Age	49.7 ± 6.6	78.0 ± 8.4	< 0.0001
BMI (kg/m^2^)	26.2 ± 2.3	27.0 ± 3.2	0.61
LDL cholesterol (mg/dl)	147.4 ± 37.2	121.3 ± 14.5	0.13
HDL cholesterol (mg/dl)	52.3 ± 11.2	46.8 ± 10.1	0.41
Total cholesterol (mg/dl)	212.7 ± 38.0	179.0 ± 32.5	0.09
Fasting plasma glucose (mg/dl)	90.0 ± 8.0	86.1 ± 14.3	0.54
HbA1c (%)	5.7 ± 0.3	5.8 ± 0.9	0.78
CRP (mg/dl)	0.6 ± 0.5	0.7 ± 0.5	0.04
Leukocytes (1000/ul)	6.6 ± 2.3	6.4 ± 1.4	0.84
Hb (mg/dl)	12.7 ± 1.7	14.9 ± 0.6	0.007
Creatinine (mg/dl)	1.0 ± 0.2	1.3 ± 0.5	0.31
Triglycerides (mg/dl)	95.9 ± 44.2	159.5 ± 48.0	0.03

Statistical analysis performed by means of the student T test.

DNA methylation profiles covering >450,000 CpG dinucleotides of peripheral blood of eight atherosclerosis and eight healthy individuals were generated by Illumina 450k BeadChip arrays. CpG probes were sub-grouped in relation to gene regions and to CpG islands to obtain the most comprehensive view of the DNA methylation distribution in both groups. Global DNA methylation was assessed in each individual by calculating the median beta-value of all CpG probes. The mean median values were calculated per sample group (healthy and atherosclerosis). Overall, no statistical significant difference (P-value = 0.9159) was observed in mean global DNA methylation between atherosclerosis patients (0.6613, SEM: 0.0067) versus healthy controls (0.6624, SEM: 0.0071). In addition, no global DNA methylation shifts were found between the groups when mapping cg probes to different genomic locations (e.g. CpG islands, shores, shelves) (Supplementary Fig. 1).

### Peripheral blood of atherosclerosis patients reveals DNA methylation signature of impaired regulation of cell adhesion, chemotaxis and estrogen hormone responses

**Figure 1:**
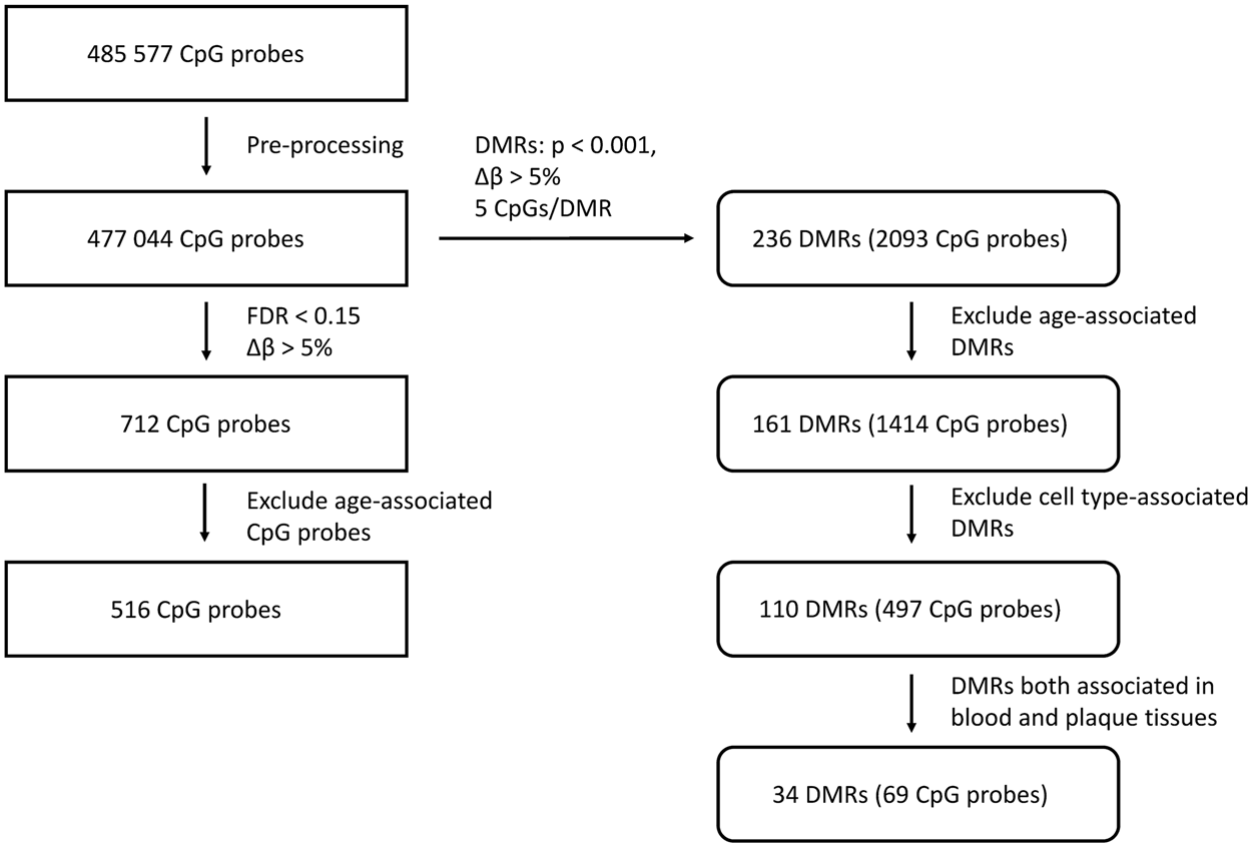
Flowchart of DMP and DMR selection.

**Figure 2:**
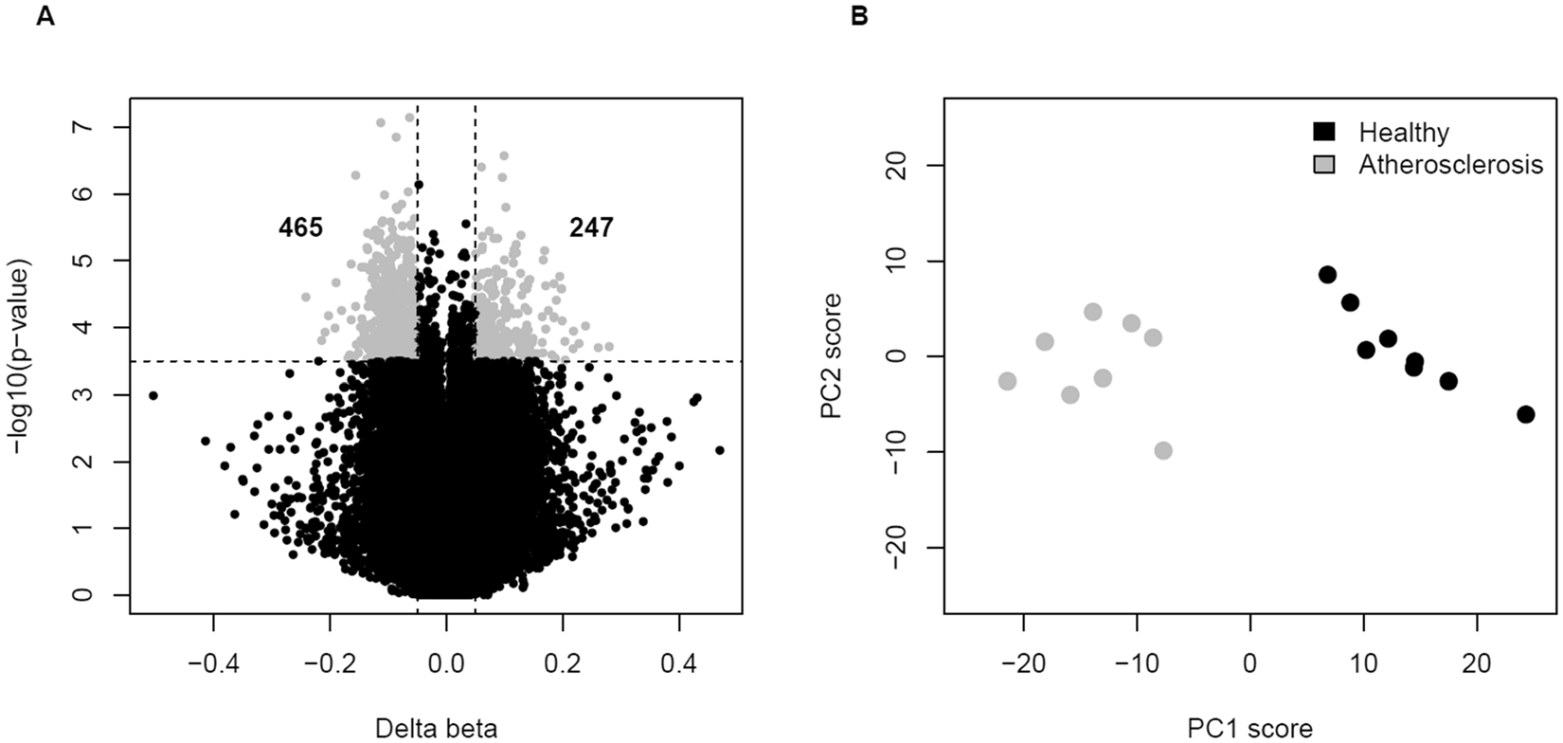
Differentially methylated CpG probes between healthy and atherosclerotic individuals. Sig-DMPs were selected based on FDR < 0.15 and Δ beta >5%. (**A**) Volcano plot showing 465 hypo- and 247 hypermethylated probes meeting the selection criteria. (**B**) PCA plot demonstrating the separation of atherosclerosis patients for CVD (grey) and healthy individuals (black) based on the DNA methylation values of the 712 DMPs.

**Figure 3:**
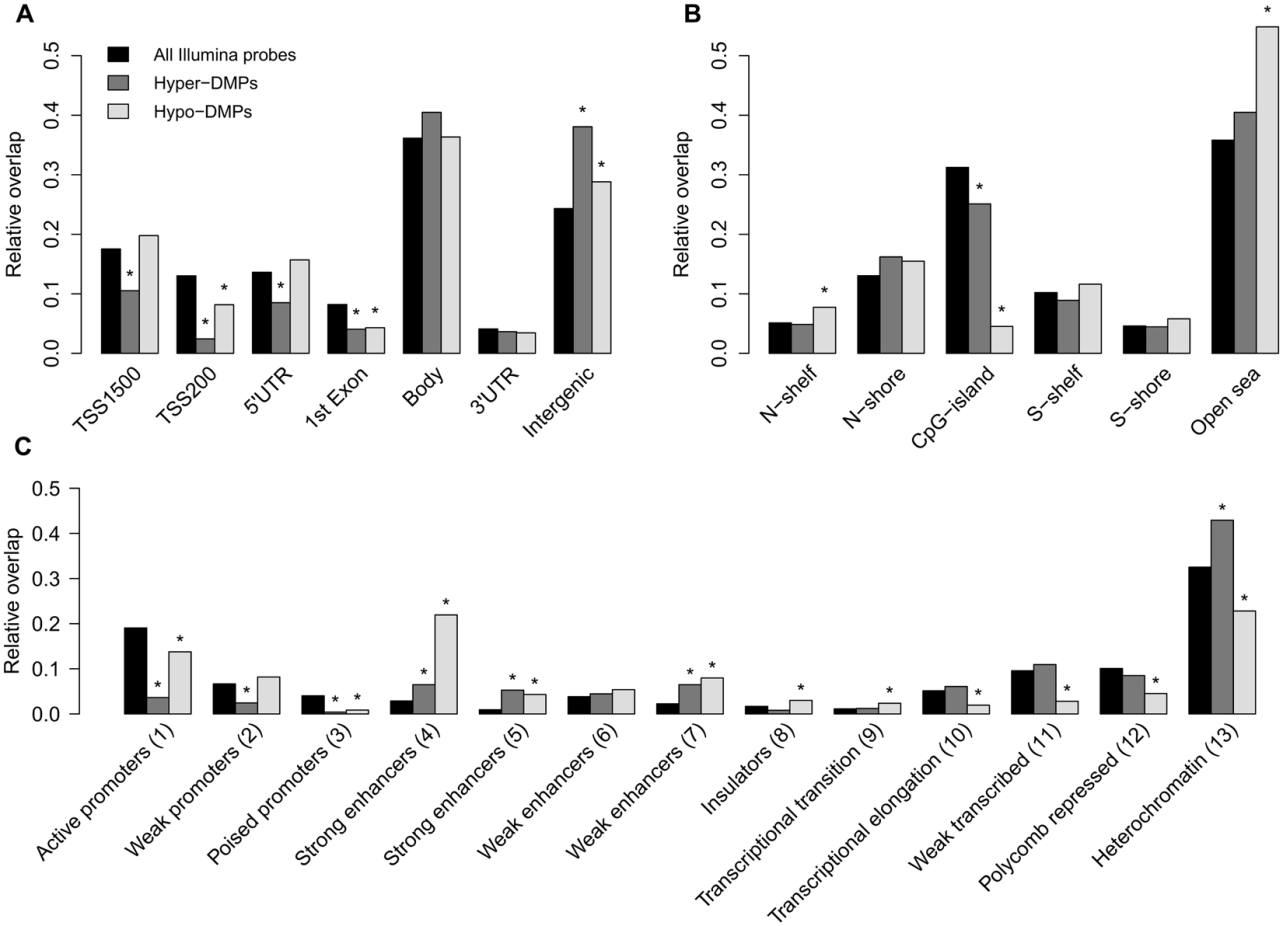
Genomic distribution of sig-DMPs based on (**A**) gene regions, (**B**) CpG-island regions and (**C**) chromatin segmentation states (based on GM12878 cell type data). Significant enrichment or depletion of sig-DMPs (P-value < 0.05), determined by the Fisher’s exact test, are marked by an asterisk.

Criteria for the identification of sig-DMPs are summarized in Fig. 1. Normalization, quality control and probe filtering for SNPs resulted in an output of 477,044 CpG probes. Differentially methylated CpG probes were filtered for a Benjamini-Hochberg adjusted p-value smaller than 0.15 and a difference in β-values between atherosclerosis patients and healthy controls of at least 0.05 (i.e. 5% difference in DNA methylation). 712 CpG probes met the selection criteria comprising 465 hypomethylated CpG sites (hypo-DMPs) and 247 hypermethylated CpG sites (hyper-DMPs) (Fig. 2A, Supplementary Table 2). We observed a maximum of 20% difference in DNA methylation between atherosclerosis patients and healthy controls. Based on the methylation values of these CpG sites, principle component analysis reveals a clear separation of DNA methylation profiles of atherosclerosis and healthy individuals (Fig. 2B). Analysis of functional genomic locations using Epi-explorer^17^ revealed that both hyper- and hypo-DMPs were depleted in promoter regions and CpG islands and enriched in intergenic, CpG-poor and enhancer regions (Fig. 3).

Since median age of atherosclerosis patients (78 ± 8 years old) and healthy controls (47 ± 8 years old) was significantly different (Table 1), we next overlapped our sig-DMP list with the list of age-responsive CpG probes, identified by Steengenga *et al*.^18^, to discriminate between aging- and atherosclerosis-specific DNA methylation changes. Of the 7,477 CpG probes with age-dependent methylation changes, 196 probes overlapped with our sig-DMP list, resulting in 516 age-independent DMPs, including 287 hypo- and 229 hyper-DMPs (Supplementary Table 2).

**Figure 4:**
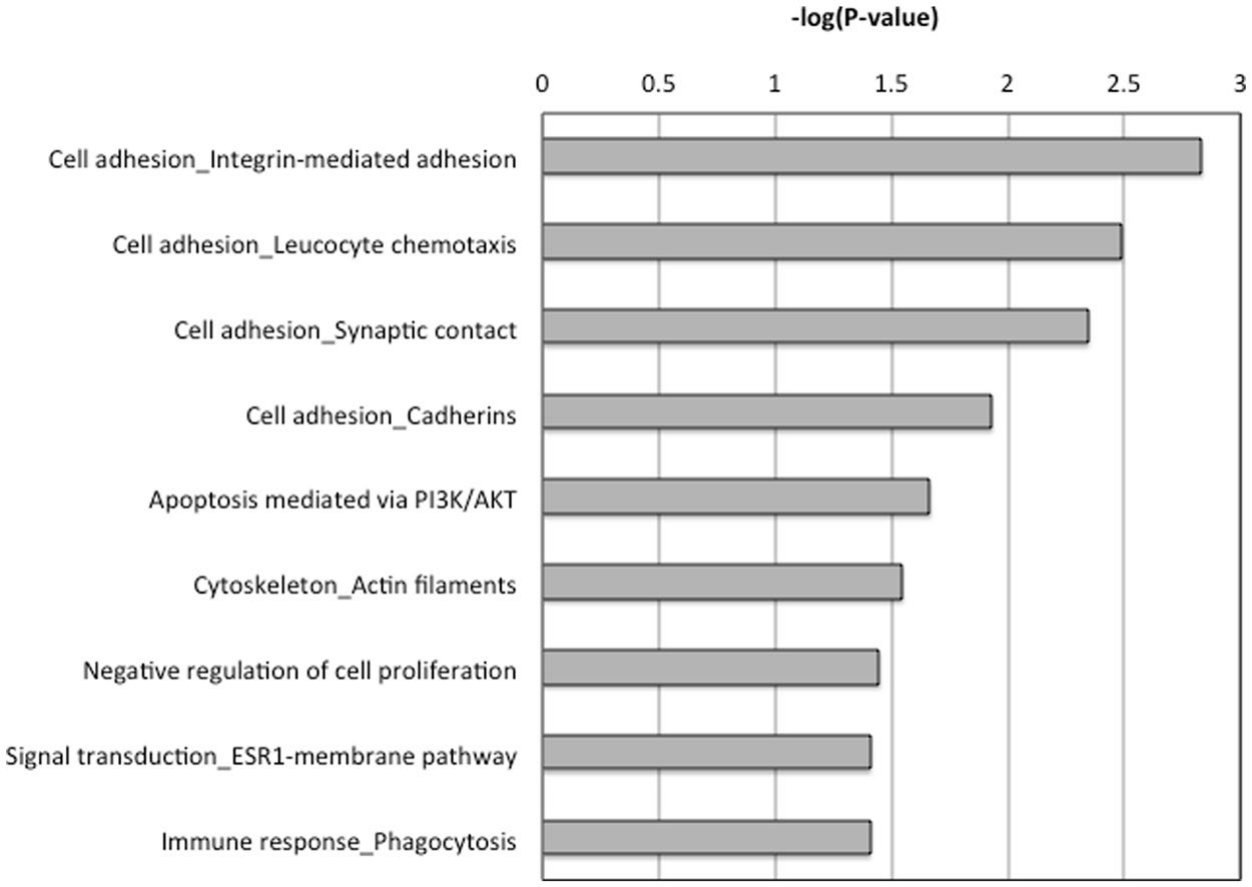
Metacore based pathway enrichment analysis of gene associated DMRs in atherosclerosis reveals a significant (P-value < 0.05; −log(P-value) < 1.3) enrichment of CVD related pathways related to leukocyte chemotaxis and adhesion.

To further reduce biological complexity of DNA methylation changes, we next determined consecutive sig-DMPs using the R-package DMRcate. In total 236 sig-DMRs were identified (P_mean_-value < 0.001) containing at least 5 consecutive CpG probes with a minimal DNA methylation difference of 5% (Δβ-value >0.05) (Fig. 1 and Supplementary Table 3). After overlap with known age-dependent CpG probes^18^, 75 DMRs were removed leaving a total of 161 DMRs (sig-DMRs), containing 1,424 CpG probes, for further analysis. Interestingly, further pathway enrichment analysis of the various gene associated DMRs (Supplementary Table 3), identified significant (P < 0.05) enrichment of cadherin and integrin dependent cell adhesion, chemotaxis, cytoskeletal reorganisations, cell cycle and cell death responses, nongenomic estrogen receptor pathways, immune phagocytosis, all of which are critically involved in atherosclerosis (Fig. 4 and Supplementary Table 4).

### Blood samples of atherosclerosis patients reveal a different immune cell type composition in comparison to healthy individuals

**Figure 5:**
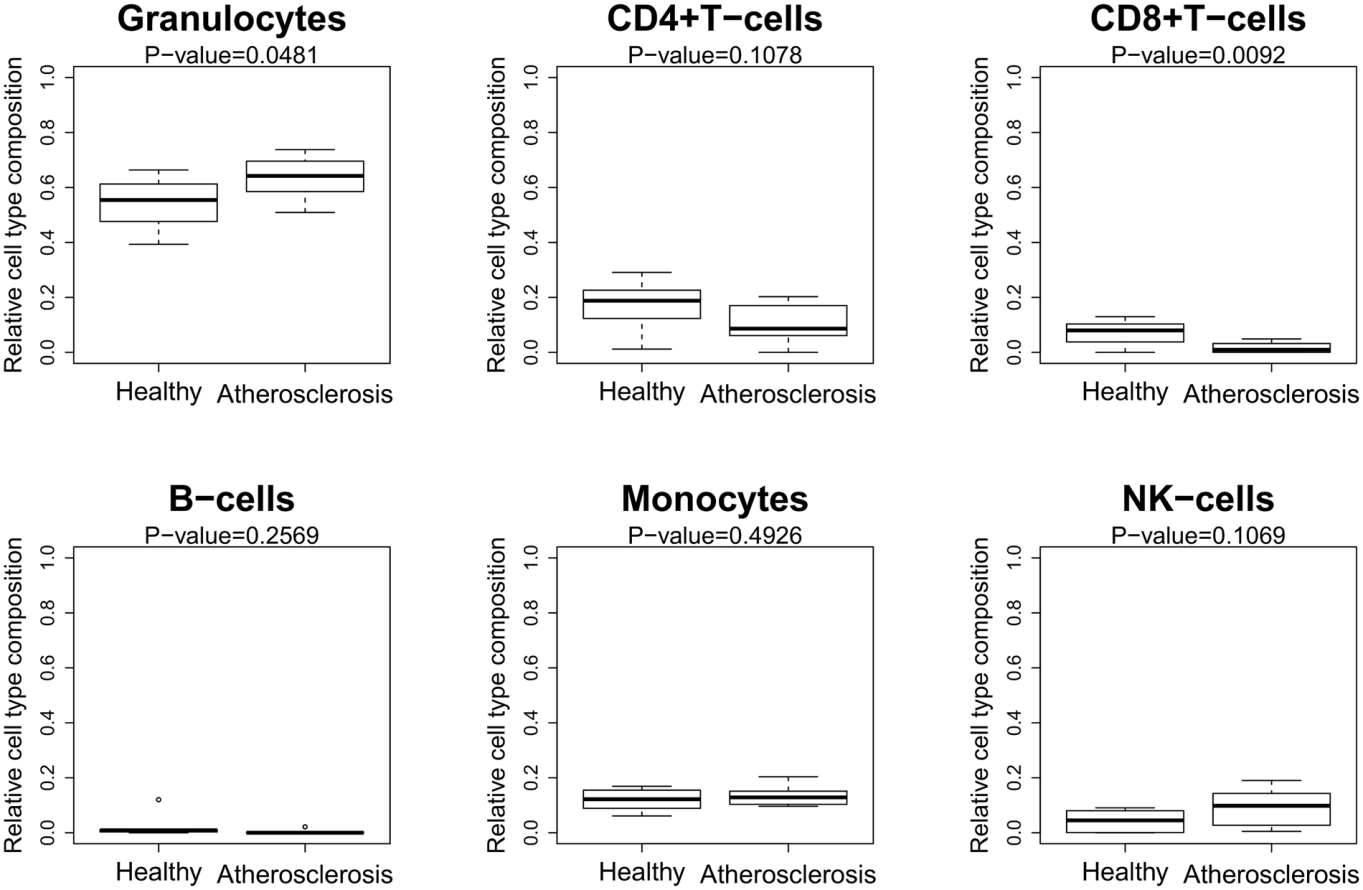
Deconvolution of immune cell type blood composition. The approach described by Houseman *et al*. was applied to determine the relative immune cell type fraction (Y-axis) in healthy and atherosclerosis blood samples. Statistical significant differences in cell type contribution between healthy and atherosclerosis blood samples were calculated by a student t-test.

Since blood is a heterogeneous collection of different cell types, each characterized by unique DNA methylation profile, as recently demonstrated by Jaffe and colleagues^19^, we next wanted to evaluate whether identified DNA methylation changes did not simply reflect variation in blood cell composition between both studied populations. Using the algorithm developed by Houseman and colleagues^20^ for mathematical deconvolution of relative immune cell type composition of blood samples based on Illumina 450k data^21^, we observed that the fraction of granulocytes was slightly but statistical significantly (P < 0.05) increased in patients at high cardiovascular disease risk in comparison to control individuals (Fig. 5 and Supplementary Table 5). In contrast, the CD8^+^ T-cell population was significantly reduced in atherosclerosis patients. Although statistically not significant, a fraction of CD4^+^ T- and NK-cells tended to decrease in atherosclerosis patient. Because of the relative low sample size analysed, we were not able to correct for this in the linear model analysis.

### Pathway enrichment analysis of common gene associated DMRs in blood, aorta and carotid plaque material reveals impaired NRF2 oxidative stress, DNA repair, thioredoxin and inflammatory pathways

**Figure 6:**
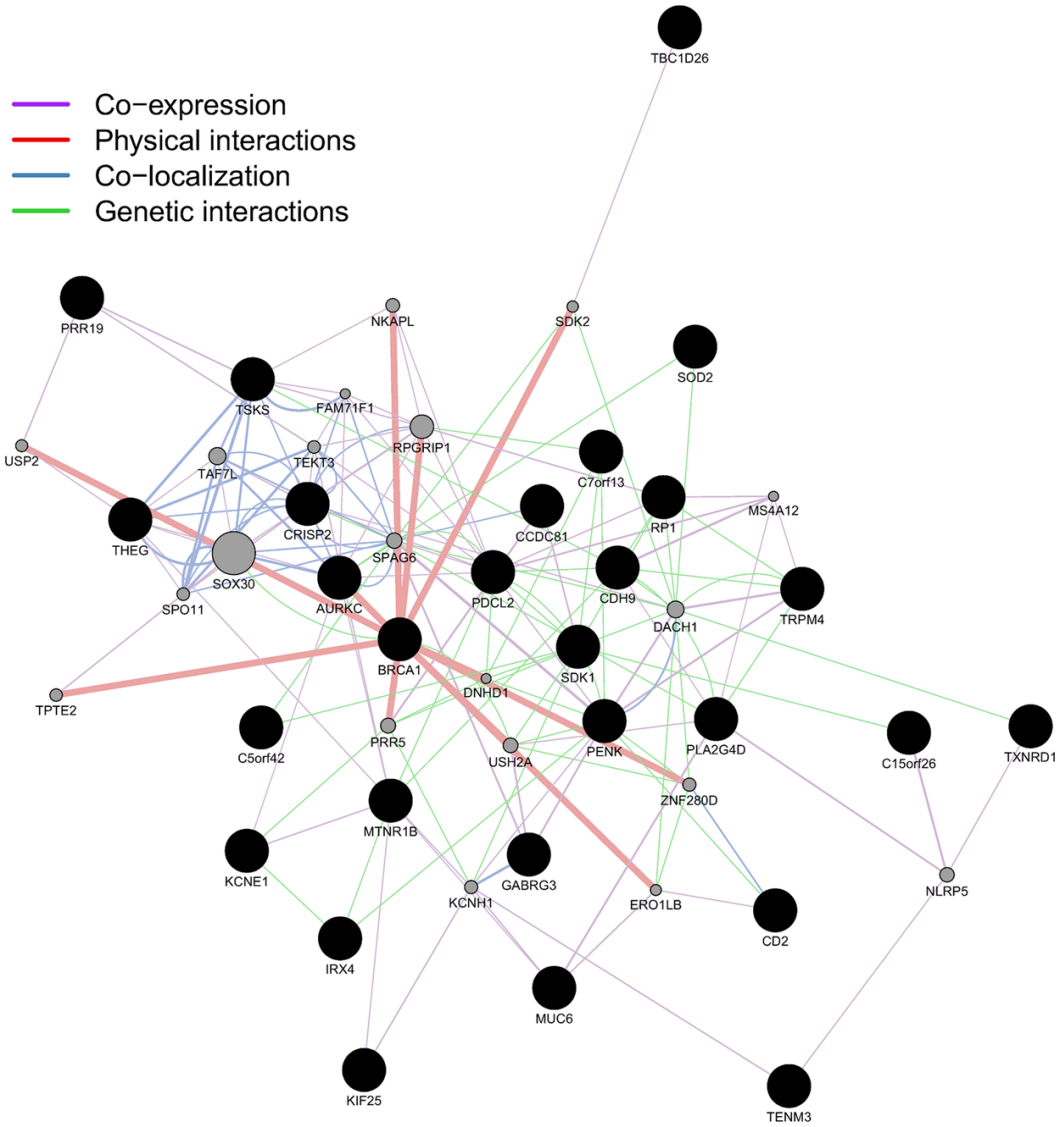
GeneMANIA network of the 34 sig-DMRs not affected by age or cell type composition and consistently differentially methylated in both blood and plaque tissue.

In total, 51 cell type-associated DMRs were excluded using the houseman method leaving 110 sig-DMRs for further analysis. To replicate the potential role for the 110 sig-DMRs as blood-based surrogate biomarkers for plaque biopsy material in atherosclerosis, we compared our findings with publicly available data from Zaina *et al*. (GSE46401)^22^ which contains DNA methylation profiles from donor-matched healthy and atherosclerotic human aorta tissue and from human carotid plaque samples in a larger independent cohort^22^. We performed a two-tailed student t-test for the 497 CpG probes located in the 110 sig-DMRs comparing methylation values between donor-matched healthy and aorta plaque tissue and between healthy aorta and carotid plaque tissue. We found that 69 CpG probes located in 34 unique DMRs were both consistently differentially methylated in our blood-based dataset, in aorta plaque tissue and carotid plaque tissue (Supplementary Table 6). Interestingly, pathway enrichment analysis of the 34-common gene associated DMRs revealed Nuclear factor (erythroid-derived 2)-like 2 (*NRF2*), oxidative stress (*SOD2*), DNA repair (*BRCA1*), thioredoxin (*TXNRD1*) and inflammatory pathways (*MIF*, *PLA2G4D*) (Supplementary Table 7). To find out the relationship between the different sig-DMRs, we mapped each sig-DMR to the nearest gene and constructed a network using the GeneMANIA Cytoscape plugin (Fig. 6). Interestingly the breast cancer 1 gene (*BRCA1*) stands out as one of the most highly connected node in our network, physically interacting with nine other proteins (Fig. 6). In addition, cysteine rich secretory protein 2 (*CRISP2*), was the gene with the highest node degree. Due to their high network interconnectivity (high node degree), *BRCA1* and *CRISP2* genes were selected for further DNAmethylation biomarker validation studies.

### Verification of Illumina 450 K DNA methylation intensities of *BRCA1* and *CRISP2* DMRs by Epityper MassARRAY

**Figure 7:**
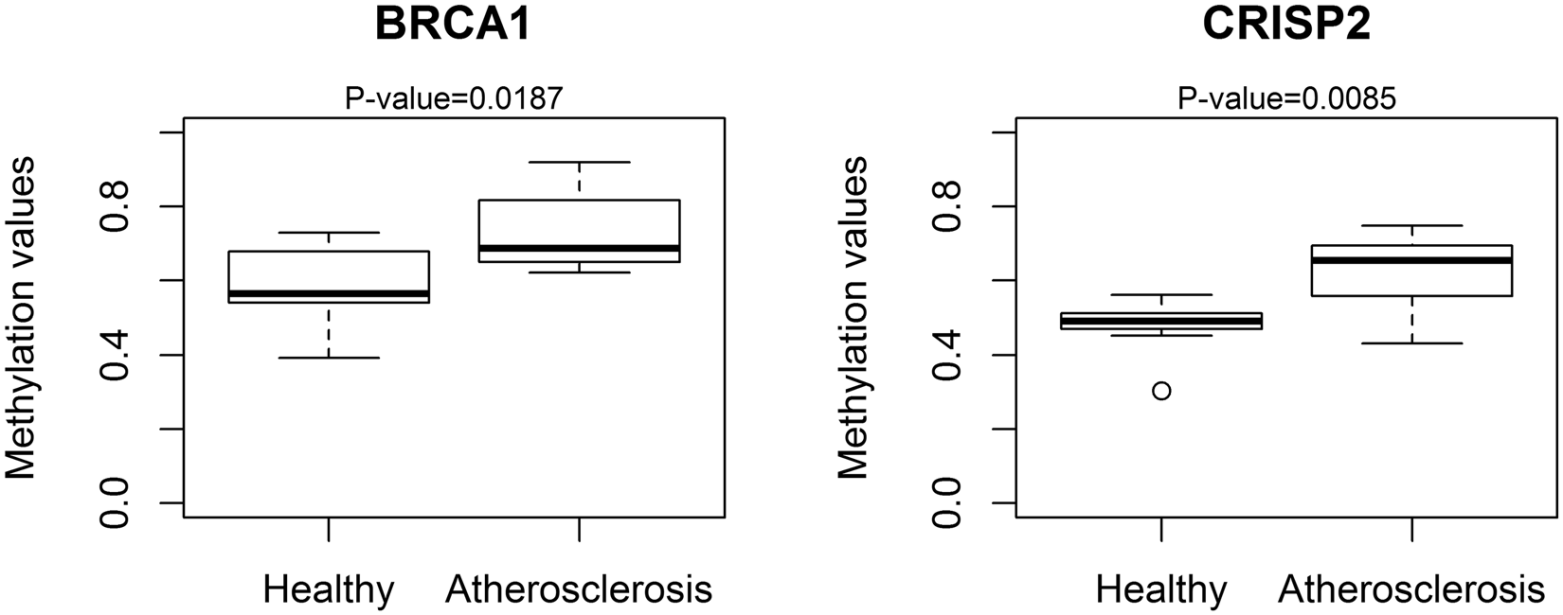
MassARRAY EpiTYPER validation of *BRCA1* and *CRISP2*. The mean methylation values of each measured region are represented in boxplots. The student t-test was used to calculate the significance of the methylation difference between healthy and atherosclerotic blood samples.

Differentially methylated regions of *BRCA1* and *CRISP2* genes determined by Illumina 450 array were selected for further technical validation by Epityper MassARRAY. These regions were found to be significant using the DMRcate package, having multiple CpG sites with more than 10% difference in DNA methylation and showed optimal amplicon fragmentation patterns in Epityper MassARRAY analysis. *BRCA1* and *CRISP2* had the best assay score for MassARRAY analysis, whereas other genes of interest (*EID3, SDHAP3, TSKS and AURKC*) showed limitations in their fragmentation patterns. For *BRCA1*, we decided to focus on 14 consecutive hypermethylated CpG probes located in a CGI (chr17:41,277,974–41,278,445, Supplementary Fig. 2). Nine CpG probes in this region showed more than 10% DNA hypermethylation in the atherosclerosis patients (cg26370022, cg15065591, cg02286533, cg18372208, cg14947218, cg16006004, cg06001716, cg25288140 and cg24900425). The CGI is located in an active promoter region (Roadmap Epigenomics Project^23^). Finally, the promoter region of the *CRISP2* was selected as a second amplicon for validation. Seven neighboring CpG sites (cg26715042, cg14997592, cg04595372, cg01706515, cg25390787, cg08942800 and cg01076129) were found to be more than 10% hypermethylated in this region for the atherosclerosis patients. The amplicon covers the entire promoter region of the gene (Supplementary Fig. 2). Altogether, the Epityper MassARRAY and Illumina DNA methylation levels revealed strongly significant correlations for *BRCA1* (ρ = 0.711–0.932) and *CRISP2* (ρ = 0.680) (Supplementary Fig. 3A). Moreover, *BRCA1* and *CRISP2* target genes demonstrate significant hypermethylation in atherosclerosis blood samples, as compared to blood samples derived from healthy individuals (Fig. 7). Similar DNA atherosclerosis associated *BRCA1* DNA hypermethylation results were also obtained by pyrosequencing of the *BRCA1* DMR chr17:41278125–41278228 (including cg26279233, cg cg06001716 and cg cg14947218), whereas no valid pyrosequencing assay could be designed for the CRISP2 DMR (Supplementary Fig. 3B and data not shown).

We further compared the methylation status of the blood cells types in the two validated regions using the reference methylation data set of Reinius *et al*.^21^. Four CpG probes in the *BRCA1* DMR (cg26370022, cg11529738, 14947218 and cg06001716) (Supplementary Fig. 4) showed significant DNA methylation differences between immune cell types (p < 0.05, not corrected for multiple testing). However, after Bonferroni correction hypermethylation of only one CpG probe remained significantly correlated with immune cell type (cg26370022). In the *CRISP2* DMR significant immune cell type specific DNA methylation changes were observed for two CpG probes (cg01706515 and cg21710255) (Supplementary Fig. 4). However, observed atherosclerosis related DNA hypermethylation trend for most *BRCA1* and *CRISP2* CpG probes does not follow the expected methylation change in blood samples enriched for granulocyte and reduced CD8^+^ T immune cell subpopulations. As such, our results show that DNA hypermethylation of most *BRCA1* and *CRISP2* CpG probes occurs independently of sample variation in blood cell type composition and is associated with atherosclerosis pathology.

**Figure 8:**
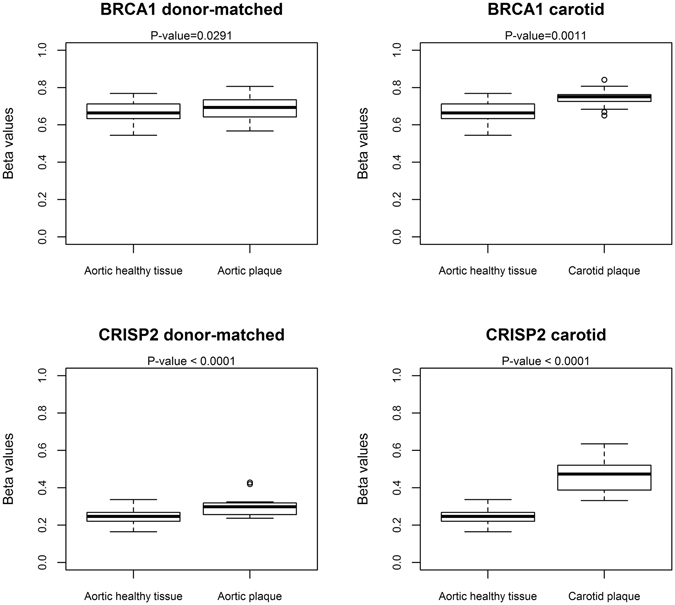
DMRs associated with *BRCA1* and *CRISP2* in an independent publicly available cohort (Zaina *et al*.^22^). The Zaina *et al*. cohort comprises of 30 donor-matched atherosclerotic plaque tissue samples and 19 carotid plaque samples.

As shown in Fig. 8, the same hypermethylation trend of the *BRCA1* and *CRISP2* regions could be replicated in atherosclerotic tissues from the study by Zaina *et al*., both in the carotid plaques and the donor-matched aortic tissues^22^. Altogether, our data suggest that *BRCA1* and *CRISP2* DNA methylation patterns are potential epigenetic biomarkers related to atherosclerosis in blood and atherosclerotic plaque tissue matrix.

### Association of blood DNA methylation changes in *BRCA1* and *CRISP2* with subclinical atherosclerosis in healthy middle age men of the AWHS cohort

To explore the role of validated regions as predictors of subclinical atherosclerosis in a population with a low burden of disease, we evaluated the association of DNA methylation levels from fresh frozen whole blood samples collected at the baseline visit (2009–2010) and subclinical atherosclerosis measured in 2011–2013 from a subsample of 24 Aragon Workers Health Study (AWHS) participants with available baseline InfiniumMethylation 450 K data. We looked for associations between blood DNA methylation and the subclinical atherosclerosis measures, coronary calcium score and carotid intima media thickness. Associations were found for three CpG probes located in *CRISP2* (cg12440062, cg25390787, cg01076129) and 1 CpG probe in *BRCA1* (cg16630982). The strongest statistically significant CpG probe was cg12440062 (located 70 bases upstream of the promotor) for *CRISP2*, consistently, for both coronary calcium score and carotid intima media thickness measures (Table 2). The multi-adjusted difference in coronary calcium score comparing the 75^th^ to the 25^th^ percentiles of DNA methylation was −46.62 score points (−86.87, −6.36; p-value = 0.03) for cg12440062 in *CRISP2*. The corresponding difference in carotid intima media thickness was −0.20 millimeters (−0.33, −0.06; p-value = 0.009) for cg12440062 in *CRIPS2*. For *CRISP2*, DNA methylation in cg01076129 (located in promoter region) was associated with carotid intima-media thickness, but not coronary calcium score. The association with cg25390787 (located in promotor region) was only borderline significant (p = 0.06) consistently for both atherosclerosis measures. With respect to the *BRCA1* DMR, the strongest association with both coronary calcium score (p-value = 0.018) and carotid intima media thickness (p-value = 0.0019) was found for cg16630982 (located in the promotor region), whereas other cg probes did not reach significance within the limited sample series tested.

**Table 2:** Significant associations of *BRCA1* and *CRISP2* CpG probes with coronary calcium score and carotid intima thickness.

CpG probe	Gene	Difference P75vsP25 (95% CI)	P-value
**Coronary calcium score**			
cg16630982	*BRCA1*	−36.59 (−64.53–−8.64)	0.018
cg12440062	*CRISP2*	−46.65 (−86.87–−6.36)	0.03
cg25390787	*CRISP2*	34.98 (−69.35–−0.61)	0.06
**Carotid intima thickness**			
cg16630982	*BRCA1*	−0.16 (−0.25–−0.07)	0.0019
cg12440062	*CRISP2*	−0.20 (−0.33–−0.06)	0.009
cg01076129	*CRISP2*	−0.14 (−0.26–−0.02)	0.036
cg25390787	*CRISP2*	−0.12 (−0.24−0.00)	0.06

## Discussion

In this study, we identified genomic regions in *BRCA1* and *CRISP2* which were consistently differentially methylated in blood DNA of atherosclerosis patients compared to healthy individuals, and in aortic and carotid plaque samples compared to aorta samples without plaque. Furthermore, methylation in *BRCA1* and *CRISP2* DMR was also associated with subclinical atherosclerosis measures in an independent sample of middle age men. Our results thus support a potential role of blood DNA surrogate markers for early CVD detection.

Epigenetics may provide the missing mechanism linking environment, genome and atherosclerotic phenotype. Identifying epigenomic biomarkers that parallel the development of subclinical atherosclerosis might open new paths for risk stratification and prevention, and may help to further understand the pathophysiology of atherosclerosis. In particular, changes in DNA methylation patterns have been linked to several cardiovascular-related biomarkers, including homocysteine and C-reactive protein^9^. Furthermore, an increasing number of studies report DNA methylation alterations in atherosclerosis^24–26^. For example, a recent genome-wide study showed DNA methylation differences between healthy donor-matched aortic healthy and plaque tissue, indicated by epigenetic drift of DNA methylation in aortic plaques with atherosclerotic progression^22, 27^. Furthermore, known cardiovascular risk factors, including homocysteine levels, smoking and age have been described to induce DNA methylation changes^28–30^. Currently, only few DNA methylation studies have been performed with blood samples of CAD patients. Sharma *et al*. identified 72 hypermethylated DMRs associated with CAD and hyperhomocysteinemia using a 12k human CpG island microarray^31^. Guay *et al*. performed a study on subjects with familial hypercholesterolemia with or without CAD using the Infinium 27k methylation array^32^. Since blood leukocytes have major contributions to the initiation, progression and maintenance of atherosclerosis, we determined genome-wide DNA methylation profiles in blood samples of atherosclerosis patients, in comparison to healthy individuals to identify CVD related epigenetic biomarkers. Although Illumina 450 K profiling did not reveal significant global DNA methylation differences between atherosclerosis patients and healthy individuals, HPLC based methods demonstrated global DNA hypermethylation in blood leukocytes^9, 33^ whereas both global DNA hypermethylation and hypomethylation have been reported in atherosclerotic vascular tissue^22, 34, 35^.

Upon further mapping of DNA methylation changes at specific CpG motifs or regions, 161 DMRs were identified to be differentially methylated based on specific selection criteria. Of particular interest, pathway enrichment analysis of gene associated DMRs revealed DNA impaired epigenetic regulation of integrin and cadherin dependent cell adhesion, cell cycle, cell death, chemotaxis, immune phagocytosis and estrogen hormone pathways which are all critically involved in atherosclerosis. In accordance with other studies examining DNA methylation in metabolic diseases, the observed methylation changes were relatively small (max 20%), as compared to cancer specific DNA methylation changes. More specifically, whereas promoter regions and CpG islands were found to be depleted of atherosclerosis associated DMPs, gene bodies, intergenic and open sea regions show enrichment of various DMPs. Interestingly, a strong enrichment was also observed in enhancer regions suggesting that impaired control of distal regulatory regions may contribute to atherosclerosis.

Remarkably, mathematical deconvolution (Houseman correction) of the blood DNA methylation profiles revealed significant changes in the granulocyte and CD8^+^ T immune cell populations in atherosclerosis patients as compared to healthy individuals, which could be highly relevant for atherosclerotic plaque formation. More particularly, important regulatory roles for granulocyte and CD4^+^/CD8^+^ T cell populations have recently been identified in atherosclerotic lesions and coronary thrombus evolution^36, 37^. As expected, a large fraction of the sig-DMRs were also differentially methylated between blood cell types, suggesting that their change in DNA methylation in atherosclerosis patients could be due to a difference in blood cell type composition between atherosclerosis and healthy controls. Heterogeneity of blood samples could be prevented using cell count and sorting methods (FACS) to analyze specific immune cell subpopulations. However, these methods are difficult and costly to apply in large epidemiological cohort studies. CpG sites associated with blood cell type were excluded for further analysis, leaving 110 sig-DMRs not affected by blood cell types.

Interestingly, of the 110 remaining DMRs (comprising 497 CpG sites), 34 DMRs (69 CpG sites) were also found to be differentially methylated in plaque tissues, which suggests that blood-associated epigenetic biomarkers can be valid surrogate markers for methylation changes in plaque material. Of particular interest, pathway enrichment analysis of the 34 common gene associated DMRs revealed epigenetic impairment of *NRF2* oxidative stress (*SOD2*), DNA repair (*BRCA1*), thioredoxin (*TXNRD1*) and inflammatory pathways (*MIF*, *PLA2G4D*) in atherosclerosis conditions. Upon further network analysis of each sig-DMR, mapped to the nearest gene, we identified a highly interconnective network with central roles of *BRCA1* and *CRISP2* DMRs, which prompted us to focus on these genes for further validation. Of special note, the differentially methylated regions in *BRCA1* and *CRISP2* appeared to be largely independent of age and/or immune cell type composition, and both hold promise as valuable atherosclerosis related biomarkers in routine blood analysis. Interestingly, DNA methylation at the *BRCA1* locus was already present in healthy controls, which is contrary to other studies where a lack of DNA methylation was observed^38, 39^. Nevertheless, it must be emphasized that the methylated CpGs in our data set are located in a CpG island approximately 600 bp upstream of the *BRCA1* gene, whereas previous studies^40^ detected hypomethylation around the transcription start site of the gene, which can also be appreciated in our data (Supplementary Fig. 2).

DNA methylation changes of the *BRCA1* and *CRISP2* DMRs could also be replicated in an Illumina 450 K dataset of paired atherosclerotic plaque and normal aorta samples from 24 middle aged men with subclinical atherosclerosis of the Aragon Workers Health Study (AWHS). Moreover, we also observed statistically significant association between DNA methylation in several CpG sites in these regions and coronary calcium score and carotid intima-media thickness when using data from this well-established population-based cohort^3, 16^, thus adding robustness to our findings. Surprisingly, the associations with subclinical atherosclerosis measures were not always directionally consistent compared to the associations comparing blood DNA methylation of cardiovascular disease versus healthy individuals or the atherosclerosis versus normal aorta samples. The explanation for this inconsistency still remains unclear, although we cannot exclude cell type specific variations in blood sample composition or complex genotype SNP, microRNA or lncRNA dependent heterogenic epigenetic regulation of different *BRCA1* or *CRISP2* variants^41–47^. In addition, changes in lifestyle (diet, smoking, pollution, exercise) and pharmacological treatments (statins, aspirin, PARP inhibitors) could further obscure DNA methylation changes associated with atherosclerosis^47, 48^.

However, the known biological role of *BRCA1* and *CRISP2* in cardiometabolic risk and inflammation pathways adds further significance to our findings. Besides the well described tumor suppressor function of *BRCA1* in breast and ovarian cancers, more recent research also demonstrates an important role for *BRCA1* in suppression of endothelial dysfunction and atherosclerosis^49^. In the latter study, *BRCA1*-overexpressing ApoE knock-out mice developed significantly less atherosclerotic plaque lesions together with reduced macrophage infiltration and diminished ROS production^49^. In another study, women lacking functional *BRCA1/BRCA2* at increased breast cancer risk also show greater risk for heart disease and metabolic diseases^50–53^. *BRCA1* is involved in multiple cellular processes and genome stability maintenance like DNA repair, transcriptional regulation, ubiquitination and cell-cycle control^54^. Excessive production of reactive oxygen species, in part via upregulation of DNA damage pathways, is a central mechanism governing pathologic activation of vascular smooth muscle cells. Remarkably, *BRCA1* was found to shield vascular smooth muscle cells (VSMCs) from oxidative stress by inhibiting NADPH Nox1-dependent reactive oxygen species production^55^. More recently, *BRCA1* was found to regulate lipogenesis through its interaction with acetyl coenzyme A carboxylase^56^. Along the same line, *BRCA1* plays a critical role in the regulation of metabolic function in the skeletal muscle where it is involved in lipid storage and insulin resistance^57^. In analogy to *BRCA1* dependent suppression of cell motility and epithelial-mesenchymal transition in cancer^58^, *BRCA1* may also prevent endothelial-mesenchymal transition involved in atherosclerosis progression and other CVDs (myocardial infarction, vascular calcification)^59–61^. As our data suggests, silencing of the *BRCA1* gene in atherosclerosis patients may mean that this gene not only acts a tumor suppressor but also as a vascular protector against oxidative cell damage.

While no *CRISP2* functions have so far been reported in relation to CVDs, *CRISP2* gene activities were recently associated with oxidative stress responses and decline of lung function upon smoke or particular matter exposure^62^. In another study, CRISP expression was found to abolish the neovascularization process induced by exogenous growth factors (bFGF, vpVEGF)^63^. Decreased *CRISP2* expression correlated with Th2-like eosinophilic inflammation in chronic nasal asthmatic chronic rhinosinusitis^64^. As such, the potential involvement of *CRISP2* in CVD pathologies and angiogenesis via oxidative stress and inflammatory responses warrants further investigation.

An important limitation in our study is reflected by the small sample size of the studied samples, which renders our analysis clearly underpowered. Future prospective studies in larger and distinct cohorts could further enable the validation of *BRCA1* and *CRISP2* to predict early cardiovascular disease. Additionally, the atherosclerosis patients were older than the healthy controls. Even though, a correction for age-specific methylation was performed, we cannot exclude that age may affect the results and is therefore a confounding factor. In addition, as atherosclerosis is an age-dependent disease, probably some of the excluded age-dependent CpG sites overlap with CDV related CpGs. Nonetheless, in the post-hoc analysis with the Aragon Workers Health Study, a study population composed of cardiovascular disease free middle age men, the main findings were consistent even after adjustment of age, BMI, smoking and houseman cell composition. While we cannot discard a potential lack of generalizability, which is typical of observational studies, an important strength of our study includes the availability of DNA-methylation data from three independent set of samples covering the whole spectrum from blood samples from individuals with subclinical atherosclerosis measures and individuals at high and low cardiovascular risk to carotid and aorta samples.

In conclusion, we identified promising novel epigenetic biomarkers of clinical and subclinical atherosclerotic disease, in genes involved in impaired leukocyte-endothelium functions during atherosclerosis progression. These regions deserve further consideration in experimental studies and prospective population-based cohort to confirm their potential role in cardiovascular risk. If confirmed, the reported markers could become potential tools to support early identification of individuals at high CVD risk who could benefit from preventive interventions for cardiovascular disease prevention and control.

## Methods

We fist conducted a discovery phase analysis of DNA methylation data from 8 healthy voluntaries and 8 patients with atherosclerosis from the Study “Impact of Left Ventricular Assist Devices Implantation on Micro- and Macrovascular Function” (LVAD study, clinicaltrials.gov: NCT02174133). Subsequently we validated findings from the discovery phase by analyzing DNA methylation data from plaque material related to GEO dataset GSE46401 published by Zaina *et al*. Finally, as a post-hoc analysis, we explored the potential role of the identified markers as early detection biomarkers by evaluating the association of DNA methylation and subclinical atherosclerosis endpoints in whole blood DNA from 24 Aragon Workers Study participants free of clinical cardiovascular disease.

### Experimental set-up and sample collection in the discovery stage

In the LVAD study, eight healthy volunteers were recruited based on following inclusion criteria: age (35–60 years), BMI (23–27 kg/m²), average physical activity and normal western diet (clinicaltrials.gov: NCT01799005). The exclusion criteria for the healthy volunteers were CVD, diabetes mellitus, acute inflammation and arrhythmia. We additionally selected eight patients with confirmed clinical diagnosis of atherosclerosis (clinicaltrials.gov: NCT02174133). The characteristics of the study population are described in Table 1. Whole blood (0.5 ml) was collected from all individuals, following informed consent. The study was conducted according to the guidelines laid down in the Declaration of Helsinki and all procedures involving human subjects were approved by the University of Düsseldorf Research Ethics Committee (ref: 3870 and ref: 4565R).

### Infinium HumanMethylation450 BeadChip Array processing and data analysis for whole blood DNA from atherosclerosis patients and healthy donors

Genomic DNA (gDNA) isolated from 0.5 ml whole blood (EDTA), was isolated with DNeasy Blood & Tissue kit (Qiagen Hilden, Germany) and quantified by Nanodrop™ spectrophotometry. 1000 ng of gDNA was bisulfite converted using the EZ DNA methylation kit (Zymo Research, Orange, CA, USA) according to manufacturer’s instructions. Genome-wide DNA methylation was analyzed on Infinium HumanMethylation450 BeadChip platform (Illumina, San Diego, CA, USA) at the DKFZ Genomics and Proteomics Core Facility. 4 µl of bisulfite-converted whole blood DNA (~150 ng) was used for the whole genome amplification (WGA) reaction, enzymatic fragmentation, precipitation and re-suspended in hybridization buffer. Subsequent steps of DNA methylation analysis were carried out according to the standard Infinium HD Assay Methylation Protocol Guide (Part #15019519, Illumina). The BeadChip images were captured using the Illumina iScan. Pre-processing and analysis of the Infinium 450k data was performed using the R package RnBeads^65^. CpG probes containing a SNP at least 3 bp from the 3’ query site, having a detection p-value higher than 0.01, having empty values in at least one sample or measuring methylation in a non-CpG context were removed. In total 8,533 CpG probes (1.75%) were filtered. Intra-array normalization was done using the Beta Mixture Quantile Normalization^66^. Methylation values were represented as β-values ranging from 0 to 1. β-alues were converted into M-values $(M\,=\,{\rm{l}}{\rm{o}}{\rm{g}}2(\frac{\beta }{(1-\beta )}))\,$before doing the statistical analysis. Limma R package was used to identify differentially methylated positions. Raw p-values were corrected for multiple testing using the Benjamini-Hochberg method. CpG probes with an adjusted p-value below 0.15 and having a difference in β-values of at least 0.05 (i.e. 5% difference in DNA methylation) between atherosclerosis patients and healthy controls were denoted as significant, and named sig-DMPs. Differentially methylated regions were identified using the DMRcate R package^67^. A region was called significant when Pmean-value was below 0.001 with a maximum methylation difference of at least 5% and containing at least five CpGs. Sig-DMPs were annotated using the HumanMethylation450 v1.2 manifest file. The freely available EpiExplorer tool was used to add further annotation including chromatin state segmentation and histone modifications^17^. Enrichment or depletion of sig-DMPs in a particular genomic region was determined using the Fisher’s exact test. Commercial Metacore (https://portal.genego.com/)and Ingenuity (www.ingenuity.com/) software packages werre used to identify significant pathway enrichment of gene associated DMRs.

The method of Houseman *et al*.^20^ incorporated in the RnBeads package was used to estimate the cell type composition in blood. Reference cell types for granulocytes, CD4+ T-cells, CD8+ T-cells, B-cells, monocytes and NK-cells were obtained from the study of Reinius *et al*.^21^ using the FlowSorted.Blood.450k R package. The methylation profiles were processed (filtering and normalization) together with the atherosclerosis methylation dataset in the same way as described above. In total 50,000 CpG probes with the highest variance were used to identify the top 500 CpG probes associated with the cell types. The relative cell type contributions were compared between healthy individuals and atherosclerosis using a normal student t-test. One-way ANOVA and Bonferroni Post-hoc test was used to detect methylation differences between the blood cell types using the data from Reinius *et al*.

### Replication in atherosclerotic plaque material methylation dataset GSE46401

Normalized Infinium 450k DNA methylation data of atherosclerotic plaque material were obtained from GEO dataset GSE46401. The dataset contains data from 15 donor-matched aorta healthy and plaque tissue and from 19 carotid plaque material. A paired two-tailed student t-test was performed to find DNA methylation differences in the 15 donor-matched samples and an unpaired two-tailed student t-test was performed to find DNA methylation differences between the carotid plaque tissue samples and the healthy aorta samples.

### Epityper Sequenom MassARRAY

*In silico* cleavage was done by means of the RSeqMeth script in R to aid selection of an optimal primer set for the genomic region of interest^68^. MassARRAY primers for regions in the *BRCA1* (chr17:41,277,701–41,278,776) and *CRISP2* (chr6:49,680,757–49,682,289) genes (Supplementary Table 1 and Supplementary Fig. 2) were designed using the Sequenom EpiDesigner online tool (www.epidesigner.com). Bisulfite converted DNA was used for the methylation analysis. PCR reactions were performed using the following reagents: 10x buffer (Qiagen®), 10 mM dNTP, 10 µM primer mix, 5 U/µl HotStarTaq^TM^ polymerase (Qiagen®) and deionized water. Methylation percentages were calculated based on the ratio of the unmethylated versus methylated peaks. In addition, DNA methylation standards (0, 20, 40, 60, 80 and 100%) were used to control for amplification bias. The R computing environment was used for the correction of the obtained methylation data according to standard procedures^69^. Linear regression was performed to fit the obtained data points according to the predicted standard methylation values. The student T-test was used to calculate the significance of the methylation difference between healthy and atherosclerotic blood samples.

### Pyrosequencing

1 µg gDNA from each sample was bisulfite converted using the EpiTect Fast bisulfite conversion kit (Qiagen, Hilden, Germany) according to manufacturer’s instructions. 15 ng of bisulfite treated DNA was subsequently used in PCR amplification using the PyroMark PCR kit (Qiagen, Hilden, Germany). Reverse primers were biotinylated to get biotin-labelled PCR products. Finally, DNA sequences were pyrosequenced using the PyroMark Q24 Advanced instrument (Qiagen, Hilden, Germany). First, streptavidin-coated Sepharose beads (High Performance, GE Healthcare, Uppsala, Sweden) were used to immobilize the biotin-labelled PCR products. Subsequently, PCR products were captured by the PyroMark vacuum Q24 workstation, washed and denaturated. The single stranded PCR products were mixed and annealed with their corresponding sequencing primer. After the pyrosequencing run was finished, the results were analysed using the PyroMark Q24 Advanced software (Qiagen, Hilden, Germany). Biotinylated-reverse, forward and sequencing primers were designed using the PyroMark assay design 2.0 software (Qiagen, Hilden, Germany) (Supplementary Table 1).

### Post-hoc analysis of human blood DNA methylation in BRCA1 and CRISP2 and subclinical atherosclerosis in middle-age healthy men

The Aragon Workers Health Study (AWHS) is a study designed to assess cardiovascular risk and subclinical atherosclerosis in a cohort of middle-aged healthy men from Spain. The AWHS design and baseline characteristics have been reported elsewhere^3, 16^. In brief, in the baseline examination (2009–2010), the average (SD) age, body mass index, and waist circumference were 49.3 (8.7) years, 27.7 (3.6) kg/m2 and 97.2 (9.9) cm, respectively, The prevalence of overweight, obesity, current smoking, hypertension, hypercholesterolemia, and diabetes were 55.0, 23.1, 37.1, 40.3, 75.0, and 7.4%, respectively^70^. The adherence of the AWHS participants to the Mediterranean diet has been extensively studied^71^. 21.7% of participants in the AWHS reported being physical active (e,g, >150 min/week or 30 min/d of jogging, walking quickly, dance, aerobics, gardening)^71^. The levels of physical activity were positively associated with the adherence to the Mediterrean lifestyle^71^. In 2011–2013, calcium coronary scoring was performed using non-contrast ECG gated prospective acquisition by a 16 multidetector computed tomography scanner (Mx 8000 IDT 16, Philips Medical Systems, Best, the Netherlands). During a single breath hold, images were acquired from the tracheal bifurcation to below the base of the heart. Scan parameters were 8 × 3 mm collimation, 220-mm field of view, 120 kVp, 55 mA, and 3-mm section thickness. Coronary calcium was quantified with calcium scoring software (Workspace CT viewer, Philips Medical Systems) that follows the Agatston method^72^. Carotid intima-media thickness was determined using the Philips IU22 ultrasound system (Philips Healthcare, Bothell, Washington). Ultrasound images were acquired with linear high-frequency 2-dimensional probes (Philips Transducer L9–3, Philips Healthcare), following the Bioimage Study protocol^73^. Examination of the carotid territory included the terminal portion (10 mm) of the common carotid, the bulb, and the initial portion (10 mm) of the internal and external carotid arteries. The given value for carotid artery intima-media thickness is the mean value from all sites at both sides. The AWHS study was approved by the Ethics Committee for Clinical Research at the Institutional Review Board of Aragón (CEICA)^3, 16^. All study participants provided written informed consent. The methods for DNA isolation and bisulfite conversion were similar to the methods implemented in the LVAD samples, which are standard manufacturer procedures. DNA methylation was measured using the platform Illumina Infinium Methylation 450 K in a subsample of 23 individuals with available measures of subclinical atherosclerosis. Preprocessing and analysis of the Infinium 450k data was performed using the R package minfi^74^. CpG probes with a detection p-value higher than 0.01 were removed. Intra-array normalization was done using the Quantile Normalization. In exploratory analysis, we detected a potential batch effect by slide. Methylation proportion values were represented as β-values ranging from 0 to 1. β-values were converted into M-values before doing the statistical analysis. For analysis of site-specific DNA methylation (independent variable) and subclinical atherosclerosis measures (dependent variable) in the AWHS, we estimated the differences in coronary artery score and carotid intima media thickness comparing 75^th^ versus 25^th^ percentiles of DNA methylation distribution at a given CpG site by linear regression with the following adjustment variables: age, smoking status (never, former and current smoking), body mass index, and houseman cell estimates (B cell, CD4^+^ and CD8^+^ T cells, granulocytes, monocytes and natural killer cells). Due to the small sample size we tried to avoid non-parsimonious regression parameters by performing two-stage regression for adjustment. First we adjusted DNA methylation M-values for potential confounders using combat^75^ to correct for batch effect by slide. Subsequently, we adjusted intima media thickness and coronary artery calcium score levels for the same set of potential confounders. Second, we ran the final regression models using the residuals resulting from the first step recalibrated to the corresponding marginal mean. Since this was post-hoc analysis we evaluated the association of DNA methylation and subclinical atherosclerosis in CpG sites from regions validated in previous analysis (i.e. *BRCA1* and *CRISP2*). Thus, we considered the non-Bonferroni corrected p-values < 0.05 as statistically significant.

### Availability of data

Data are available on request. DNA methylation data will be deposited in the GEO profile database.

### Ethics approval and consent to participate

The study was conducted according to the guidelines laid down in the Declaration of Helsinki and all procedures involving human subjects were approved by the University of Düsseldorf Research Ethics Committee (ref: 3870 and ref: 4565 R). The Aragon Workers Health Study participants (AWHS) study was approved by the Ethics Committee for Clinical Research at the Institutional Review Board of Aragón (CEICA)^3, 16^. All study participants provided written informed consent.

## Electronic supplementary material

Supplementary Figures and Tables
