# Supplementary material for: Identification of differentially methylated BRCA1 and CRISP2 DNA regions as blood surrogate markers for cardiovascular disease
Source: Sci Rep. 2017 Jul 11;7:5120. doi: 10.1038/s41598-017-03434-0 (PMC5506022; doi:10.1038/s41598-017-03434-0)
Supplement: Supplementary file 1 — Supplementary Figures and Tables [file 41598_2017_3434_MOESM1_ESM.pdf]

Supplementary information (Figures and Tables)

Identification of differentially methylated BRCA1 and CRISP2 DNA regions as blood surrogate markers for cardiovascular disease

Geoffrey Istas[1,2,3#], Ken Declerck[1#], Maria Pudenz[4], Katarzyna Szarc vel Szic[1,5], Veronica Lendinez-Tortajada[6], Montserrat Leon-Latre[7,8], Karen Heyninck[9], Guy Haegeman[9], Jose A. Casasnovas,[8,10,11] Maria Tellez-Plaza,[12] Clarissa Gerhauser[4], Christian Heiss[2], Ana Rodriguez-Mateos[2,3], Wim Vanden Berghe[1,9*]

Supplementary Fig. 1

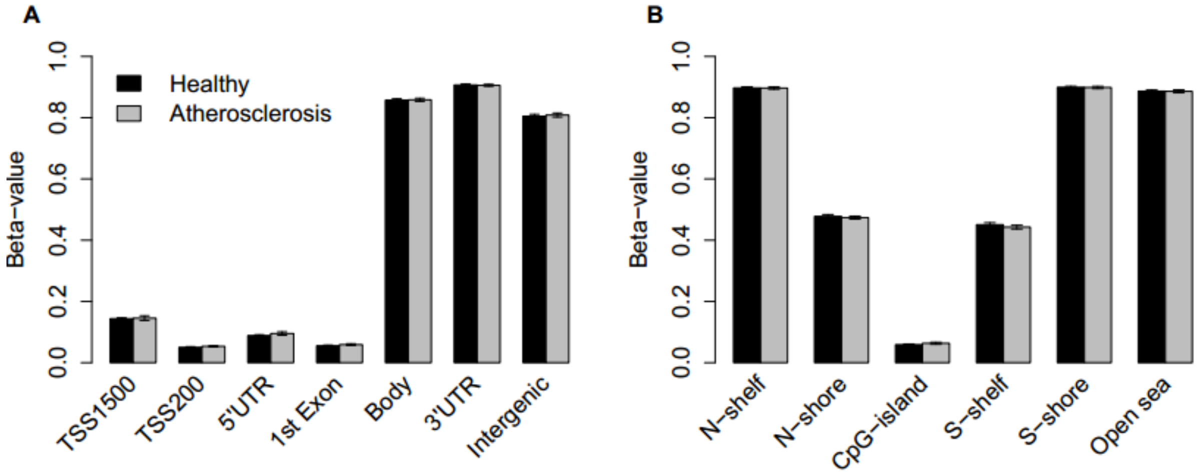

Supplementary Fig. 1 Representation of global DNA methylation of all significant CpG sites observed in patients at high risk for CVD and in healthy individuals based on A) gene regions and B) CpG-island regions. Bars (+/- SEM represent the mean global DNA methylation in each group. No statistical differences were observed (Student t-test p-value > 0.05).

Supplementary Fig. 2

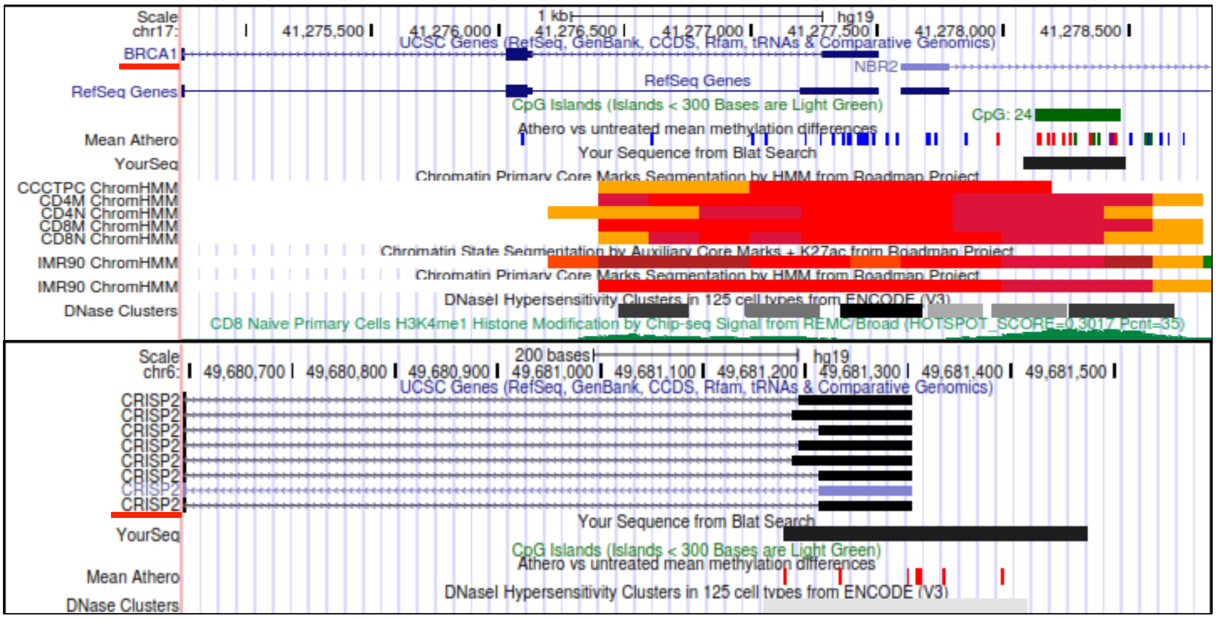

Supplementary Fig. 2 UCSC tracks mapping the selected CGI upstream of the BRCA1 gene (above) and the CRISP2 gene (below). The black YourSeq track represents the location of the amplicon for MassARRAY validation. The mean DNA methylation differences between the test groups were calculated per CpG probe and mapped to UCSC by developing custom tracks (under CpG island track). The red color of the Mean Athero tracks represent DNA methylation differences higher than 10% between two groups. The blue color indicates DNA methylation differences below 5%. Further down, Roadmap Epigenomics Project tracks are shown. Active promoter regions based on histone codes are indicated in red. The last track represents one example of an active histone modification (H3K4me1) generated by Roadmap Epigenomics.

Supplementary Fig. 3

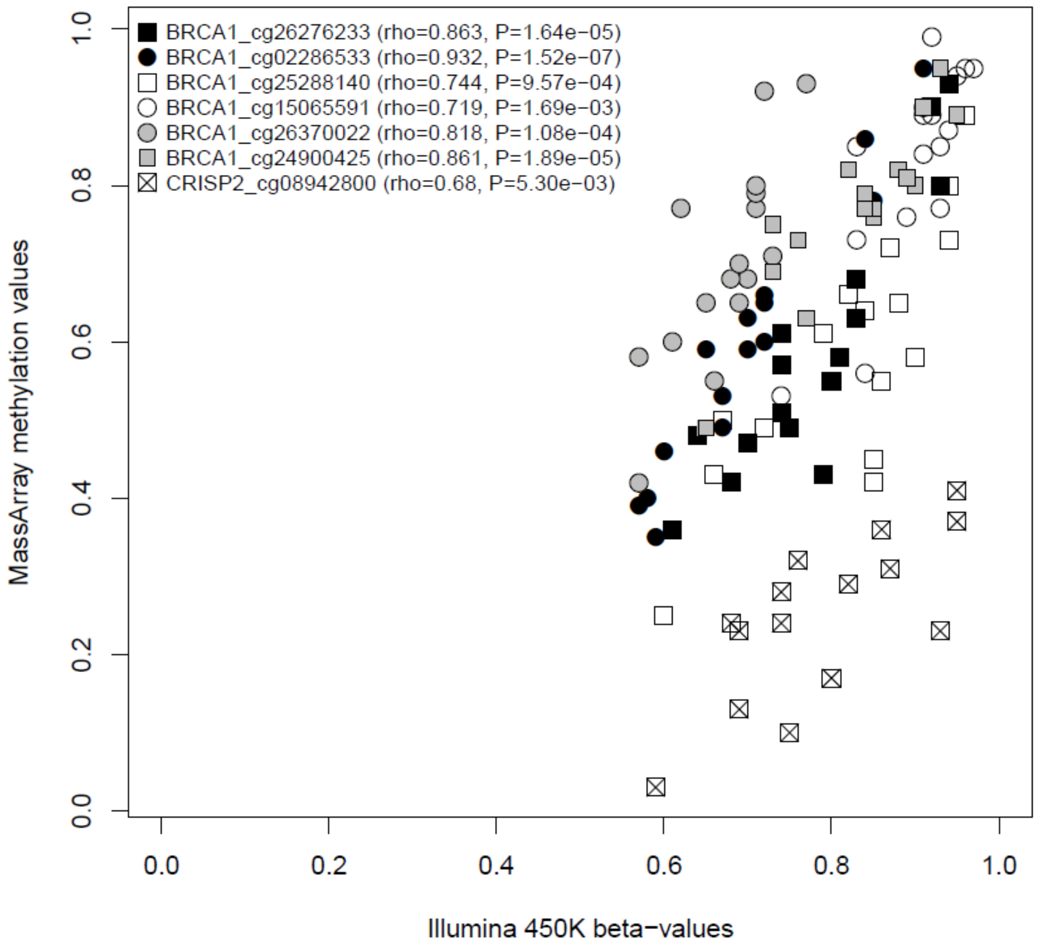

Supplementary Fig. 3A Correlation between Illumina beta-values and MassArray methylation values. Only Illumina probes are represented of which methylation was measured by a single region in the MassArray. For each gene Spearman's correlation coefficient was calculated.

Supplementary Fig.3B Pyrosequencing verification of differential methylation of 7 CpG motifs in the BRCA1 DMR in healthy individuals versus atherosclerosis patients. The pyrosequencing assay covers chr17:41278125-41278228, including 7 neighboring CpG motifs, covering 4 cg probes of the Illumina 450K array; CpG 1: cg26279233, CpG 2: cg06001716, CpG 3, CpG 4, CpG 5: cg02286533, CpG 6: cg14947218, CpG. Statistical difference in CpG specific DNA methylation difference between healthy individuals and atherosclerosis was calculated by a student t-test.

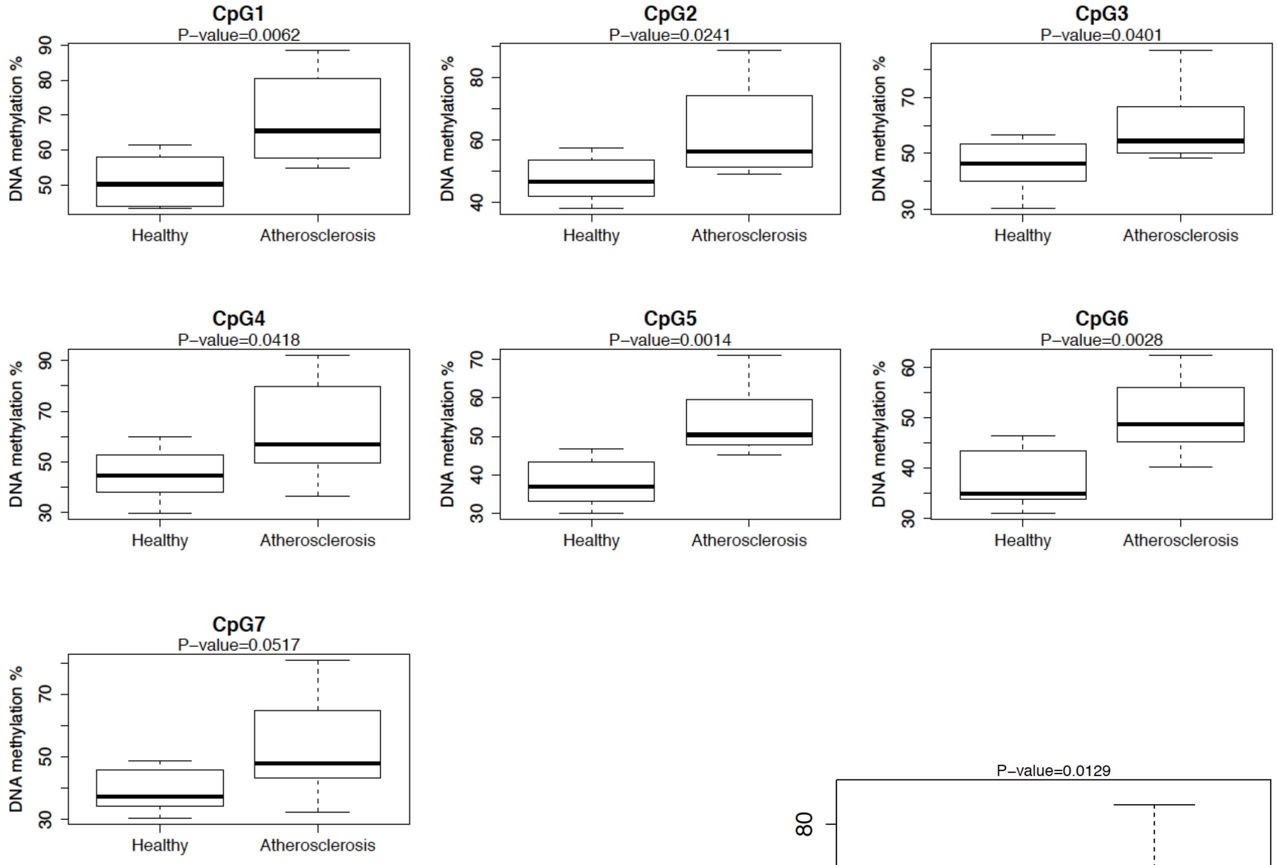

Supplementary Fig. 3C Pyrosequencing verification of mean DNA methylation difference of the BRCA1 DMR (chr17:41278125-41278228) between healthy individuals and atherosclerosis patients. Statistical difference in mean DNA methylation difference was calculated by a student t-test.

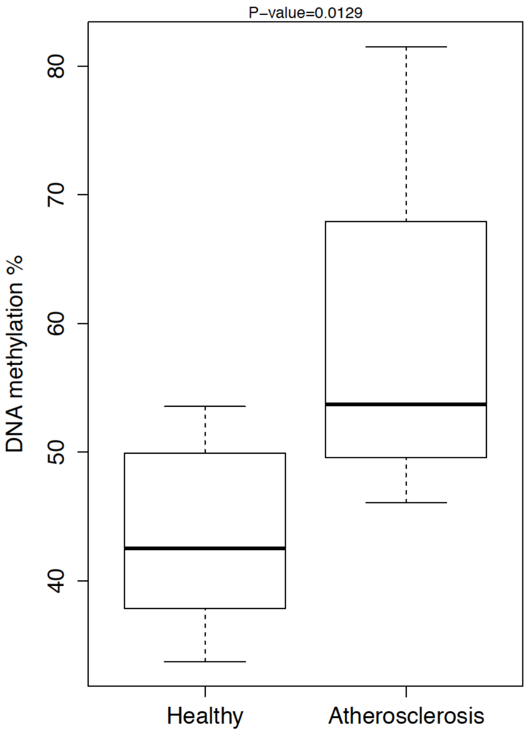

Supplementary Fig. 4.

Supplementary Fig. 4A Methylation of BRCA1 DMR in different blood cell types. Arrows indicate the CpG probes with significant methylation differences (p-value < 0.05) between the different blood cell types, determined by one-way ANOVA.

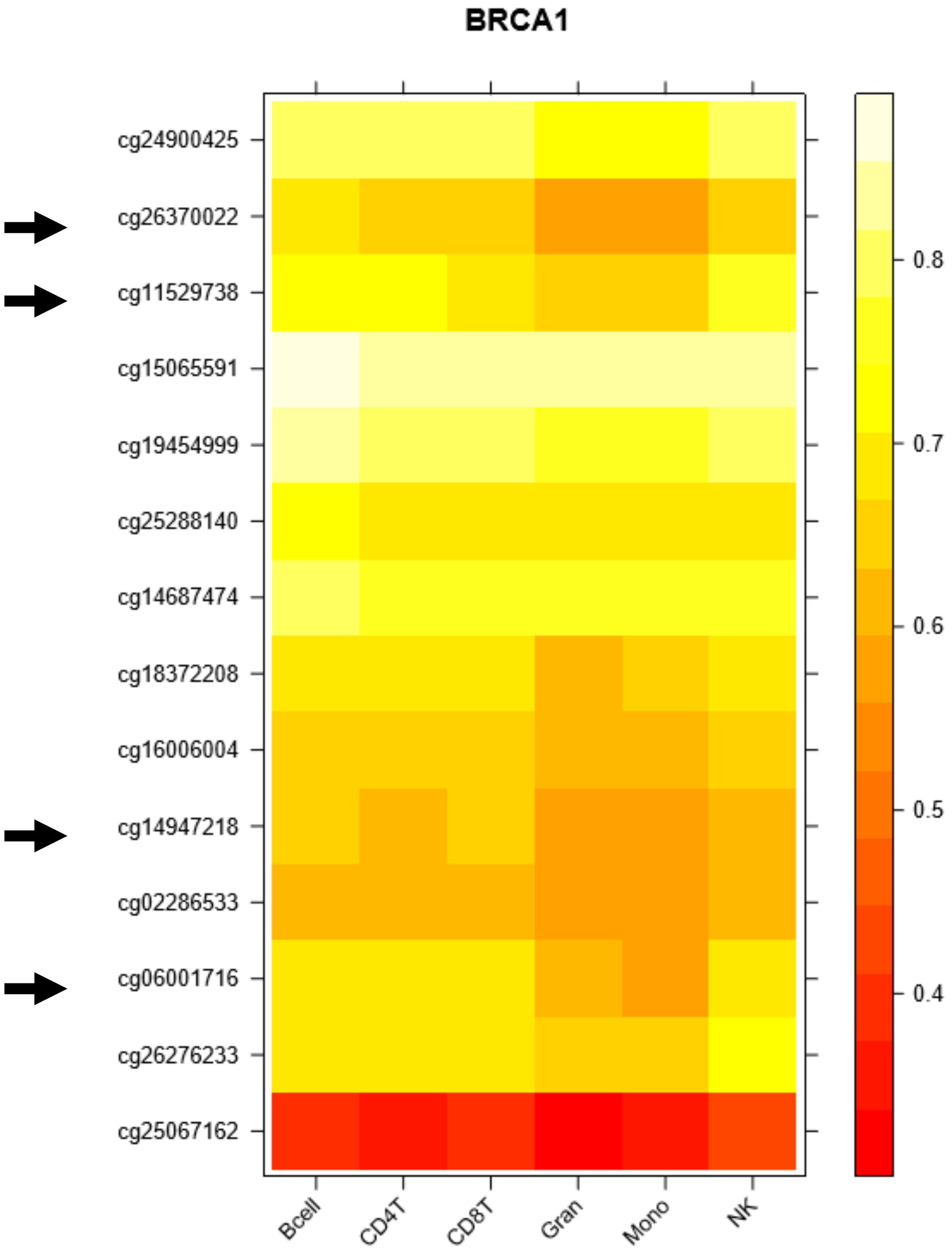

Supplementary Fig. 4B Methylation of CRISP2 DMR in different blood cell types. Arrows indicate the CpG probes with significant methylation differences (p-value < 0.05) between the different blood cell types, determined by one-way ANOVA.

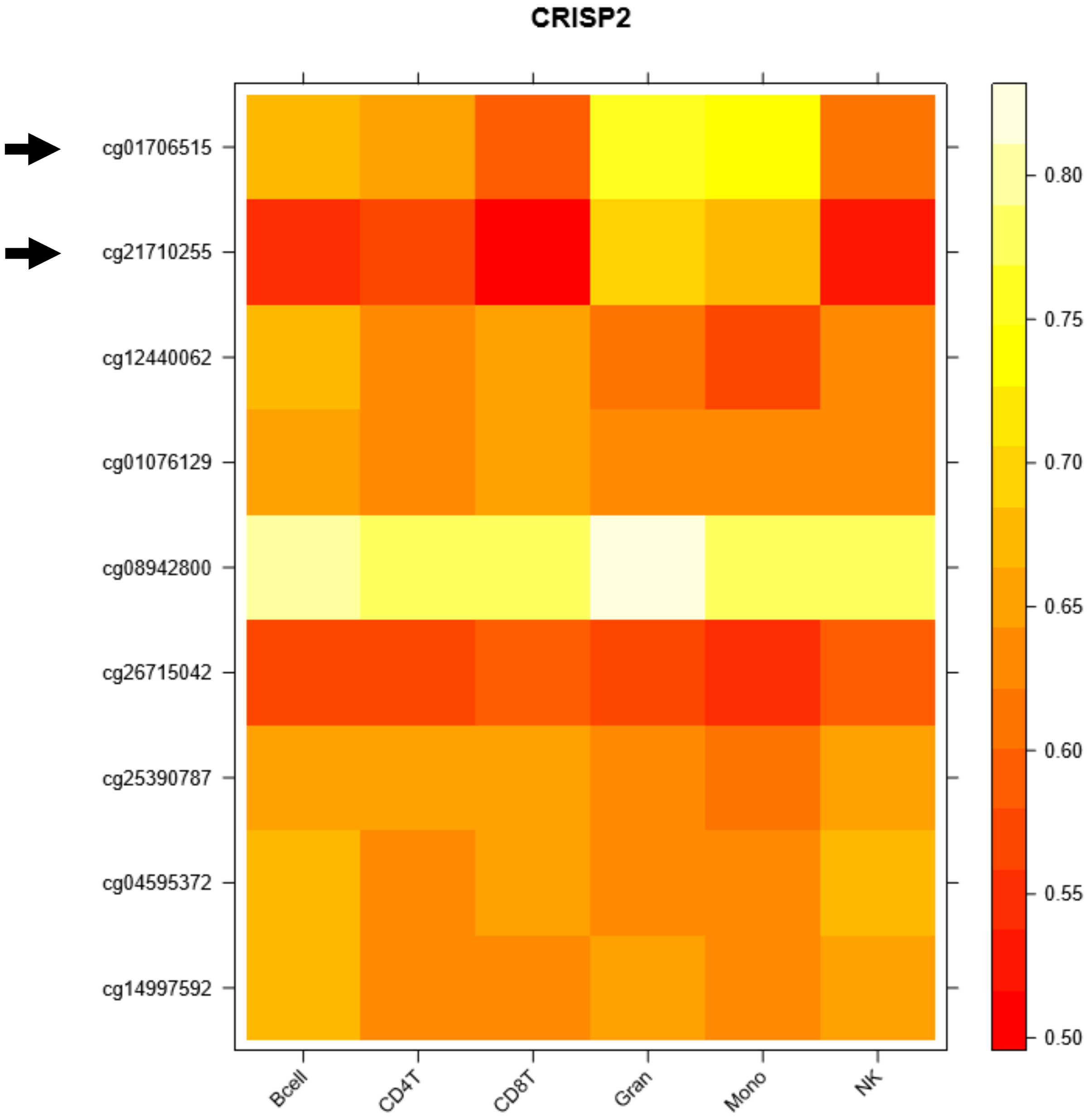

Supplementary table 1: Primer sequences.

Epityper Massarray primers

BRCA1-for	5'-aggaagagagTTTATTTAGTTTTAGGAGTTTGGGG-3'
BRCA1-rev	5'-cagtaatacgactcactatagggagaaggctCAAACTACTTCCTTACCAACTTCCC-3'
CRISP2-for	5'-aggaagagagGGGTTTTATATTTATTTTGGTTTTAGA-3'
CRISP2-rev	5'-cagtaatacgactcactatagggagaaggctCATTTTCTTTCCTCATCAACTAATAA-3'

Pyrosequencing primers

BRCA1_1_FW	GAG-TTT-GGG-GTA-AGT-AGT-TTT-GTA-AG
BRCA1_1_REV(biotin)	CCA-AAA-AAA-TTA-ACT-CTT-ACC-ACT-TAT-CC
BRCA1_1_SEQ	GGT-AAG-TAG-TTT-TGT-AAG-GT

Supplementary table 2: Significant differentially methylated positions (sig-DMPs). FDR < 0.15 and Delta_beta > 5%.

Illumina_probe	Chrom	Start	End	P.Value	FDR	Mean_beta_Athero	Mean_beta_Healthy	Delta_beta	UCSC_RefG	UCSC_RefG	UCSC_RefG	Relation_tc	Age
cg23919111	chr14	95983255	95983256	7,27E-08	0,020154935	0,063679005	0,126895616	-0,063216612				Island	No
cg23732483	chr3	48965610	48965611	8,45E-08	0,020154935	0,087729557	0,200795889	-0,113066332	ARIH2	NM_00632	Body		Yes
cg03725309	chr1	#########	#########	1,42E-07	0,022542547	0,081244973	0,167320409	-0,086075436	SARS	NM_00651	Body	S_Shore	Yes
cg13777513	chr15	65127740	65127741	2,66E-07	0,031673648	0,898406772	0,798958143	0,099448629				N_Shore	No
cg01448562	chr3	#########	#########	3,95E-07	0,037678121	0,966438424	0,906309438	0,060128985				Island	No
cg01908508	chr2	87569141	87569142	5,17E-07	0,038633092	0,79913081	0,955354079	-0,156223269				N_Shore	No
cg01994486	chr7	#########	#########	5,67E-07	0,038633092	0,922520343	0,826163095	0,096357248					No
cg01406381	chr19	47288262	47288263	9,28E-07	0,048433726	0,043019568	0,108315935	-0,065296367	SLC1A5;SLC	NM_00114	TSS200;Boc	N_Shelf	No
cg18442362	chr7	44677771	44677772	1,02E-06	0,048433726	0,150445244	0,256474723	-0,106029479	OGDH;OGD	NM_00100	Body;Body;Body		Yes
cg22274117	chr6	16713612	16713613	1,45E-06	0,054667686	0,057057863	0,134002171	-0,076944309	ATXN1;ATX	NM_00112	5'UTR;5'UTR		No
cg05017994	chr5	964561	964562	1,57E-06	0,054667686	0,083697235	0,170004708	-0,086307472				S_Shore	No
cg06442294	chr19	18404194	18404195	1,57E-06	0,054667686	0,905422128	0,803152883	0,102269245					Yes
cg14612335	chr3	#########	#########	1,68E-06	0,054667686	0,0690869	0,153616341	-0,084529442	SKIL;SKIL	NM_00541	TSS1500;TS	N_Shore	No
cg13092901	chr22	50965372	50965373	2,37E-06	0,054667686	0,066860939	0,121692127	-0,054831188	TYMP;SCO2	NM_00111	Body;TSS15	Island	No
cg14204586	chr1	#########	#########	2,56E-06	0,054667686	0,117385111	0,226603371	-0,109218259	ARHGEF2;A	NM_00472	Body;Body;Body		Yes
cg01372366	chr11	48041782	48041783	2,58E-06	0,054667686	0,083895567	0,179350767	-0,0954552	PTPRJ;PTPR	NM_00284	Body;Body		No
cg21494379	chr5	88275243	88275244	2,72E-06	0,054667686	0,091006343	0,201932842	-0,1109265					Yes
cg02610723	chr16	88850534	88850535	2,93E-06	0,054667686	0,049579577	0,110814234	-0,061234658	FAM38A	NM_00114	Body	Island	No
cg23072383	chr22	31031044	31031045	2,99E-06	0,054667686	0,048600504	0,108905474	-0,060304969	SLC35E4	NM_00100	TSS1500	N_Shore	No
cg21766592	chr19	47288066	47288067	3,04E-06	0,054667686	0,041789727	0,117677016	-0,075887289	SLC1A5;SLC	NM_00114	1stExon;5'U	N_Shelf	No
cg22331294	chr3	45698298	45698299	3,37E-06	0,054667686	0,10458964	0,197099644	-0,092510004	LIMD1	NM_01424	Body		No
cg17666418	chr6	#########	#########	3,45E-06	0,054667686	0,080168081	0,201668642	-0,12150056					Yes
cg02896872	chr1	#########	#########	3,57E-06	0,054667686	0,950102291	0,87600371	0,074098581	ITPKB	NM_00222	Body	N_Shelf	No
cg26954174	chr16	50730813	50730814	3,79E-06	0,054667686	0,092329351	0,206536036	-0,114206685	NOD2	NM_02216	TSS1500		Yes
cg02841912	chr6	#########	#########	3,91E-06	0,054667686	0,105531162	0,240811959	-0,135280798	SYNE1;SYNI	NM_18296	5'UTR;5'UT	N_Shore	No
cg00046336	chr2	#########	#########	4,07E-06	0,054667686	0,912123266	0,783715695	0,128407571				Island	No
cg01437204	chr1	#########	#########	4,09E-06	0,054667686	0,099074059	0,217415288	-0,11834123	PTPN7;PTPI	NM_08058	TSS1500;TSS1500		No
cg05072215	chr9	#########	#########	4,17E-06	0,054667686	0,631026813	0,758463195	-0,127436382	HSPA5	NM_00534	Body	N_Shore	No
cg03992114	chr13	#########	#########	4,26E-06	0,054667686	0,091734423	0,182973532	-0,091239109	ATP11A;AT	NM_01520	TSS1500;TS	N_Shore	Yes
cg04033022	chr16	89189383	89189384	4,31E-06	0,054667686	0,937032954	0,874814087	0,062218868	ACSF3;ACSF	NM_17491	Body;Body;	S_Shelf	No
cg00144180	chr2	#########	#########	4,54E-06	0,054667686	0,941092975	0,853193266	0,087899709	HDAC4	NM_00603	5'UTR		Yes
cg06666727	chr8	#########	#########	4,67E-06	0,054667686	0,952470753	0,87100631	0,081464443	KIFC2	NM_14575	Body	N_Shore	No
cg01657995	chr6	31804882	31804883	4,73E-06	0,054667686	0,085844038	0,169631559	-0,083787521	C6orf48;C6	NM_00104	5'UTR;5'UT	S_Shelf	No
cg05471495	chr2	#########	#########	4,78E-06	0,054667686	0,157185864	0,272243849	-0,115057985					No
cg08105590	chr16	88849598	88849599	4,88E-06	0,054667686	0,150672183	0,248621802	-0,097949619	FAM38A	NM_00114	Body	N_Shore	No
cg04742550	chr16	31366429	31366430	4,90E-06	0,054667686	0,049897876	0,123530583	-0,073632707	ITGAX	NM_00088	TSS200		No
cg13214542	chr18	33552018	33552019	4,97E-06	0,054667686	0,067502648	0,129460703	-0,061958054	C18orf21	NM_03144	TSS1500	N_Shore	No
cg20831413	chr11	72460551	72460552	4,99E-06	0,054667686	0,175750345	0,293201137	-0,117450793	ARAP1	NM_00104	5'UTR	N_Shelf	Yes
cg10524576	chr12	63322643	63322644	5,65E-06	0,054667686	0,158251362	0,263657694	-0,105406333	PPM1H	NM_02070	Body		Yes
cg04873833	chr12	#########	#########	5,68E-06	0,054667686	0,941289687	0,821391809	0,119897879	GLT1D1	NM_14466	Body		No
cg07329251	chr11	10476662	10476663	5,77E-06	0,054667686	0,173943957	0,277878636	-0,103934679	AMPD3;AM	NM_00102	TSS200;TSS	S_Shelf	No

cg02278841	chr15	38988860	38988861	5,91E-06	0,054667686	0,06425276	0,125326288	-0,061073528	C15orf53	NM_20744	1stExon		No
cg11185549	chr12	#########	#########	6,13E-06	0,054667686	0,086761859	0,1509902	-0,064228342	MAP1LC3B	NM_00108	TSS1500		No
cg00252813	chr12	6642228	6642229	6,19E-06	0,054667686	0,088753311	0,172754669	-0,084001358	PTPRJ;PTPR	NM_00284	Body;Body		Yes
cg11468953	chr11	48034953	48034954	6,19E-06	0,054667686	0,082491432	0,148616583	-0,066125151	GAPDH	NM_00204	TSS1500	N_Shore	No
cg18417061	chr14	61972660	61972661	6,20E-06	0,054667686	0,93666809	0,874514389	0,062153701	PRKCH	NM_00625	Body		No
cg06521852	chr22	38141419	38141420	6,24E-06	0,054667686	0,122611958	0,203906255	-0,081294297	TRIOBP;TRI	NM_00703	TSS1500;TS	N_Shore	No
cg19578660	chr10	3511600	3511601	6,26E-06	0,054667686	0,145860544	0,282017244	-0,1361567					Yes
cg03881294	chr2	11884333	11884334	6,46E-06	0,054667686	0,067899875	0,1314346	-0,063534725				N_Shore	Yes
cg25607249	chr19	47288039	47288040	6,49E-06	0,054667686	0,053617338	0,124881797	-0,071264459	SLC1A5;SLC	NM_00114	1stExon;5'U	N_Shelf	No
cg13405631	chr6	36259166	36259167	6,68E-06	0,054667686	0,099810371	0,175770603	-0,075960233	PNPLA1;PN	NM_17367	5'UTR;Body;5'UTR		No
cg18919209	chr11	96012038	96012039	6,73E-06	0,054667686	0,942174302	0,880451582	0,06172272	MAML2	NM_03242	Body		No
cg04727071	chr19	4061359	4061360	6,74E-06	0,054667686	0,209568519	0,345115133	-0,135546613	ZBTB7A	NM_01589	5'UTR	S_Shore	Yes
cg25194612	chr11	#########	#########	6,76E-06	0,054667686	0,909271709	0,792966197	0,116305512				Island	No
cg10503007	chr2	11796989	11796990	7,13E-06	0,055848683	0,811553267	0,642799208	0,168754059				Island	No
cg02351277	chr3	#########	#########	7,30E-06	0,055848683	0,117338926	0,216501829	-0,099162903					Yes
cg19311448	chr2	11797017	11797018	7,48E-06	0,055848683	0,809224606	0,688680308	0,120544298				Island	No
cg13443844	chr20	55904633	55904634	7,98E-06	0,055848683	0,934688225	0,884466867	0,050221358	SPO11;SPO	NM_01244	TSS200;TSS	N_Shore	No
cg09037630	chr6	31528239	31528240	8,11E-06	0,055848683	0,092613083	0,179143953	-0,08653087					Yes
cg07102001	chr16	87734816	87734817	8,15E-06	0,055848683	0,866796687	0,752193668	0,11460302	LOC100129	NR_024488	Body	N_Shore	Yes
cg04645070	chr11	34393105	34393106	8,31E-06	0,055848683	0,123188156	0,243198298	-0,120010142					No
cg27004870	chr16	88850383	88850384	8,49E-06	0,055848683	0,065133808	0,129303995	-0,064170187	FAM38A	NM_00114	Body	Island	No
cg01464985	chr2	27805622	27805623	8,64E-06	0,055848683	0,100414135	0,168112942	-0,067698806	ZNF512	NM_03243	TSS1500	N_Shore	Yes
cg09164913	chr17	78963220	78963221	8,66E-06	0,055848683	0,232952422	0,349135717	-0,116183295				N_Shelf	Yes
cg12018718	chr2	8684089	8684090	8,93E-06	0,05681955	0,086083323	0,199258677	-0,113175354					No
cg22116041	chr4	#########	#########	9,06E-06	0,056853002	0,130451066	0,223634412	-0,093183346	TMEM154	NM_15268	Body		Yes
cg15832662	chr11	63448437	63448438	9,26E-06	0,057251549	0,124099699	0,240643052	-0,116543353	RTN3;RTN3	NM_20142	TSS1500;TS	N_Shore	Yes
cg07925587	chr12	52583324	52583325	9,36E-06	0,057251549	0,061966411	0,115728666	-0,053762254	KRT80;KRT8	NM_00108	Body;Body		No
cg00737841	chr10	#########	#########	9,59E-06	0,057567332	0,918400857	0,751913942	0,166486915					No
cg13591783	chr9	75768867	75768868	9,87E-06	0,057567332	0,099446212	0,210043655	-0,110597444	ANXA1	NM_00070	5'UTR		Yes
cg07114422	chr1	2183438	2183439	9,90E-06	0,057567332	0,885546351	0,785717841	0,099828509	SKI	NM_00303	Body		No
cg03139435	chr20	54966400	54966401	1,01E-05	0,057841623	0,133568585	0,23647021	-0,102901625	AURKA;AUR	NM_19843	5'UTR;5'UT	N_Shore	No
cg22339338	chr1	#########	#########	1,03E-05	0,058294239	0,136278157	0,20410832	-0,067830163	TAGLN2	NM_00356	5'UTR	N_Shelf	Yes
cg05496814	chr5	#########	#########	1,07E-05	0,059329275	0,167258733	0,264768446	-0,097509712					Yes
cg23953820	chr6	30851050	30851051	1,08E-05	0,059329275	0,231161381	0,358393316	-0,127231935	ATXN1;ATX	NM_00112	5'UTR;5'UTR		Yes
cg24067911	chr6	16729609	16729610	1,08E-05	0,059329275	0,791039432	0,675773821	0,115265612	DDR1;DDR1	NM_00195	TSS1500;TS	N_Shore	Yes
cg13984040	chr12	#########	#########	1,12E-05	0,060651352	0,174774634	0,338402715	-0,16362808					No
cg17950348	chr7	#########	#########	1,14E-05	0,060915757	0,142935487	0,25123028	-0,108294793	DNAJB6	NM_05824	Body		Yes
cg09650907	chr17	71224982	71224983	1,16E-05	0,061287572	0,067920145	0,131873375	-0,06395323	FAM104A;F	NM_03283	Body;Body	N_Shelf	Yes
cg18147543	chr1	16276293	16276294	1,17E-05	0,061434633	0,074074872	0,170515524	-0,096440653	ZBTB17	NM_00344	5'UTR		Yes
cg20363347	chr11	69061472	69061473	1,24E-05	0,063152005	0,172042887	0,268388978	-0,096346092	MYEOV	NM_13876	TSS200		No
cg10588720	chr6	23397852	23397853	1,25E-05	0,063152005	0,146617489	0,292694046	-0,146076557	VIM	NM_00338	Body	S_Shore	Yes
cg26063719	chr10	17273186	17273187	1,25E-05	0,063152005	0,828762011	0,890291109	-0,061529099					No
cg21241410	chr2	43267815	43267816	1,27E-05	0,063152005	0,134001342	0,273863611	-0,139862269					No
cg02711665	chr8	11664728	11664729	1,29E-05	0,063152005	0,828521649	0,905004656	-0,076483007	FDFT1	NM_00446	Body	N_Shore	No
cg26253134	chr2	70751720	70751721	1,29E-05	0,063152005	0,139957438	0,245032506	-0,105075068	TGFA;TGFA	NM_00323	Body;Body		Yes

cg23367351	chr7	2106028	2106029	1,32E-05	0,063152005	0,896709448	0,810881979	0,085827469	MAD1L1;M	NM_00355	Body;Body;	N_Shore	No
cg13359998	chr1	#########	#########	1,33E-05	0,063152005	0,138341398	0,257131171	-0,118789773	GALNT2	NM_00448	Body		No
cg13921921	chr1	#########	#########	1,33E-05	0,063152005	0,087129569	0,181495614	-0,094366045	ARHGEF2;A	NM_00472	Body;Body;Body		Yes
cg05492306	chr19	45927593	45927594	1,35E-05	0,063152005	0,130904719	0,240007915	-0,109103196	ERCC1;ERC	NM_00198	TSS1500;TS	S_Shore	No
cg26369418	chr5	#########	#########	1,35E-05	0,063152005	0,114624564	0,179706696	-0,065082132	CSF2	NM_00075	TSS200		No
cg15361750	chr19	47839131	47839132	1,37E-05	0,063272344	0,141450885	0,267173471	-0,125722586	GPR77	NM_01848	TSS1500		Yes
cg14854503	chr8	19540271	19540272	1,38E-05	0,063272344	0,204291384	0,336087834	-0,13179645	CSGALNACT	NM_01837	TSS200;TSS200		No
cg05117638	chr6	#########	#########	1,41E-05	0,063272344	0,061257524	0,116519009	-0,055261485					No
cg23838005	chr17	74868604	74868605	1,41E-05	0,063272344	0,127352859	0,192928881	-0,065576022	MGAT5B;M	NM_14467	Body;TSS20	S_Shore	No
cg08698159	chr17	27294743	27294744	1,44E-05	0,063467423	0,063837237	0,137288433	-0,073451195	SEZ6;SEZ6	NM_00109	Body;Body		No
cg14293575	chr22	18635460	18635461	1,47E-05	0,063467423	0,935006084	0,864685873	0,070320211	USP18	NM_01741	5'UTR	S_Shelf	No
cg00244001	chr10	#########	#########	1,50E-05	0,063467423	0,94091499	0,868166071	0,072748919	IQSEC3;IQS	NM_00117	Body;5'UTR	Island	No
cg01239735	chr12	214224	214225	1,50E-05	0,063467423	0,082080671	0,148766451	-0,066685781	FAM53B	NM_01466	Body		Yes
cg05899984	chr12	#########	#########	1,53E-05	0,063467423	0,922332486	0,860536763	0,061795724				N_Shore	No
cg08212862	chr5	#########	#########	1,53E-05	0,063467423	0,906814771	0,77950085	0,127313921	PCDHB6	NM_01893	1stExon	N_Shore	No
cg00916640	chr12	34498371	34498372	1,55E-05	0,063467423	0,903454363	0,805880674	0,097573688				S_Shore	No
cg02711608	chr19	47287963	47287964	1,56E-05	0,063467423	0,105162348	0,186771836	-0,081609489	SLC1A5;SLC	NM_00114	1stExon;5'U	N_Shelf	No
cg27536559	chr22	36465149	36465150	1,56E-05	0,063467423	0,10187078	0,172559401	-0,070688621				S_Shelf	No
cg24185397	chr17	25659608	25659609	1,60E-05	0,063467423	0,068289422	0,129257389	-0,060967967				N_Shore	Yes
cg17412005	chr1	45806886	45806887	1,61E-05	0,063467423	0,943679707	0,882167007	0,0615127	TBCD	NM_00599	Body	S_Shelf	No
cg19755815	chr17	80873572	80873573	1,61E-05	0,063467423	0,150762062	0,255264762	-0,104502699	MUTYH;MU	NM_00104	TSS1500;TS	S_Shore	No
cg20382154	chr2	#########	#########	1,66E-05	0,064478619	0,163190358	0,255143239	-0,091952881					No
cg10152449	chr7	2444534	2444535	1,70E-05	0,064478619	0,802731757	0,608003017	0,19472874				Island	No
cg16414030	chr3	#########	#########	1,70E-05	0,064478619	0,221497733	0,342971182	-0,121473449	CHST12	NM_01864	5'UTR	S_Shore	Yes
cg04324917	chr16	87734877	87734878	1,72E-05	0,064610263	0,895741318	0,808999244	0,086742074	LOC100129	NR_024488	Body	N_Shore	Yes
cg14795163	chr1	74620779	74620780	1,73E-05	0,064610263	0,848788695	0,906550445	-0,05776175	LRRIQ3	NM_00110	Body		No
cg16755833	chr17	80866063	80866064	1,76E-05	0,065006821	0,944393439	0,892340378	0,052053061	TBCD	NM_00599	Body	S_Shelf	No
cg03669394	chr16	75685253	75685254	1,77E-05	0,065006821	0,170706185	0,260239556	-0,089533371	TERF2IP	NM_01897	Body	S_Shelf	Yes
cg25278941	chr6	#########	#########	1,83E-05	0,065477416	0,175877251	0,302957038	-0,127079788					Yes
cg26550214	chr5	34609492	34609493	1,84E-05	0,065477416	0,583420016	0,711086375	-0,127666359					Yes
cg19284277	chr17	80190053	80190054	1,88E-05	0,065477416	0,101057093	0,187859972	-0,08680288	SLC16A3;SL	NM_00104	TSS200;5'U	S_Shore	Yes
cg04973995	chr10	74057977	74057978	1,89E-05	0,065477416	0,105849932	0,202972875	-0,097122943				Island	No
cg13408655	chr9	#########	#########	1,89E-05	0,065477416	0,145488902	0,232939232	-0,08745033	COL15A1	NM_00185	Body		No
cg05897571	chrX	51148628	51148629	1,93E-05	0,065735383	0,921379722	0,777608931	0,143770791				Island	No
cg14113035	chr2	#########	#########	1,93E-05	0,065735383	0,831055547	0,905798422	-0,074742875	CXorf67	NM_20340	TSS1500	N_Shore	No
cg21545859	chr3	5068037	5068038	1,97E-05	0,065735383	0,103265344	0,193684283	-0,090418939				Island	No
cg15268456	chr8	#########	#########	1,98E-05	0,065735383	0,81195918	0,670741608	0,141217573					No
cg15095917	chr17	7482693	7482694	1,99E-05	0,065735383	0,095966382	0,188207469	-0,092241087	CD68;CD68	NM_00104	TSS200;TSS	N_Shelf	No
cg27073066	chr19	2169160	2169161	1,99E-05	0,065735383	0,131273269	0,230055701	-0,098782432	DOT1L	NM_03248	Body	S_Shelf	No
cg12510708	chr7	26193805	26193806	2,00E-05	0,065735383	0,156530432	0,265923544	-0,109393112	NFE2L3	NM_00428	Body	S_Shore	Yes
cg05305327	chr20	32156436	32156437	2,05E-05	0,065735383	0,900187256	0,794978079	0,105209178	CBFA2T2;C	NM_00103	Body;5'UTR;5'UTR		No
cg04111435	chr11	63448454	63448455	2,10E-05	0,065735383	0,109171011	0,211364475	-0,102193464	RTN3;RTN3	NM_20142	TSS1500;TS	N_Shore	Yes
cg07856975	chr6	36356161	36356162	2,11E-05	0,065735383	0,452131094	0,642012755	-0,189881662	ETV7	NM_01613	TSS1500	S_Shore	No
cg23574583	chr13	36705996	36705997	2,11E-05	0,065735383	0,922298475	0,810871502	0,111426973	DCLK1	NM_00473	TSS1500	S_Shore	No
cg23052776	chr14	71609157	71609158	2,12E-05	0,065735383	0,078315186	0,137427781	-0,059112595					Yes

cg24046474	chr9	#########	#########	2,15E-05	0,065735383	0,144888885	0,219329402	-0,074440517	RPL12;LRSA	NM_00097	Body;TSS15	N_Shore	No
cg05168229	chr13	45390049	45390050	2,16E-05	0,065735383	0,149797243	0,237621721	-0,087824478					No
cg16680873	chr7	1798868	1798869	2,16E-05	0,065735383	0,849342411	0,77027946	0,079062951					No
cg20558320	chr2	#########	#########	2,17E-05	0,065735383	0,88665087	0,812993493	0,073657376					No
cg05215994	chr13	23310203	23310204	2,20E-05	0,065735383	0,172185631	0,261919768	-0,089734137					Yes
cg15257755	chr8	22225668	22225669	2,20E-05	0,065735383	0,90172963	0,716772923	0,184956707					No
cg20979737	chr1	#########	#########	2,20E-05	0,065735383	0,124414656	0,211038183	-0,086623527	SLC39A14;S	NM_00112	5'UTR;5'UT	S_Shore	Yes
cg00224807	chr2	#########	#########	2,28E-05	0,06661797	0,951890622	0,842823771	0,109066851					No
cg05903800	chrX	48554185	48554186	2,29E-05	0,06661797	0,782079731	0,64194144	0,140138292	SUV39H1	NM_00317	TSS1500	N_Shore	No
cg09076123	chr1	#########	#########	2,29E-05	0,06661797	0,121618193	0,208823003	-0,08720481	NCF2;NCF2	NM_00112	5'UTR;1stExon;5'UTR		Yes
cg09997082	chr19	46170946	46170947	2,32E-05	0,06661797	0,319641061	0,441937947	-0,122296885	GIPR	NM_00016	TSS1500	N_Shelf	No
cg09692677	chr20	34359756	34359757	2,33E-05	0,06661797	0,058792525	0,110129776	-0,051337251	AHCYL2;AH	NM_00113	1stExon;Body;1stExon;		No
cg17827670	chr7	#########	#########	2,33E-05	0,06661797	0,086096488	0,035386211	0,050710277	PHF20	NM_01643	TSS200	Island	No
cg06928797	chr11	#########	#########	2,38E-05	0,066765639	0,090705809	0,17411428	-0,083408471	SIK3	NM_02516	Body	N_Shelf	Yes
cg12898019	chr17	74621049	74621050	2,38E-05	0,066765639	0,881710324	0,710982423	0,170727901					No
cg19317226	chr10	#########	#########	2,38E-05	0,066765639	0,187229698	0,302972456	-0,115742758	ST6GALNA(	NM_01841	3'UTR		Yes
cg04957307	chr12	92180807	92180808	2,43E-05	0,067215825	0,940930663	0,886144517	0,054786146					No
cg01445100	chr16	88103339	88103340	2,44E-05	0,067215825	0,809247866	0,676620736	0,13262713	BANP;BANF	NM_01786	Body;Body	S_Shore	Yes
cg10442729	chr2	#########	#########	2,47E-05	0,067215825	0,821461177	0,687655038	0,133806139					No
cg15865892	chr2	#########	#########	2,47E-05	0,067215825	0,224698875	0,326832156	-0,102133282				N_Shore	No
cg17223520	chr1	32739113	32739114	2,48E-05	0,067215825	0,880863471	0,777519031	0,103344441	LCK;LCK	NM_00535	5'UTR;TSS1	N_Shore	No
cg00625963	chr1	32740034	32740035	2,50E-05	0,067215825	0,932948158	0,85200228	0,080945878	LCK;LCK	NM_00535	Body;Body	N_Shore	No
cg26489179	chrX	39921142	39921143	2,51E-05	0,067215825	0,929550169	0,859163148	0,070387021	BCOR;BCOF	NM_01774	Body;Body;Body;Body		No
cg26811385	chr4	#########	#########	2,53E-05	0,06734003	0,085274544	0,153748963	-0,068474419					No
cg08469255	chr6	30851068	30851069	2,55E-05	0,067683442	0,838963818	0,736918972	0,102044846	DDR1;DDR1	NM_00195	TSS1500;TS	N_Shore	Yes
cg09333631	chr3	44802604	44802605	2,60E-05	0,06825311	0,51348704	0,315806407	0,197680633	KIF15;KIAA	NM_02024	TSS1500;Bo	N_Shore	No
cg01443020	chr17	15653096	15653097	2,67E-05	0,069140461	0,366984076	0,228574459	0,138409617				Island	No
cg02474731	chr2	#########	#########	2,68E-05	0,069140461	0,730819142	0,532967892	0,19785125	LYPD6	NM_19431	TSS1500	N_Shore	No
cg23349968	chr3	11887527	11887528	2,70E-05	0,069237899	0,135709572	0,248066446	-0,112356874	C3orf31	NM_13880	Body	N_Shore	No
cg02543462	chr2	#########	#########	2,72E-05	0,069494072	0,127793177	0,245142283	-0,117349106	IL1RN;IL1RI	NM_17384	TSS200;5'UTR;Body;Bo		No
cg23190089	chr11	2920208	2920209	2,74E-05	0,069511073	0,048570395	0,10711558	-0,058545185	SLC22A18A	NM_00710	Body;TSS15	N_Shelf	No
cg06745229	chr16	34378804	34378805	2,79E-05	0,069907428	0,871975448	0,769602808	0,10237264					No
cg01083549	chr16	89168371	89168372	2,80E-05	0,069907428	0,913603298	0,837538121	0,076065177	ACSF3;ACSI	NM_17491	Body;Body;	S_Shore	No
cg02284014	chr6	41674099	41674100	2,83E-05	0,0701613	0,041327954	0,102926393	-0,061598439	TFEB;TFEB	NM_00116	5'UTR;5'UTR		No
cg03018771	chr1	27940619	27940620	2,86E-05	0,0701613	0,770154018	0,668995278	0,10115874	FGR;FGR;F(	NM_00104	Body;Body;	N_Shore	No
cg26728422	chr16	1429015	1429016	2,87E-05	0,0701613	0,07199334	0,133008703	-0,061015363	UNKL	NM_02307	5'UTR	N_Shore	No
cg01973676	chr7	#########	#########	2,90E-05	0,0701613	0,14046693	0,241691878	-0,101224948	CUX1;CUX1	NM_18150	Body;Body;Body		No
cg02049210	chr8	48647582	48647583	2,91E-05	0,0701613	0,126495613	0,247116159	-0,120620546	KIAA1274	NM_01443	5'UTR		No
cg06285727	chr11	72524027	72524028	2,91E-05	0,0701613	0,084591409	0,150682594	-0,066091186	ATG16L2	NM_03338	TSS1500	N_Shore	Yes
cg25204955	chr10	72254313	72254314	2,91E-05	0,0701613	0,101893068	0,174960666	-0,073067598	KIAA0146	NM_00108	Body	N_Shelf	No
cg09234599	chr12	#########	#########	2,98E-05	0,071405869	0,140162289	0,232297737	-0,092135449				N_Shore	No
cg17131553	chr8	#########	#########	3,01E-05	0,071405869	0,858307773	0,94592519	-0,087617417	TRPS1	NM_01411	5'UTR	N_Shelf	No
cg02088996	chr7	41817771	41817772	3,02E-05	0,071405869	0,154082705	0,263600053	-0,109517347	LOC285954	NR_027118	Body		Yes
cg23121335	chr11	67784488	67784489	3,05E-05	0,071405869	0,120108182	0,18083939	-0,060731208	ALDH3B1;A	NM_00116	Body;Body;Body		No
cg09583957	chr20	57428315	57428316	3,06E-05	0,071405869	0,931767774	0,847742939	0,084024835	GNAS;GNAS	NM_08042	5'UTR;5'UT	Island	No

cg18458509	chr11	2920188	2920189	3,07E-05	0,071405869	0,117863469	0,190712148	-0,072848679	SLC22A18A	NM_00710	Body;TSS15	N_Shelf	Yes
cg07100542	chr1	17402079	17402080	3,08E-05	0,071405869	0,116866446	0,245850291	-0,128983845	EFHD2	NM_02432	Body	S_Shore	Yes
cg25978218	chr1	15738732	15738733	3,08E-05	0,071405869	0,080099138	0,142607382	-0,062508244	PADI2	NM_00736	Body		No
cg18396403	chr22	38615368	38615369	3,10E-05	0,071405869	0,149413644	0,246389252	-0,096975608	TMEM184B	NM_01226	3'UTR		Yes
cg04289314	chr2	#########	#########	3,19E-05	0,073259781	0,933438918	0,84973395	0,083704967				Island	No
cg08835221	chr22	38071607	38071608	3,25E-05	0,07373037	0,089713311	0,156212748	-0,066499437	LGALS1	NM_00230	TSS200	N_Shore	Yes
cg09067506	chr11	69065869	69065870	3,27E-05	0,073761357	0,080185179	0,135073823	-0,054888645					No
cg01955153	chr16	50769851	50769852	3,28E-05	0,073761357	0,092814578	0,157649412	-0,064834834					Yes
cg18935108	chr11	#########	#########	3,30E-05	0,073761357	0,6798425	0,550640509	0,129201991				Island	No
cg23757247	chr10	6354911	6354912	3,31E-05	0,073761357	0,897554157	0,954671933	-0,057117776					No
cg26347170	chr20	32441424	32441425	3,33E-05	0,073761357	0,89472155	0,814896679	0,079824871	CHMP4B	NM_17681	3'UTR		No
cg06576340	chr7	99954187	99954188	3,34E-05	0,073761357	0,117805571	0,214709455	-0,096903883	PILRB;PILRE	NM_17504	5'UTR;TSS1500;5'UTR		No
cg27336481	chr10	1401914	1401915	3,44E-05	0,074966234	0,915200745	0,865043917	0,050156828	ADARB2	NM_01870	Body	N_Shelf	No
cg15901722	chr5	#########	#########	3,47E-05	0,075322057	0,20321321	0,3034306	-0,10021739	PCDH24	NM_01767	TSS1500	S_Shelf	No
cg25140188	chrX	31087347	31087348	3,50E-05	0,075331386	0,243994758	0,485511594	-0,241516836				N_Shelf	No
cg10721220	chr8	29743491	29743492	3,53E-05	0,075331386	0,896774353	0,836164752	0,060609601	AJAP1;AJAP	NM_01883	Body;Body	N_Shore	No
cg15580684	chr1	4770711	4770712	3,53E-05	0,075331386	0,119023358	0,201186424	-0,082163066					Yes
cg27615938	chr17	16344573	16344574	3,57E-05	0,075331386	0,213103898	0,325270852	-0,112166954	NCRNA001	NR_027165	Body;Body;	S_Shore	No
cg10421247	chr12	#########	#########	3,63E-05	0,076067365	0,117098606	0,19032351	-0,073224904	CCDC64	NM_20731	Body	N_Shore	No
cg08877357	chr10	#########	#########	3,65E-05	0,076067365	0,143937039	0,269606548	-0,12566951					Yes
cg25937884	chr5	#########	#########	3,65E-05	0,076067365	0,086635459	0,143989667	-0,057354208				S_Shore	No
cg02961101	chr16	48533154	48533155	3,68E-05	0,076245447	0,168938819	0,301585303	-0,132646484				S_Shelf	Yes
cg11935248	chr6	4942483	4942484	3,71E-05	0,076245447	0,105134221	0,188362452	-0,083228231					No
cg15812586	chr7	#########	#########	3,71E-05	0,076245447	0,100173879	0,17261481	-0,072440931	CDYL;CDYL;	NM_00482	Body;Body;Body;Body		Yes
cg17737314	chr1	44114355	44114356	3,74E-05	0,07661338	0,175923664	0,307708416	-0,131784752	KDM4A	NM_01466	TSS1500	N_Shore	Yes
cg15605704	chr1	4770675	4770676	3,76E-05	0,07661338	0,909888599	0,859863268	0,050025332	AJAP1;AJAP	NM_01883	Body;Body	N_Shore	No
cg21108085	chr11	44591098	44591099	3,77E-05	0,076613927	0,112001295	0,18136869	-0,069367395	CD82;CD82	NM_00223	5'UTR;5'UT	S_Shelf	Yes
cg16005592	chr5	964653	964654	3,91E-05	0,078617896	0,886586443	0,787274091	0,099312352					No
cg18959621	chr13	23310464	23310465	3,95E-05	0,0791072	0,884166583	0,69538497	0,188781613					No
cg18564928	chr8	11790559	11790560	3,99E-05	0,079132904	0,937920041	0,836172181	0,10174786					No
cg25737313	chr19	12899556	12899557	3,99E-05	0,079132904	0,123727795	0,193957224	-0,070229429				N_Shore	No
cg25597390	chr10	5333614	5333615	4,00E-05	0,079132904	0,113788	0,237693844	-0,123905844					No
cg13709639	chr12	49526039	49526040	4,04E-05	0,079687876	0,079907301	0,145670587	-0,065763286	TUBA1B	NM_00608	TSS1500	S_Shore	Yes
cg17820878	chr1	27440463	27440464	4,08E-05	0,08007906	0,935271299	0,883460021	0,051811278	SLC9A1	NM_00304	Body		No
cg21249091	chr2	#########	#########	4,11E-05	0,08007906	0,207240277	0,317725991	-0,110485714					No
cg21232015	chr12	#########	#########	4,13E-05	0,08007906	0,058922586	0,132708386	-0,0737858	SLPI	NM_00306	TSS1500		No
cg23480341	chr20	43883705	43883706	4,13E-05	0,08007906	0,884156257	0,815903803	0,068252454	CHFR;CHFR	NM_00116	Body;Body;Body;Body;		Yes
cg23680451	chr10	3823790	3823791	4,15E-05	0,08007906	0,129719048	0,220242007	-0,090522958	KLF6;KLF6;K	NR_027653	Body;Body;	Island	Yes
cg08554257	chr16	50730737	50730738	4,20E-05	0,080855972	0,083541694	0,165169593	-0,081627899	NOD2	NM_02216	TSS1500		Yes
cg24028809	chr6	#########	#########	4,23E-05	0,081035893	0,1764186	0,291504023	-0,115085423	RGS17	NM_01241	5'UTR		Yes
cg08972190	chr7	2138995	2138996	4,28E-05	0,081567632	0,873519632	0,812297512	0,06122212	MAD1L1;M	NM_00355	Body;Body;Body		No
cg25738326	chr15	75463903	75463904	4,31E-05	0,081567682	0,106321764	0,161874245	-0,055552481					No
cg17971578	chr1	36852463	36852464	4,38E-05	0,082610013	0,178591351	0,258055209	-0,079463858	STK40	NM_03201	TSS1500	S_Shore	No
cg26963632	chr16	85558148	85558149	4,45E-05	0,083286544	0,144313098	0,245261884	-0,100948786					Yes
cg22584802	chr7	#########	#########	4,47E-05	0,083286544	0,086729316	0,13813655	-0,051407234	AHCYL2;AH	NM_00113	Body;TSS200;Body;TSS2		Yes

cg19858214	chr7	#########	#########	4,63E-05	0,085518147	0,136229632	0,241671606	-0,105441974					Yes
cg08129092	chr1	#########	#########	4,74E-05	0,086317746	0,125315556	0,229119782	-0,103804226	INTS3	NM_02301	3'UTR	N_Shore	Yes
cg03183540	chr8	#########	#########	4,76E-05	0,086317746	0,276546624	0,411107078	-0,134560454	TP53BP2;TI	NM_00103	Body;5'UTR		No
cg03992323	chr1	#########	#########	4,76E-05	0,086317746	0,076334234	0,152691845	-0,07635761					No
cg00359365	chr1	26880546	26880547	4,78E-05	0,086317746	0,139308043	0,243239359	-0,103931316	MIR1976;R	NR_03174	TSS1500;Body;Body		No
cg07220903	chr3	46973283	46973284	4,78E-05	0,086317746	0,144749553	0,27051323	-0,125763677	CCDC12	NM_14471	Body		Yes
cg23479730	chr14	99681757	99681758	4,80E-05	0,086493087	0,86719503	0,785861555	0,081333475	BCL11B;BCI	NM_02289	Body;Body		No
cg26376241	chr2	65594021	65594022	4,85E-05	0,086902736	0,193196449	0,348979103	-0,155782655	SPRED2;SPI	NM_00112	TSS200;Body		Yes
cg07052041	chr10	#########	#########	4,90E-05	0,087328914	0,063371951	0,120008558	-0,056636606				S_Shore	No
cg02701024	chr7	#########	#########	4,91E-05	0,087328914	0,912160504	0,803694443	0,108466061	PTN	NM_00282	5'UTR		No
cg23792308	chr17	14104658	14104659	5,01E-05	0,088786221	0,061765245	0,120461082	-0,058695837	COX10	NM_00130	Body		No
cg16037711	chr1	59280840	59280841	5,06E-05	0,089321376	0,297327258	0,421024867	-0,123697609				N_Shore	No
cg08783514	chr15	63772233	63772234	5,08E-05	0,089359735	0,805349896	0,66982643	0,135523466					No
cg20292653	chr2	5847374	5847375	5,10E-05	0,089406221	0,95296789	0,883863182	0,069104708				Island	No
cg16272554	chr3	15541262	15541263	5,15E-05	0,089586166	0,795595675	0,879458922	-0,083863246	COLQ;COLC	NM_08053	Body;Body;TSS1500		No
cg18900669	chr17	7482456	7482457	5,18E-05	0,089764708	0,111703754	0,185285009	-0,073581255	CD68;CD68	NM_00104	TSS1500;TS	N_Shelf	No
cg08639389	chr4	26995889	26995890	5,19E-05	0,089764708	0,952688793	0,896391925	0,056296868	STIM2;STIN	NM_00116	Body;Body;Body		No
cg08713711	chr5	1444333	1444334	5,22E-05	0,089971837	0,356276975	0,255404032	0,100872943	SLC6A3	NM_00104	5'UTR	Island	No
cg23752828	chr12	57941308	57941309	5,28E-05	0,090222186	0,081630042	0,14031132	-0,058681278	DCTN2	NM_00640	TSS1500	S_Shore	Yes
cg08836542	chr2	28618831	28618832	5,32E-05	0,090656543	0,213743881	0,326114682	-0,112370802	FOSL2	NM_00525	Body	S_Shelf	No
cg05516537	chr13	#########	#########	5,41E-05	0,091230562	0,123410285	0,21650035	-0,093090065	TFDP1;TFDI	NM_00711	5'UTR;Body		Yes
cg25623459	chr11	1861344	1861345	5,50E-05	0,091653715	0,11973232	0,180605531	-0,060873211	TNNI2;TNN	NM_00114	Body;Body;TSS200		No
cg27102141	chr10	#########	#########	5,50E-05	0,091653715	0,878234752	0,702771446	0,175463307				Island	No
cg02453013	chr16	1297490	1297491	5,51E-05	0,091653715	0,409040662	0,589479311	-0,180438649					No
cg13533061	chr17	74712429	74712430	5,58E-05	0,091784054	0,09590213	0,174149781	-0,078247652	AHCYL2;AH	NM_00113	Body;Body;Body;Body		No
cg15664152	chr7	#########	#########	5,58E-05	0,091784054	0,210686185	0,320293846	-0,109607661	JMJD6	NM_00108	3'UTR		Yes
cg19568845	chr11	66194067	66194068	5,63E-05	0,091784054	0,859855383	0,719938301	0,139917082	NPAS4	NM_17886	3'UTR		No
cg02519286	chr12	6642354	6642355	5,64E-05	0,091784054	0,106380934	0,165018444	-0,058637511	GAPDH	NM_00204	TSS1500	N_Shore	No
cg22810489	chr19	45927584	45927585	5,66E-05	0,091784054	0,122899668	0,222094973	-0,099195306	ERCC1;ERC	NM_00198	TSS1500;TS	S_Shore	No
cg13273540	chr3	#########	#########	5,73E-05	0,092072504	0,14333761	0,209864486	-0,066526875	TBL1XR1	NM_02466	5'UTR		Yes
cg02010481	chr7	28218524	28218525	5,74E-05	0,092072504	0,105310798	0,202065227	-0,096754429	JAZF1	NM_17506	Body	N_Shore	Yes
cg26099158	chr12	89646051	89646052	5,84E-05	0,092898051	0,625603331	0,743224124	-0,117620793					Yes
cg05293820	chr3	#########	#########	5,95E-05	0,093900303	0,101919269	0,045738965	0,056180304	STXBP5L	NM_01498	5'UTR	Island	No
cg20155035	chr1	#########	#########	6,01E-05	0,093900303	0,131088144	0,198728791	-0,067640647					No
cg21268578	chr22	38029478	38029479	6,06E-05	0,093900303	0,056486329	0,129229188	-0,072742859	GGA1;GGA	NM_00100	3'UTR;3'UTR		No
cg09138671	chr1	20125846	20125847	6,07E-05	0,093900303	0,138096672	0,215612797	-0,077516125	TMCO4	NM_18171	5'UTR	N_Shore	No
cg04583904	chr16	33627536	33627537	6,08E-05	0,093900303	0,861695418	0,775270194	0,086425224					No
cg07985366	chr6	#########	#########	6,12E-05	0,094107726	0,858843271	0,735138882	0,123704389				Island	No
cg15473092	chr2	#########	#########	6,15E-05	0,094239523	0,946008949	0,830531046	0,115477903				S_Shore	No
cg09516523	chr1	#########	#########	6,16E-05	0,094239523	0,925473172	0,85454206	0,070931112	CHI3L2;CHI	NM_00102	TSS1500;TSS1500		No
cg02656560	chr17	19967599	19967600	6,20E-05	0,094507395	0,163078738	0,262476666	-0,099397928					Yes
cg09555124	chr6	#########	#########	6,26E-05	0,094857091	0,079831276	0,139451322	-0,059620046	IL17RE;IL17	NM_15348	Body;Body;	N_Shelf	No
cg16324121	chr3	9954273	9954274	6,26E-05	0,094857091	0,885523268	0,786784397	0,09873887	IGF2R	NM_00087	Body		No
cg13615963	chr6	#########	#########	6,31E-05	0,095181994	0,084038857	0,144223604	-0,060184747	CCR6;CCR6	NM_03140	5'UTR;5'UTR;1stExon		No
cg12799314	chr10	1405192	1405193	6,32E-05	0,095181994	0,917034024	0,815078685	0,101955339	ADARB2	NM_01870	Body	Island	No

cg21699330	chr7	26193032	26193033	6,37E-05	0,095343156	0,088533394	0,186614877	-0,098081484	NFE2L3	NM_00428	Body	S_Shore	Yes
cg07145988	chr1	8692312	8692313	6,40E-05	0,095343156	0,879685911	0,802270108	0,077415803	RERE;RERE	NM_01210	Body;Body		No
cg26581503	chr22	42304579	42304580	6,47E-05	0,095850872	0,142289468	0,227839114	-0,085549646				N_Shore	Yes
cg24445388	chr1	2084391	2084392	6,61E-05	0,097372545	0,947005622	0,87897362	0,068032003	PRKCZ;PRK(	NM_00103	Body;Body;	S_Shore	No
cg26621897	chrX	#########	#########	6,69E-05	0,097402707	0,44644131	0,649300653	-0,202859343	TMSB15A	NM_02199	TSS1500	S_Shore	No
cg24721964	chr3	#########	#########	6,72E-05	0,097402707	0,156174799	0,259512871	-0,103338073	CLRN1;CLRI	NM_05299	TSS1500;Body		Yes
cg26542792	chr12	52603567	52603568	6,74E-05	0,097496361	0,171157224	0,258014863	-0,086857639	LOC283404	NR_027358	TSS1500		No
cg07280807	chr14	70317238	70317239	6,88E-05	0,098469084	0,138979639	0,22481109	-0,085831451					Yes
cg07644446	chr3	64034133	64034134	6,88E-05	0,098469084	0,883048075	0,718343764	0,164704311				Island	No
cg19160130	chr11	82681574	82681575	6,89E-05	0,098469084	0,928827496	0,849248126	0,07957937					No
cg25708790	chr12	#########	#########	6,91E-05	0,098469084	0,877580362	0,808146778	0,069433584	EP400	NM_01540	Body		No
cg25619440	chr2	#########	#########	7,00E-05	0,099009117	0,893752232	0,709168903	0,184583328				N_Shore	No
cg02244175	chr5	95178739	95178740	7,04E-05	0,099009117	0,172965556	0,284946941	-0,111981386					No
cg14622879	chr6	12234000	12234001	7,07E-05	0,099009117	0,100563507	0,183309827	-0,082746321					Yes
cg05949181	chr22	39154591	39154592	7,19E-05	0,099009117	0,897048752	0,794171226	0,102877526				S_Shelf	No
cg17218026	chr1	#########	#########	7,20E-05	0,099009117	0,377269466	0,19246593	0,184803536	ADAR;ADAI	NM_00102	5'UTR;TSS1	S_Shore	No
cg02590572	chr1	#########	#########	7,22E-05	0,099009117	0,947665059	0,884945979	0,06271908	CHI3L2;CHI	NM_00102	TSS1500;TSS1500		No
cg13048962	chr15	66790409	66790410	7,29E-05	0,099486502	0,142126166	0,2378312	-0,095705034	SNAPC5	NM_00604	TSS1500	S_Shore	No
cg16223079	chr15	75339540	75339541	7,35E-05	0,099582562	0,098040537	0,170620372	-0,072579835	PPCDC	NM_02182	Body		No
cg03552317	chr10	#########	#########	7,45E-05	0,099868449	0,86069042	0,93241888	-0,07172846				S_Shelf	No
cg05509777	chr11	2322517	2322518	7,46E-05	0,099868449	0,065996186	0,118953116	-0,05295693	C11orf21;T	NR_024621	Body;TSS1500;Body		No
cg07056138	chr8	#########	#########	7,47E-05	0,099868449	0,117890978	0,185289558	-0,067398581	NCALD;NCA	NM_00104	5'UTR;5'UTR;5'UTR;5'U		No
cg24704287	chr19	13951481	13951482	7,48E-05	0,099868449	0,239025795	0,384108524	-0,145082729				N_Shore	Yes
cg01616876	chr12	#########	#########	7,60E-05	0,100757578	0,155676478	0,266450885	-0,110774407	RASAL1	NM_00465	Body	S_Shelf	No
cg08467103	chr2	65593933	65593934	7,64E-05	0,100987528	0,313526467	0,476885267	-0,1633588	SPRED2;SPI	NM_00112	TSS200;Body		No
cg00571483	chr1	59046173	59046174	7,68E-05	0,10116181	0,130551787	0,209422479	-0,078870691				S_Shelf	Yes
cg04342904	chrX	46852029	46852030	7,71E-05	0,101187112	0,869314578	0,921494351	-0,052179773	PHF16;PHF	NM_00107	Body;Body		No
cg11264878	chr10	47004362	47004363	7,72E-05	0,101187112	0,183757835	0,256724999	-0,072967164				N_Shelf	No
cg06021088	chr2	#########	#########	7,82E-05	0,101737685	0,135425451	0,23919179	-0,103766339	BIN1;BIN1;	NM_13934	Body;Body;Body;Body;		Yes
cg17520654	chr12	13409473	13409474	7,90E-05	0,101737685	0,173115981	0,264528243	-0,091412262					No
cg25028542	chr7	36429119	36429120	7,95E-05	0,101737685	0,151874527	0,245420131	-0,093545603	ANLN;KIAA	NM_01868	TSS1500;Bo	N_Shore	Yes
cg12831034	chr20	57582971	57582972	7,97E-05	0,101737685	0,094511913	0,155063684	-0,06055177	CTSZ	NM_00133	TSS1500	S_Shore	No
cg19283806	chr18	66389419	66389420	7,98E-05	0,101737685	0,198388345	0,319832753	-0,121444409	CCDC102B	NM_00109	5'UTR		Yes
cg15108641	chr10	99263320	99263321	7,99E-05	0,101737685	0,28205994	0,399569747	-0,117509807	UBTD1	NM_02495	Body	S_Shelf	Yes
cg01404750	chr13	#########	#########	8,02E-05	0,101737685	0,915042956	0,84151874	0,073524215	ATP11A;AT	NM_01520	Body;Body	N_Shore	No
cg01919999	chr5	#########	#########	8,04E-05	0,101737685	0,161994602	0,251052599	-0,089057998	GNB2L1	NM_00609	Body	N_Shore	No
cg18506744	chr19	1102627	1102628	8,06E-05	0,101737685	0,103552098	0,173762072	-0,070209974	GPX4;GPX4	NM_00208	TSS1500;TS	N_Shore	No
cg25104727	chr1	#########	#########	8,07E-05	0,101737685	0,818598421	0,619575576	0,199022845	ASTN1;AST	NM_20710	Body;Body		No
cg19405484	chr8	29513210	29513211	8,08E-05	0,101737685	0,111897267	0,196375998	-0,084478731					No
cg25467652	chr1	11795975	11795976	8,16E-05	0,102399563	0,150488375	0,240711768	-0,090223393	AGTRAP;AG	NM_00104	TSS200;TSS	Island	Yes
cg01816191	chr3	#########	#########	8,26E-05	0,103185694	0,834723022	0,898238807	-0,063515785	FXR1;FXR1;	NM_00508	TSS1500;TS	N_Shore	No
cg21990700	chr12	7260776	7260777	8,34E-05	0,103928731	0,151281965	0,236161973	-0,084880007	LOC283314	NR_026947	TSS200;Body		Yes
cg25817701	chr7	2140142	2140143	8,41E-05	0,104195659	0,76674658	0,673288306	0,093458274	MAD1L1;M	NM_00355	Body;Body;Body		Yes
cg03318904	chr22	39801522	39801523	8,47E-05	0,104729636	0,81663114	0,739549634	0,077081506	MAP3K7IP1	NM_00611	Body;Body		No
cg15058210	chr2	#########	#########	8,61E-05	0,105284006	0,116475952	0,218707884	-0,102231932	HDAC4	NM_00603	Body	Island	Yes

cg05568549	chr6	41907198	41907199	8,63E-05	0,105284006	0,162427966	0,259624587	-0,097196621	CCND3;CCN	NM_00113	Body;Body;	N_Shore	No
cg00686132	chr6	#########	#########	8,69E-05	0,105284006	0,340289574	0,468920973	-0,128631399	MLLT4;MLL	NM_00104	Body;Body;Body		Yes
cg08880817	chr6	7909641	7909642	8,72E-05	0,105284006	0,134281368	0,210873403	-0,076592035	TXNDC5;TX	NM_03081	Body;5'UTR	N_Shore	No
cg06119477	chr20	418893	418894	8,73E-05	0,105284006	0,075389955	0,135160854	-0,059770899	TBC1D20	NM_14462	3'UTR		No
cg02626929	chr16	3011660	3011661	8,76E-05	0,105284006	0,13422332	0,191816167	-0,057592847				N_Shore	No
cg23324787	chr11	36619693	36619694	8,76E-05	0,105284006	0,950027706	0,880292656	0,06973505	C11orf74;R	NM_13878	5'UTR;1stExon;5'UTR		No
cg20225915	chr11	805725	805726	8,81E-05	0,105550204	0,082982717	0,14643312	-0,063450404	LRDD;LRDD	NM_01849	TSS1500;TS	S_Shore	Yes
cg08274633	chr15	38988533	38988534	8,87E-05	0,105868636	0,106841125	0,163733738	-0,056892613	C15orf53	NM_20744	TSS1500		No
cg13439241	chr11	63273507	63273508	8,88E-05	0,105868636	0,067845429	0,120557833	-0,052712404	LGALS12;LG	NM_03310	TSS200;TSS200;TSS200		No
cg18369972	chr17	9940120	9940121	8,90E-05	0,105868636	0,186627472	0,294496197	-0,107868725	GAS7;GAS7	NM_20143	Body;TSS200		Yes
cg26856257	chr1	32805350	32805351	8,94E-05	0,106135143	0,10223157	0,174258574	-0,072027004				S_Shelf	Yes
cg24769348	chr10	74692574	74692575	8,98E-05	0,106356856	0,178968944	0,28063527	-0,101666325	OIT3	NM_15263	3'UTR		No
cg08548498	chr20	43883545	43883546	9,08E-05	0,107108227	0,124138912	0,235754523	-0,111615611	SLPI	NM_00306	TSS1500		Yes
cg20475486	chr14	70317074	70317075	9,09E-05	0,107108227	0,872561543	0,734432031	0,138129512				Island	No
cg09837977	chr7	#########	#########	9,14E-05	0,107182838	0,931395177	0,874962262	0,056432915	LRRN3;LRR	NM_00109	5'UTR;1stExon;Body;5'U		Yes
cg24964103	chr6	#########	#########	9,14E-05	0,107182838	0,314356824	0,452594869	-0,138238046	VNN1	NM_00466	TSS200		Yes
cg12966875	chr20	43883746	43883747	9,17E-05	0,107257922	0,093945175	0,173403818	-0,079458643	SLPI	NM_00306	TSS1500		No
cg01239367	chr10	1815497	1815498	9,23E-05	0,107670328	0,910271872	0,856207046	0,054064826					No
cg26700469	chr16	79155427	79155428	9,28E-05	0,107967566	0,895879716	0,947449698	-0,051569982	WWOX	NM_01637	Body		No
cg06728055	chr3	#########	#########	9,31E-05	0,108040283	0,296570355	0,383327048	-0,086756693	WWTR1;W	NM_01547	Body;Body;Body		Yes
cg16125214	chr1	9751352	9751353	9,37E-05	0,108201835	0,918382728	0,844486374	0,073896354	PIK3CD	NM_00502	5'UTR	S_Shore	No
cg22101463	chr7	56242181	56242182	9,44E-05	0,10875479	0,821301359	0,582231169	0,239070191				N_Shore	No
cg13200854	chr4	90836547	90836548	9,52E-05	0,109219564	0,863883617	0,917637041	-0,053753424	MMRN1	NM_00735	Body		No
cg05480110	chr3	#########	#########	9,67E-05	0,10960497	0,925176191	0,855442143	0,069734048	MUC4;MUC	NM_01840	Body;Body;Body		No
cg16511445	chr1	#########	#########	9,67E-05	0,10960497	0,161167951	0,262007717	-0,100839766	SEC22B	NM_00489	3'UTR		No
cg02641539	chr8	#########	#########	9,83E-05	0,110125172	0,14012818	0,220787492	-0,080659312	TM7SF4	NM_03078	TSS1500		No
cg13790576	chr9	#########	#########	9,84E-05	0,110125172	0,393637868	0,54149555	-0,147857682	LCN6	NM_19894	Body	Island	Yes
cg22447508	chr20	13975439	13975440	9,89E-05	0,110125172	0,957331403	0,895817386	0,061514018	MACROD2	NM_08067	TSS1500	N_Shore	No
cg07971089	chr8	#########	#########	9,90E-05	0,110125172	0,046003443	0,106453058	-0,060449615				N_Shelf	No
cg08364093	chr2	28114163	28114164	9,92E-05	0,110125172	0,07637737	0,129373218	-0,052995848	BRE;RBKS;B	NM_19919	5'UTR;TSS1	S_Shore	Yes
cg23344321	chr17	28707212	28707213	9,96E-05	0,110125172	0,073651229	0,12688747	-0,053236241	CPD	NM_00130	Body	S_Shore	No
cg25213720	chr5	#########	#########	9,96E-05	0,110125172	0,222486361	0,334141303	-0,111654942	MXD3;MXD	NM_03130	3'UTR;Body	N_Shore	Yes
cg02324006	chr19	1080034	1080035	9,97E-05	0,110125172	0,891427974	0,823223245	0,068204729	HMHA1	NM_01229	Body	Island	No
cg09297514	chr12	#########	#########	9,98E-05	0,110125172	0,860234734	0,923818949	-0,063584216	SAPS2	NM_01467	Body	N_Shelf	No
cg18040409	chr22	50865814	50865815	9,98E-05	0,110125172	0,195485454	0,101435797	0,094049657	CAMKK2;CA	NM_00654	Body;3'UTR;3'UTR;Body		No
cg18076651	chr12	53625605	53625606	9,99E-05	0,110125172	0,149758341	0,215049854	-0,065291513	RARG	NM_00096	5'UTR	N_Shore	No
cg24037976	chr6	#########	#########	0,00010056	0,110125172	0,929484366	0,869700397	0,059783969				Island	No
cg09834049	chr14	23525719	23525720	0,000100911	0,110125172	0,112012981	0,162256059	-0,050243078	CDH24;CDH	NM_02247	5'UTR;5'UT	N_Shore	No
cg10973762	chr15	48625931	48625932	0,000100926	0,110125172	0,326223538	0,425728365	-0,099504827	DUT;DUT;D	NM_00102	Body;Body;	S_Shore	No
cg22700686	chr1	#########	#########	0,000101112	0,110125172	0,094827277	0,153327508	-0,05850023	S100A2	NM_00597	TSS1500		No
cg07837085	chr1	#########	#########	0,000103096	0,111589749	0,186731273	0,315573777	-0,128842504	SLAMF7	NM_02118	TSS200		Yes
cg19102955	chr20	5928063	5928064	0,000103334	0,111589749	0,230263931	0,421504118	-0,191240187	TRMT6	NM_01593	Body	N_Shelf	No
cg22129276	chr2	#########	#########	0,000104123	0,111589749	0,157362157	0,263805006	-0,106442849	DHRS9;DHF	NM_00577	5'UTR;5'UTR		No
cg09988805	chr2	43278551	43278552	0,000104191	0,111589749	0,767018523	0,629926343	0,137092181					Yes
cg20719675	chr2	#########	#########	0,000104436	0,111589749	0,79762756	0,900943675	-0,103316115	CHRNA1;CH	NM_00103	Body;Body		No

cg04281019	chr16	46461208	46461209	0,000104562	0,111589749	0,584775229	0,413968145	0,170807084				N_Shore	No
cg00034336	chr2	8684125	8684126	0,00010621	0,112586837	0,134695728	0,26239511	-0,127699383					No
cg08168737	chr11	#########	#########	0,000106356	0,112586837	0,920438942	0,836054353	0,084384589				Island	No
cg27225130	chr1	#########	#########	0,00010644	0,112586837	0,79190749	0,713575231	0,07833226	PLXNA2	NM_02517	Body		No
cg15774510	chr10	90749966	90749967	0,000107376	0,113036658	0,038829836	0,096374224	-0,057544388	FAS;FAS;FA	NR_028033	TSS1500;TS	N_Shore	No
cg08610773	chr2	#########	#########	0,000107516	0,113036658	0,829103187	0,888669412	-0,059566224					No
cg02057796	chr17	74530986	74530987	0,000107576	0,113036658	0,919902676	0,865036063	0,054866613	CYGB	NM_13426	Body	N_Shelf	No
cg19040077	chr2	#########	#########	0,000107874	0,11306767	0,201091873	0,306661936	-0,105570063	D2HGDH	NM_15278	Body		Yes
cg07367601	chr1	#########	#########	0,00010808	0,11306767	0,149891702	0,231037101	-0,081145398					Yes
cg07195577	chr17	27052828	27052829	0,000109021	0,113408135	0,051052547	0,10369874	-0,052646193	TLCD1;TLCD	NM_00116	Body;Body	N_Shore	No
cg05394456	chr16	129229	129230	0,000109119	0,113408135	0,091445022	0,174628205	-0,083183183	MPG;MPG;	NM_00101	5'UTR;Body	S_Shore	No
cg20486407	chr2	7037101	7037102	0,000110637	0,114066961	0,849673676	0,937010659	-0,087336983	RSAD2	NM_08065	3'UTR		No
cg22056595	chr7	#########	#########	0,000110764	0,114066961	0,490357188	0,349696075	0,140661113	PTPRN2;PT	NM_00284	Body;Body;Body		No
cg14959425	chr7	20447706	20447707	0,000111114	0,114066961	0,742445721	0,812798213	-0,070352493	ITGB8	NM_00221	Body		No
cg26768584	chr3	18480242	18480243	0,000111566	0,114066961	0,796293597	0,676404366	0,119889231	SATB1;SATE	NM_00113	5'UTR;1stExon		No
cg08696165	chr2	45172168	45172169	0,000112021	0,114066961	0,170520121	0,116056051	0,054464069	SIX3	NM_00541	3'UTR	S_Shore	No
cg12666727	chr1	42128486	42128487	0,000112812	0,114066961	0,115577339	0,237301148	-0,121723809	HIVEP3;HIV	NM_02450	5'UTR;5'UT	Island	No
cg20367304	chr4	#########	#########	0,000112861	0,114066961	0,844373278	0,741513314	0,102859965	KIAA0922;K	NM_01519	Body;Body	S_Shelf	No
cg26744682	chr19	50249583	50249584	0,000114281	0,114609492	0,639268514	0,42050308	0,218765433	TSKS	NM_02173	Body	Island	No
cg10413352	chr7	31375319	31375320	0,000114549	0,114609492	0,874085824	0,736640925	0,137444899				N_Shore	No
cg16298481	chr1	89989717	89989718	0,000114696	0,114609492	0,113861313	0,185954004	-0,072092692	LRRC8B	NM_00113	TSS1500	N_Shore	No
cg06817737	chr12	966395	966396	0,000114839	0,114609492	0,954502971	0,902647222	0,051855749	WNK1	NM_01897	Body		No
cg03850957	chr20	16713358	16713359	0,000115374	0,114889108	0,220752363	0,328408056	-0,107655693	SNRPB2;SN	NM_19822	Body;Body	S_Shelf	Yes
cg24015889	chr17	2951718	2951719	0,000115601	0,114889108	0,749000638	0,616432025	0,132568613				N_Shore	No
cg25769469	chr5	71643841	71643842	0,000116484	0,115284309	0,215582512	0,357066117	-0,141483605	PTCD2	NM_02475	Body		Yes
cg11095743	chr10	49815242	49815243	0,000116724	0,115284309	0,187662978	0,280689816	-0,093026838				S_Shore	No
cg22540135	chr19	51898904	51898905	0,000117124	0,11531149	0,064503996	0,129390701	-0,064886705				S_Shore	No
cg27370471	chr15	#########	#########	0,000117461	0,11531149	0,221861089	0,430471114	-0,208610024	PCSK6;PCSK	NM_00257	Body;Body;Body;Body;		No
cg10310700	chr17	80872460	80872461	0,000119789	0,116371037	0,869799389	0,787355582	0,082443807	TBCD	NM_00599	Body	S_Shore	No
cg03257930	chr9	#########	#########	0,000120263	0,116371037	0,131570619	0,206794798	-0,075224179				N_Shore	Yes
cg03252770	chr4	#########	#########	0,000121811	0,116691238	0,159741034	0,263615408	-0,103874374					No
cg06679494	chr17	6921295	6921296	0,000121817	0,116691238	0,078041505	0,144002317	-0,065960812	MIR497;MI	NR_030178	Body;TSS15	S_Shelf	No
cg09880331	chr5	95902142	95902143	0,000123037	0,117623159	0,565648222	0,687915953	-0,122267731					No
cg03548415	chr11	#########	#########	0,000123673	0,117921113	0,173160261	0,261939096	-0,088778834					Yes
cg13761818	chr10	#########	#########	0,000123843	0,117921113	0,888506598	0,943381488	-0,054874891					No
cg08189186	chr19	38793546	38793547	0,000124093	0,117924255	0,47177392	0,612875901	-0,141101981	C19orf33	NM_03352	TSS1500		No
cg06980531	chr5	#########	#########	0,000125622	0,118712523	0,823778654	0,713494285	0,11028437	FBLL1	NR_024356	Body	Island	No
cg07229767	chr4	3372206	3372207	0,00012587	0,118712523	0,901686963	0,825016318	0,076670645	RGS12;RGS	NM_19822	Body;Body;	N_Shelf	No
cg17167303	chr17	77375711	77375712	0,000126118	0,118712523	0,89358339	0,814760243	0,078823147	HRNBP3	NM_00108	5'UTR		No
cg25320328	chr1	92953036	92953037	0,000126167	0,118712523	0,22914184	0,344289208	-0,115147368	GFI1;GFI1	NM_00112	TSS1500;TS	S_Shore	Yes
cg01974478	chr7	4779311	4779312	0,000126883	0,118987491	0,927369753	0,867180201	0,060189552	FOXK1	NM_00103	Body		No
cg15945235	chr10	90611603	90611604	0,000127018	0,118987491	0,101945873	0,179779932	-0,077834059	ANKRD22;A	NM_14459	1stExon;5'UTR		No
cg26101277	chr17	32690412	32690413	0,000127863	0,118987491	0,096851096	0,162732689	-0,065881593	CCL1	NM_00298	TSS200		Yes
cg05514299	chr17	74868670	74868671	0,000127873	0,118987491	0,132408719	0,208542043	-0,076133324	MGAT5B;M	NM_14467	Body;TSS20	S_Shelf	Yes
cg04027328	chr1	11372138	11372139	0,000128459	0,118987491	0,172161492	0,227023863	-0,054862371					No

cg18159802	chr11	#########	#########	0,000128606	0,118987491	0,131395728	0,194263134	-0,062867406					No
cg19413066	chr17	76965346	76965347	0,000128676	0,118987491	0,348833665	0,432531924	-0,083698259					Yes
cg13677800	chr10	#########	#########	0,000129701	0,118987491	0,929264906	0,863829165	0,065435741	GPR26	NM_15344	Body	Island	No
cg00950718	chr1	#########	#########	0,000129799	0,118987491	0,097328095	0,18740609	-0,090077995	CCDC19	NM_01233	Body		No
cg26170244	chr15	65176352	65176353	0,00012991	0,118987491	0,141017459	0,235030115	-0,094012656					Yes
cg26269881	chr3	5023310	5023311	0,000130216	0,118987491	0,160706943	0,268771591	-0,108064649	BHLHE40	NM_00367	Body	N_Shore	Yes
cg20234640	chr19	7767089	7767090	0,000130248	0,118987491	0,876663145	0,811224967	0,065438178	FCER2	NM_00200	TSS200		No
cg02463988	chr7	22817223	22817224	0,00013045	0,118987491	0,787891654	0,873185685	-0,085294031					No
cg16881676	chr12	49659619	49659620	0,000131172	0,119055496	0,107775695	0,169924273	-0,062148578	TUBA1C	NM_03270	Body	S_Shore	Yes
cg04583430	chr5	#########	#########	0,000131314	0,119055496	0,866637397	0,789963209	0,076674189					No
cg11134430	chr3	#########	#########	0,000131555	0,119055496	0,199073525	0,303505109	-0,104431585				Island	No
cg01644741	chr20	47896888	47896889	0,000132173	0,119055496	0,0959243	0,148796796	-0,052872496	MIR1259;SI	NR_03166(	Body;Body;	S_Shore	Yes
cg06647068	chr12	#########	#########	0,00013223	0,119055496	0,237524615	0,343805673	-0,106281058	CHST11	NM_01841	Body	S_Shore	Yes
cg03520542	chr6	#########	#########	0,000133629	0,119134218	0,445571607	0,349987595	0,095584011				N_Shore	No
cg25729350	chr15	58624534	58624535	0,000134341	0,119134218	0,128880887	0,205630025	-0,076749138					No
cg18054654	chr5	70246896	70246897	0,000134801	0,119134218	0,88555885	0,828994059	0,056564791	SMN2;SMN	NM_01741	Body;Body;Body;Body;(		No
cg10133462	chr4	1305112	1305113	0,000135619	0,119134218	0,823292366	0,726726129	0,096566237	MAEA;MAE	NM_00588	Body;Body	Island	No
cg11088672	chr4	#########	#########	0,000135808	0,119134218	0,24595874	0,354503139	-0,108544399					Yes
cg05964640	chr16	84390897	84390898	0,000135938	0,119134218	0,048768965	0,100941553	-0,052172588					No
cg10160612	chr16	30751898	30751899	0,000136084	0,119134218	0,251965059	0,333749132	-0,081784073					No
cg18293633	chr16	88453823	88453824	0,000136385	0,119134218	0,883382118	0,79829863	0,085083488				Island	No
cg21741284	chr11	2908035	2908036	0,000136892	0,119134218	0,420860608	0,527681934	-0,106821325	CDKN1C;CD	NM_00007	TSS1500;TS	S_Shore	No
cg10325497	chr1	32739048	32739049	0,000137105	0,119134218	0,82669595	0,710007051	0,116688899	LCK;LCK	NM_00535	5'UTR;TSS1	N_Shore	No
cg11011533	chr17	3674649	3674650	0,000137712	0,119134218	0,873985383	0,793501358	0,080484025	ITGAE	NM_00220	Body		Yes
cg00687714	chr16	21272887	21272888	0,000139232	0,119134218	0,62416776	0,750403497	-0,126235737	CRYM;CRYN	NM_00101	Body;Body		No
cg09884146	chr2	65593908	65593909	0,000139313	0,119134218	0,25746676	0,395765381	-0,138298622	SPRED2;SPI	NM_00112	5'UTR;1stExon;Body		Yes
cg06235575	chr20	62795838	62795839	0,000141344	0,119134218	0,857380106	0,727964292	0,129415814	MYT1;MYT:	NM_00453	5'UTR;1stExon		No
cg20816447	chr4	15480781	15480782	0,000141399	0,119134218	0,181857719	0,28392396	-0,10206624	CC2D2A;CC	NM_00108	Body;Body;Body		Yes
cg09735627	chr7	#########	#########	0,000141541	0,119134218	0,884225731	0,787085759	0,097139972					No
cg00450651	chr5	#########	#########	0,000141617	0,119134218	0,15475123	0,269007023	-0,114255794				N_Shore	Yes
cg06825878	chr7	75472539	75472540	0,000141845	0,119134218	0,149742938	0,269873231	-0,120130293					No
cg18960324	chr17	38705894	38705895	0,000142154	0,119134218	0,816299333	0,726516028	0,089783305					No
cg25371036	chr11	94500749	94500750	0,000142194	0,119134218	0,55608423	0,672239351	-0,116155121	AMOTL1	NM_13084	TSS1500	N_Shore	Yes
cg00524374	chr10	3894967	3894968	0,000142566	0,119134218	0,199602174	0,270340055	-0,07073788					Yes
cg00347775	chr22	43829830	43829831	0,00014279	0,119134218	0,900830691	0,835203167	0,065627524	MPPED1	NM_00104	Body	N_Shore	No
cg03959232	chr5	#########	#########	0,00014358	0,119327711	0,489835788	0,341233513	0,148602275	PCDHB19P	NR_001282	Body	Island	No
cg02835823	chr16	85979060	85979061	0,000144277	0,119490045	0,80318048	0,676142409	0,127038071				N_Shelf	Yes
cg03456771	chr7	56242072	56242073	0,000144663	0,119602767	0,821856967	0,714689271	0,107167697				N_Shore	No
cg07643930	chr16	2060265	2060266	0,000145811	0,12013533	0,06728906	0,123038951	-0,055749891	ZNF598	NM_17816	TSS1500	S_Shore	No
cg09284655	chr14	70317228	70317229	0,000147304	0,12115587	0,888546506	0,750081644	0,138464862				Island	No
cg01702055	chr6	13303064	13303065	0,000148589	0,1215603	0,158257451	0,272008943	-0,113751492					Yes
cg11984971	chr4	54765825	54765826	0,000148909	0,1215603	0,857218044	0,925229788	-0,068011743					No
cg12939425	chr5	#########	#########	0,000149011	0,1215603	0,946371512	0,888877798	0,057493714					No
cg04064963	chr6	#########	#########	0,000149286	0,1215603	0,431806032	0,522108846	-0,090302814	SNX9	NM_01622	TSS1500	N_Shore	No
cg15501231	chr6	31737629	31737630	0,000150106	0,12178106	0,271611665	0,342025597	-0,070413932	C6orf27	NM_02525	Body		Yes

cg23760945	chr19	11665140	11665141	0,000150624	0,121795896	0,846628035	0,726588155	0,120039879	ELOF1	NM_03237	Body		Yes
cg24851490	chr14	21423768	21423769	0,000150635	0,121795896	0,115952688	0,187659139	-0,071706451	RNASE2	NM_00293	5'UTR		No
cg18661379	chr10	#########	#########	0,000151406	0,122212234	0,111582948	0,191860016	-0,080277068	SUFU	NM_01616	3'UTR		No
cg01649611	chr2	43521066	43521067	0,00015212	0,122580725	0,076537083	0,161624873	-0,085087791	THADA;THA	NM_02206	Body;Body		Yes
cg23019125	chr17	78820390	78820391	0,000152512	0,122689485	0,857603171	0,796082261	0,06152091	RPTOR;RPT	NM_00116	Body;Body	S_Shore	No
cg16038868	chr8	10192618	10192619	0,000153246	0,123072608	0,145669961	0,217625241	-0,07195528	MSRA;MSR	NM_00113	Body;Body;	Island	No
cg03319082	chr16	1295309	1295310	0,000154776	0,123160001	0,518967502	0,665800885	-0,146833383					Yes
cg20255667	chr3	#########	#########	0,000155262	0,123160001	0,827935703	0,907637124	-0,079701421	CCDC48	NM_02476	Body	Island	No
cg09586924	chr11	#########	#########	0,000156655	0,123160001	0,122541126	0,223536235	-0,100995109	ST14	NM_02197	Body	S_Shelf	No
cg15302376	chr2	25560263	25560264	0,000156868	0,123160001	0,888585144	0,803230155	0,085354988	DNMT3A;D	NM_02255	5'UTR;5'UT	N_Shelf	No
cg22640868	chr17	26661373	26661374	0,000157008	0,123160001	0,105771702	0,16689002	-0,061118318	TNFAIP1;IF	NM_02113	TSS1500;5'	N_Shore	No
cg09382966	chr4	#########	#########	0,00015716	0,123160001	0,766667954	0,839858463	-0,073190509					No
cg09794680	chr14	24540451	24540452	0,000157467	0,123160001	0,228545878	0,312356366	-0,083810487	CPNE6	NM_00603	TSS1500		Yes
cg06294803	chr3	#########	#########	0,000157737	0,123160001	0,135651159	0,211176547	-0,075525389	EIF4G1;EIF4	NM_19824	5'UTR;5'UT	S_Shore	No
cg11961845	chr7	#########	#########	0,000157769	0,123160001	0,139898418	0,217482214	-0,077583796	AHCYL2;AH	NM_00113	1stExon;Body;1stExon;		Yes
cg18042586	chr8	22288224	22288225	0,000157806	0,123160001	0,494947855	0,60253726	-0,107589405	SLC39A14	NM_00113	Body		No
cg25354657	chr11	#########	#########	0,000158292	0,123160001	0,777532612	0,670972197	0,106560415	APLP2;APL	NR_024516	Body;Body;	N_Shelf	No
cg25771113	chr3	30140787	30140788	0,000158372	0,123160001	0,18772762	0,294776189	-0,107048569					No
cg11869007	chr12	52215216	52215217	0,000158544	0,123160001	0,138176955	0,35265617	-0,214479215	FIGNL2	NM_00101	1stExon	Island	No
cg26485159	chr5	4511754	4511755	0,000158876	0,123160001	0,893613926	0,825422896	0,06819103					No
cg11106652	chr4	56919046	56919047	0,000159679	0,123175607	0,342883767	0,462182218	-0,119298451				S_Shelf	Yes
cg03337057	chr19	50249775	50249776	0,000159838	0,123175607	0,63193732	0,428366365	0,203570956	TSKS	NM_02173	Body	Island	No
cg05972216	chr4	#########	#########	0,000160088	0,123175607	0,241924453	0,333756424	-0,091831971					No
cg03227963	chr5	74354834	74354835	0,000160552	0,123334228	0,880610506	0,80999689	0,070613616					No
cg15641364	chr1	#########	#########	0,000161707	0,123829994	0,14623598	0,285885569	-0,13964959	TAGLN2	NM_00356	5'UTR	N_Shelf	No
cg02606840	chr2	99280930	99280931	0,000161924	0,123829994	0,062539045	0,118343417	-0,055804371	MGAT4A;M	NM_00116	TSS1500;Body		No
cg05894970	chr3	#########	#########	0,000162496	0,123829994	0,162384755	0,25881045	-0,096425695	CDGAP	NM_02075	Body	N_Shore	No
cg08362785	chr22	40814878	40814879	0,000162864	0,123912991	0,727379445	0,642056453	0,085322992	MKL1	NM_02083	Body	Island	Yes
cg11524400	chr11	1778523	1778524	0,000163898	0,124150516	0,785976557	0,69843106	0,087545497	HCCA2;CTS	NM_05300	Body;Body	S_Shelf	No
cg22437405	chr1	38000170	38000171	0,000163909	0,124150516	0,369418214	0,496040563	-0,126622348					No
cg01264106	chr22	38071602	38071603	0,000163957	0,124150516	0,097602373	0,167241297	-0,069638924	LGALS1	NM_00230	TSS200	N_Shore	Yes
cg17969271	chr5	#########	#########	0,000164776	0,124367505	0,241148551	0,346058051	-0,1049095	FBXW11;FB	NM_03364	Body;Body;	N_Shelf	Yes
cg02100629	chr10	71892760	71892761	0,00016511	0,124367505	0,11716664	0,192426326	-0,075259686	AIFM2	NM_03279	TSS200	Island	No
cg04347414	chr1	2084519	2084520	0,000165111	0,124367505	0,771312556	0,647304796	0,12400776	PRKCZ;PRK	NM_00103	Body;Body;	S_Shore	Yes
cg00437239	chr6	28173237	28173238	0,000165287	0,124367505	0,920913938	0,826253356	0,094660582				N_Shelf	No
cg20758219	chr3	#########	#########	0,000166835	0,124913187	0,942199369	0,835776255	0,106423114	PRR23B;PR	NM_00101	1stExon;3'U	N_Shore	No
cg20315690	chr11	#########	#########	0,000167321	0,124913187	0,275152088	0,205030818	0,07012127				N_Shelf	No
cg23980070	chr18	45568066	45568067	0,000168732	0,125558798	0,233967357	0,327979229	-0,094011872	ZBTB7C	NM_00103	TSS1500		No
cg04094791	chr1	#########	#########	0,000169028	0,125558798	0,876877391	0,71232051	0,164556881	HSPA7	NR_024151	Body	Island	No
cg04663285	chr11	379455	379456	0,000169173	0,125558798	0,916222108	0,768605841	0,147616267	B4GALNT4	NM_17853	Body	Island	No
cg14396008	chr2	#########	#########	0,000169239	0,125558798	0,071317205	0,133376519	-0,062059314				Island	No
cg05805445	chr4	#########	#########	0,000170283	0,125831363	0,892111239	0,819516523	0,072594716	SPATA5	NM_14520	Body		No
cg12586707	chr4	74738902	74738903	0,000170592	0,125831363	0,152532021	0,245091726	-0,092559705				S_Shelf	No
cg24004483	chr1	944783	944784	0,000170818	0,125831363	0,202333344	0,329609735	-0,127276391				N_Shelf	Yes
cg17969540	chr19	48908179	48908180	0,00017126	0,125883878	0,920487278	0,821378467	0,099108811	GRIN2D	NM_00083	Body	Island	No

cg17004025	chr19	840795	840796	0,000171826	0,125954011	0,194120243	0,277560078	-0,083439835	PRTN3	NM_00277	TSS200	N_Shelf	No
cg06940110	chr2	#########	#########	0,000171884	0,125954011	0,625302797	0,397227379	0,228075418				N_Shore	No
cg07553761	chr3	#########	#########	0,00017267	0,125964006	0,436815647	0,299888499	0,136927147	TRIM59	NM_17308	TSS1500	Island	Yes
cg26361533	chr12	2445560	2445561	0,000173489	0,125964006	0,141759613	0,214325381	-0,072565768	CACNA1C;C	NM_00112	Body;Body;Body;Body;(		No
cg15814508	chr3	#########	#########	0,000173904	0,125964006	0,146581168	0,235865647	-0,089284479	P2RY14;ME	NM_01487	1stExon;Body;5'UTR		No
cg21196487	chr1	#########	#########	0,000174021	0,125964006	0,164603343	0,241949796	-0,077346452	S100A2	NM_00597	TSS1500		No
cg26685941	chr13	95952902	95952903	0,000174658	0,125964006	0,146884678	0,228140384	-0,081255706	ABCC4;ABC	NM_00110	Body;Body	N_Shore	Yes
cg16594502	chr15	40339900	40339901	0,000175374	0,125964006	0,169103118	0,237938941	-0,068835823					Yes
cg08573701	chr8	53603034	53603035	0,000175664	0,125964006	0,928141035	0,842186508	0,085954527	RB1CC1;RB	NM_00108	5'UTR;5'UTR		No
cg07396047	chr16	87735076	87735077	0,000176259	0,125964006	0,918314715	0,8420151	0,076299615	LOC100129	NR_024488	Body	N_Shore	No
cg17232476	chr11	#########	#########	0,000176427	0,125964006	0,940455664	0,886659556	0,053796107	SORL1	NM_00310	Body		No
cg24547396	chr22	19928740	19928741	0,00017688	0,125964006	0,220321327	0,295386617	-0,07506529	COMT;TXNI	NM_00075	TSS1500;Bo	N_Shore	Yes
cg11231143	chr17	26554245	26554246	0,000177222	0,125964006	0,803819135	0,666822239	0,136996895	PYY2	NR_003064	Body	Island	No
cg12782294	chr3	#########	#########	0,000177898	0,125964006	0,143518071	0,214091692	-0,070573621	PLD1;PLD1	NM_00113	5'UTR;5'UTR		Yes
cg25566285	chr7	#########	#########	0,000177988	0,125964006	0,920744722	0,853584153	0,067160569	PTPRN2;PT	NM_00284	Body;Body;	S_Shelf	No
cg06071604	chr13	#########	#########	0,000178436	0,125964006	0,77524211	0,673823833	0,101418277					No
cg01401327	chr7	#########	#########	0,000178722	0,125964006	0,156980049	0,211529503	-0,054549454				N_Shore	No
cg14143304	chr9	#########	#########	0,000178763	0,125964006	0,75230176	0,906840725	-0,154538966					No
cg11693709	chr15	40542018	40542019	0,000179946	0,126181557	0,318260824	0,461020965	-0,142760141	PAK6;PAK6	NM_02016	5'UTR;5'UT	N_Shelf	Yes
cg16289618	chr6	29705939	29705940	0,000180056	0,126181557	0,224078798	0,32154813	-0,097469332	LOC285830	NR_026972	Body;Body		Yes
cg08936817	chr1	20592915	20592916	0,000180322	0,126181557	0,794001731	0,872294681	-0,078292951					No
cg08553327	chr6	31543646	31543647	0,000180394	0,126181557	0,110101327	0,182891326	-0,072789999	TNF	NM_00059	1stExon		No
cg13013841	chr17	18281021	18281022	0,000182658	0,127391329	0,154520356	0,242111002	-0,087590646	EVPLL	NM_00114	TSS200		No
cg19827346	chr6	#########	#########	0,000184409	0,127920195	0,907404281	0,851891068	0,055513213	SYNE1;SYNI	NM_18296	Body;Body		No
cg19029904	chr13	79161619	79161620	0,000184488	0,127920195	0,24002338	0,148587326	0,091436055					No
cg09122593	chr1	#########	#########	0,000185609	0,128173818	0,212033809	0,300941806	-0,088907997	PPFIA4	NM_01505	TSS200		Yes
cg18882687	chr1	#########	#########	0,000185658	0,128173818	0,15546639	0,245712989	-0,090246599	ARF1;ARF1;	NM_00165	5'UTR;5'UTR;5'UTR;5'U		Yes
cg03290131	chr10	#########	#########	0,000185694	0,128173818	0,066747569	0,133417346	-0,066669777	DUSP5	NM_00441	Body		Yes
cg02845204	chr11	71259439	71259440	0,00018594	0,128173818	0,235980273	0,339612276	-0,103632003	KRTAP5-9	NM_00555	TSS200		No
cg15654264	chr1	#########	#########	0,000186466	0,128173818	0,55283822	0,683279581	-0,130441361	RPRD2	NM_01520	Body	S_Shelf	No
cg23365392	chr7	99970073	99970074	0,000189086	0,128970275	0,08534368	0,143319849	-0,057976169	PILRA;PILRA	NM_17827	TSS1500;TSS1500;TSS1		No
cg12476487	chr6	#########	#########	0,000189171	0,128970275	0,916872344	0,838176691	0,078695653	UTRN	NM_00712	Body		No
cg10399005	chr14	70316897	70316898	0,000189869	0,128970275	0,906017247	0,779184234	0,126833014				Island	No
cg14257519	chr4	#########	#########	0,000189936	0,128970275	0,890912926	0,949369146	-0,058456221	RNF150	NM_02072	Body		No
cg10309886	chr20	57407747	57407748	0,000190078	0,128970275	0,111004938	0,173928083	-0,062923145	GNASAS	NR_002785	Body		No
cg25375916	chr3	#########	#########	0,000190329	0,128970275	0,190395343	0,293164877	-0,102769534	SLC33A1	NM_00473	Body	N_Shelf	Yes
cg23691006	chr17	1510665	1510666	0,000190979	0,129202377	0,102112234	0,182875782	-0,080763548	SLC43A2	NM_15234	Body	S_Shore	No
cg17171539	chr1	59398690	59398691	0,000191213	0,129202377	0,139435738	0,236030352	-0,096594614					No
cg16230121	chr11	6435088	6435089	0,000192916	0,129601635	0,879875671	0,800806554	0,079069117	APBB1;APB	NM_00116	5'UTR;5'UTR		No
cg00186954	chr11	8933980	8933981	0,000193186	0,129601635	0,832637003	0,917309316	-0,084672313	ST5;C11orf	NM_00541	TSS1500;Bo	S_Shore	No
cg25936818	chr10	#########	#########	0,000193306	0,129601635	0,909834522	0,965302192	-0,05546767	ZRANB1;ZR	NM_01758	1stExon;5'UTR		No
cg11002686	chr7	55412681	55412682	0,000193417	0,129601635	0,7458095	0,466008212	0,279801287				Island	No
cg07474842	chr6	#########	#########	0,000193653	0,129601635	0,09457191	0,15988107	-0,065309161	MAP3K5	NM_00592	Body		No
cg01464515	chr10	72254322	72254323	0,000193705	0,129601635	0,162190377	0,282471089	-0,120280711	KIAA1274	NM_01443	5'UTR		No
cg09490277	chr6	35419590	35419591	0,0001941	0,129615507	0,128370727	0,197235503	-0,068864777	FANCE	NM_02192	TSS1500	N_Shore	No

cg27193080	chr3	#########	#########	0,000194269	0,129615507	0,682530876	0,788206595	-0,105675719	IFT122;IFT1	NM_05299	Body;Body;	S_Shore	No
cg19144019	chr17	26875307	26875308	0,000194976	0,129724217	0,166995135	0,24652575	-0,079530615	UNC119;UN	NM_05403	Body;Body	N_Shelf	No
cg18222500	chr17	143285	143286	0,000195798	0,129908382	0,160591915	0,238990486	-0,078398571	RPH3AL	NM_00698	Body		No
cg07728631	chrY	14100067	14100068	0,000197765	0,130955559	0,950359813	0,874656551	0,075703262				Island	No
cg21248060	chr7	1039956	1039957	0,000200763	0,132459647	0,911408113	0,825528782	0,085879331	C7orf50;C7	NM_00113	Body;Body;	Island	No
cg04148285	chr16	89976787	89976788	0,000201358	0,132459647	0,1499188	0,205560413	-0,055641614	TCF25	NM_01497	Body	N_Shore	No
cg02849507	chr16	14051717	14051718	0,000201634	0,132459647	0,250952462	0,343006717	-0,092054255					No
cg01590848	chr19	39997621	39997622	0,000201852	0,132459647	0,381261663	0,238423519	0,142838145	DLL3;DLL3	NM_20348	Body;Body	Island	No
cg06437100	chr16	21767741	21767742	0,000202703	0,132542888	0,090487566	0,166103272	-0,075615707	OTOA;OTO	NM_17066	Body;Body;	N_Shore	No
cg01375994	chrX	3261645	3261646	0,0002035	0,132542888	0,651102759	0,390127827	0,260974931	MXRA5	NM_01541	Body	N_Shore	No
cg09697644	chr16	34375039	34375040	0,000203511	0,132542888	0,859658969	0,732545925	0,127113044					No
cg13105872	chr12	38447229	38447230	0,000203541	0,132542888	0,835874889	0,744495649	0,09137924					No
cg02470871	chr3	#########	#########	0,000204343	0,132542888	0,13189844	0,211545568	-0,079647128	CD80	NM_00519	TSS200		No
cg17082225	chr7	56673048	56673049	0,000205469	0,132542888	0,805260932	0,587122995	0,218137937					No
cg16997203	chr11	44972678	44972679	0,000205593	0,132542888	0,108552769	0,162007788	-0,053455019	TP53I11;TP	NM_00603	TSS200;TSS	Island	No
cg21254135	chr7	76852292	76852293	0,000205638	0,132542888	0,854143243	0,767530506	0,086612736	CCDC146	NM_02087	Body		No
cg12126706	chr1	#########	#########	0,000205822	0,132542888	0,28654549	0,421274785	-0,134729295	C1orf92	NM_14470	TSS1500	N_Shore	Yes
cg21691367	chr6	#########	#########	0,0002062	0,132542888	0,203489492	0,298724214	-0,095234721	MTHFD1L	NM_01544	Body		Yes
cg00464814	chr6	16758888	16758889	0,000206779	0,132542888	0,159213847	0,234532255	-0,075318407	ATXN1;ATX	NM_00112	5'UTR;5'UT	N_Shore	Yes
cg14715778	chr17	73840989	73840990	0,000206985	0,132542888	0,13804665	0,205596531	-0,06754988	UNC13D	NM_19924	TSS200		Yes
cg03905134	chr12	76146565	76146566	0,000207133	0,132542888	0,872543763	0,938890461	-0,066346698					No
cg01154505	chr2	#########	#########	0,000207344	0,132542888	0,882013495	0,795978533	0,086034962	FBLN7;FBL	NM_15321	Body;Body	S_Shore	Yes
cg02135077	chr8	#########	#########	0,000208164	0,132542888	0,060250828	0,117577337	-0,057326509				N_Shore	No
cg03655330	chr3	50337494	50337495	0,000208387	0,132542888	0,112470067	0,175631202	-0,063161135	HYAL1;HYA	NM_00731	3'UTR;3'UT	S_Shore	Yes
cg12403889	chr1	11753338	11753339	0,000208562	0,132542888	0,091575108	0,147766459	-0,056191351	C1orf187	NM_19854	5'UTR	S_Shore	No
cg18996916	chr8	1054785	1054786	0,000208632	0,132542888	0,912277814	0,862222402	0,050055412				S_Shore	No
cg11619648	chr3	44515241	44515242	0,000208823	0,132542888	0,884488523	0,938136098	-0,053647575	ZNF445	NM_18148	5'UTR	N_Shelf	No
cg04326808	chr15	39543042	39543043	0,000209273	0,132579288	0,538173479	0,630127224	-0,091953745	C15orf54;C	NM_20744	1stExon;5'UTR		Yes
cg14386496	chr4	66536463	66536464	0,000214052	0,13477364	0,197075227	0,121657333	0,075417894	EPHA5;EPH	NM_00443	TSS1500;TS	Island	No
cg00816037	chr16	88812341	88812342	0,000214078	0,13477364	0,059298095	0,110539362	-0,051241267	FAM38A	NM_00114	Body	N_Shelf	No
cg19146112	chr6	74072255	74072256	0,000214149	0,13477364	0,934036541	0,868025377	0,066011165	C6orf221	NM_00101	TSS200	N_Shore	No
cg11034978	chr11	64837009	64837010	0,000214696	0,134816078	0,074137242	0,12614116	-0,052003917					No
cg00663986	chr17	80866231	80866232	0,000215105	0,134816078	0,880512988	0,822907144	0,057605843	TBCD	NM_00599	Body	S_Shelf	No
cg18110741	chr14	64419267	64419268	0,00021551	0,134816078	0,835275039	0,887443405	-0,052168367	SYNE2;SYN	NM_18291	Body;Body		No
cg23088142	chr3	#########	#########	0,000215538	0,134816078	0,697265439	0,5547134	0,142552039					No
cg09888026	chr17	79303433	79303434	0,000215921	0,134816078	0,168275586	0,247874365	-0,07959878	TMEM105	NM_17852	5'UTR		No
cg02963266	chr14	99681709	99681710	0,000216095	0,134816078	0,809888568	0,720659408	0,089229159	BCL11B;BCL	NM_02289	Body;Body		No
cg18216249	chr12	48100804	48100805	0,000216195	0,134816078	0,118642554	0,170114063	-0,051471509	RPAP3;RPA	NM_00114	TSS1500;TS	S_Shore	No
cg05756685	chr1	12571625	12571626	0,000217648	0,134952115	0,912597426	0,853232165	0,059365261	VPS13D;VP	NM_01815	3'UTR;3'UTR		No
cg13807549	chr9	#########	#########	0,000217761	0,134952115	0,250748706	0,33679481	-0,086046105					No
cg12371569	chr12	13278271	13278272	0,000217842	0,134952115	0,132218173	0,228458193	-0,09624002					No
cg16328023	chr7	36382071	36382072	0,000217874	0,134952115	0,132119677	0,2422884	-0,110168724	KIAA0895;K	NM_01531	Body;Body		No
cg18052511	chr3	46410221	46410222	0,000219156	0,135423829	0,06082555	0,144912756	-0,084087206	CCR5;CCR5	NM_00057	TSS1500;TSS1500		No
cg02779913	chr17	40088679	40088680	0,00021983	0,135489043	0,096832733	0,19001532	-0,093182587	TTC25	NM_03142	Body	S_Shore	Yes
cg16685608	chr14	52211578	52211579	0,000220174	0,135526197	0,932184422	0,803574151	0,128610271					No

cg16971827	chr11	#########	#########	0,000220528	0,135569061	0,845090368	0,749881716	0,095208652	CBL	NM_00518	3'UTR	N_Shelf	No
cg05897169	chr17	33568597	33568598	0,000221158	0,135592147	0,863959843	0,761346012	0,102613831	SLFN5	NM_14497	TSS1500	N_Shore	No
cg09597645	chr12	34471143	34471144	0,000222056	0,135592147	0,748421984	0,613256757	0,135165227					No
cg07865444	chr8	47866659	47866660	0,000222228	0,135592147	0,895120342	0,828961991	0,066158351				Island	No
cg14519515	chr11	67033594	67033595	0,000224418	0,135691933	0,133986387	0,200098122	-0,066111736	ADRBK1	NM_00161	TSS1500	N_Shore	Yes
cg07328519	chr19	4064300	4064301	0,000227115	0,136625222	0,211126261	0,311712242	-0,100585981	ZBTB7A	NM_01589	5'UTR	Island	Yes
cg08726522	chr11	8739586	8739587	0,000229606	0,137603189	0,398036719	0,492655282	-0,094618564	ST5;ST5;ST5	NM_13915	Body;Body;Body		No
cg02087075	chr6	44226395	44226396	0,000231392	0,138154422	0,892478557	0,840335641	0,052142916	SLC35B2;NI	NM_17814	TSS1500;3'U	S_Shore	No
cg18471664	chr3	5028143	5028144	0,000232877	0,138154422	0,173392954	0,261122479	-0,087729525				N_Shore	No
cg09286421	chr7	55552130	55552131	0,000233132	0,138154422	0,90541248	0,839906376	0,065506104	VOPP1	NM_03079	Body		No
cg27515052	chr8	8513104	8513105	0,00023416	0,138419863	0,920603091	0,867884865	0,052718226					No
cg21626540	chr2	10720198	10720199	0,000235721	0,139170184	0,750301167	0,642584767	0,1077164	NOL10	NM_02489	Body		No
cg25187161	chr1	29510025	29510026	0,000236438	0,139207458	0,168034143	0,260178983	-0,09214484	SFRS4	NM_00562	TSS1500	S_Shore	Yes
cg18322589	chr10	#########	#########	0,000236855	0,139207458	0,852034956	0,721984044	0,130050912	TACC2;TAC	NM_20686	Body;Body		No
cg24996985	chr2	#########	#########	0,000237131	0,139207458	0,652894691	0,486866256	0,166028435					No
cg25179876	chr10	33483108	33483109	0,000237577	0,139207458	0,192661727	0,311734018	-0,119072291	NRP1	NM_00387	Body		Yes
cg22108567	chr7	5535582	5535583	0,000238062	0,139207458	0,1560138	0,230197258	-0,074183458	MIR589;FB	NR_030318	TSS200;Bod	S_Shore	No
cg25178683	chr17	76976267	76976268	0,00023832	0,139207458	0,677148253	0,570408242	0,106740012	LGALS3BP	NM_00556	TSS1500		No
cg13501951	chr7	#########	#########	0,000238411	0,139207458	0,496825541	0,37219494	0,124630601				Island	No
cg00640087	chr6	31707202	31707203	0,000238957	0,139355784	0,088741842	0,147409658	-0,058667816	MSH5;MSH	NM_02525	TSS1500;TS	N_Shore	No
cg26269286	chr14	#########	#########	0,000241101	0,139423056	0,158313745	0,251519707	-0,093205962				N_Shelf	Yes
cg19848940	chr12	#########	#########	0,000241727	0,139605804	0,162927518	0,239515715	-0,076588197					Yes
cg23469878	chr9	#########	#########	0,000242281	0,139641057	0,425240677	0,576310177	-0,1510695	LCN6	NM_19894	Body	Island	Yes
cg13484324	chr2	#########	#########	0,000242809	0,139641057	0,936884822	0,88487724	0,052007582					No
cg04264075	chr19	51876469	51876470	0,000243374	0,139641057	0,177076297	0,259531392	-0,082455095	NKG7	NM_00560	TSS1500		No
cg02640809	chr5	#########	#########	0,000243454	0,139641057	0,129268857	0,204268882	-0,075000025					No
cg02208313	chr17	80085331	80085332	0,000244262	0,139884292	0,841675058	0,757984013	0,083691044	CCDC57	NM_19808	Body	Island	No
cg00034154	chr19	10337162	10337163	0,000244951	0,140110627	0,762854197	0,851529509	-0,088675312	S1PR2	NM_00423	5'UTR	S_Shore	No
cg06603309	chr11	2724143	2724144	0,000246393	0,140470892	0,922183286	0,854907022	0,067276264	KCNQ1;KCN	NM_00021	Body;Body	S_Shelf	No
cg15657936	chr4	8583362	8583363	0,000247331	0,140470892	0,868409899	0,726799472	0,141610428	GPR78	NM_08081	1stExon	Island	No
cg10517650	chr3	#########	#########	0,000247655	0,140470892	0,466452786	0,312921972	0,153530814	CCDC52	NM_14471	TSS1500	S_Shore	No
cg26332114	chr19	57742259	57742260	0,000248514	0,140470892	0,917008203	0,759951355	0,157056848	AURKC;AUI	NM_00101	TSS200;TSS	Island	No
cg01791648	chr3	#########	#########	0,000248739	0,140470892	0,674059996	0,775777997	-0,101718	SENP7;SEN	NM_00107	TSS1500;TS	S_Shore	No
cg04797957	chr17	76840488	76840489	0,000248784	0,140470892	0,11431936	0,170735184	-0,056415825				S_Shelf	No
cg07216884	chr1	#########	#########	0,000248862	0,140470892	0,902436822	0,803261778	0,099175045					No
cg07671036	chr3	46875766	46875767	0,000249211	0,140470892	0,96790946	0,898105599	0,069803862	PRSS42	NM_18270	TSS200	Island	No
cg14925024	chr1	#########	#########	0,000250439	0,1407192	0,80016022	0,649180838	0,150979383	C1orf35	NM_02431	TSS1500	S_Shore	No
cg18739675	chr2	43041124	43041125	0,00025196	0,140909649	0,164525135	0,28876499	-0,124239855				S_Shelf	Yes
cg25784219	chr2	28618327	28618328	0,000253318	0,141332696	0,143946785	0,228443952	-0,084497168	FOSL2	NM_00525	Body	S_Shore	No
cg03808001	chr13	#########	#########	0,000253535	0,141332696	0,159784369	0,325801785	-0,166017416					No
cg17016369	chr2	#########	#########	0,000253622	0,141332696	0,266816643	0,347848425	-0,081031782					No
cg07679948	chr12	56329641	56329642	0,000253901	0,141332696	0,895069014	0,813760427	0,081308587	DGKA;DGK	NM_20144	5'UTR;5'UT	Island	No
cg21111416	chr3	#########	#########	0,000255925	0,141490469	0,912603239	0,836742909	0,075860331	HLTF;HLTF	NM_13904	TSS1500;TS	S_Shore	No
cg16104584	chr1	9149733	9149734	0,000256206	0,141490469	0,236829259	0,352448968	-0,115619709	SLC2A5	NR_024180	TSS1500		No
cg17864046	chr6	#########	#########	0,000256412	0,141490469	0,322820511	0,22188732	0,100933191				S_Shore	No

cg21118486	chr6	#########	#########	0,000257304	0,141686639	0,931892167	0,87081035	0,061081818	RPS6KA2	NM_00100	Body		No
cg10898024	chr2	40266365	40266366	0,000258635	0,141686639	0,218889209	0,348794713	-0,129905504					Yes
cg15177613	chr19	50249736	50249737	0,000259151	0,141686639	0,553785028	0,37233798	0,181447048	TSKS	NM_02173	Body	Island	No
cg18725375	chr11	86142478	86142479	0,00025923	0,141686639	0,904449977	0,773434623	0,131015354				Island	No
cg11119313	chr17	61551779	61551780	0,000259424	0,141686639	0,609077726	0,690937919	-0,081860194				N_Shelf	Yes
cg11940177	chr10	99188478	99188479	0,000259608	0,141686639	0,161137254	0,25814774	-0,097010487	PGAM1	NM_00262	Body	S_Shelf	Yes
cg22617819	chr1	44378781	44378782	0,0002602	0,141686639	0,667767919	0,739916085	-0,072148166	ST3GAL3;ST	NM_17496	Body;Body;Body;Body;(		Yes
cg18739950	chr15	95870439	95870440	0,000260221	0,141686639	0,226428243	0,146570296	0,079857946				S_Shore	No
cg15863827	chr10	79363894	79363895	0,000260477	0,141686639	0,349126821	0,431767995	-0,082641174	KCNMA1;K(	NM_00116	Body;Body;Body;Body		No
cg24481594	chr1	2190849	2190850	0,000261695	0,141834017	0,911021458	0,832880936	0,078140522	SKI	NM_00303	Body		No
cg08612539	chr22	37257124	37257125	0,000261922	0,141834017	0,104157114	0,168919019	-0,064761905	NCF4;NCF4	NM_00063	5'UTR;1stExon;5'UTR;1		No
cg14588003	chr15	93361889	93361890	0,000262235	0,141834017	0,911841842	0,841051926	0,070789917					No
cg05620821	chr11	67777618	67777619	0,000263846	0,142276646	0,120102964	0,198041169	-0,077938206	ALDH3B1;A	NM_00103	TSS200;TSS200;5'UTR		No
cg05039475	chr3	35680791	35680792	0,000264363	0,142339588	0,208047381	0,151819758	0,056227623				Island	No
cg04244970	chr1	#########	#########	0,000265627	0,142503353	0,131333673	0,218206664	-0,086872991	SLAMF7	NM_02118	TSS200		Yes
cg06190807	chr1	62660187	62660188	0,000267605	0,142955826	0,659561763	0,564837322	0,094724441	L1TD1;L1TD	NM_01907	TSS1500;TS	N_Shore	No
cg04160749	chr8	58172570	58172571	0,000269762	0,143358298	0,909369826	0,798857812	0,110512014				Island	No
cg07092212	chr11	46382544	46382545	0,000269818	0,143358298	0,082204156	0,133325626	-0,05112147	DGKZ;DGKZ	NM_20153	Body;TSS1500;Body;Bo		Yes
cg24710951	chr6	30656530	30656531	0,000269861	0,143358298	0,905809889	0,829341789	0,0764681	KIAA1949;N	NM_00113	TSS1500;Bo	S_Shore	No
cg25221207	chr11	#########	#########	0,000271088	0,143526821	0,155808117	0,234213674	-0,078405557	PVRL1;PVRI	NM_00285	Body;Body;Body		Yes
cg10616795	chr5	76464212	76464213	0,000271136	0,143526821	0,709820001	0,809000999	-0,099180999					Yes
cg02304751	chr4	99583944	99583945	0,000271382	0,143526821	0,916896854	0,852517742	0,064379112				S_Shelf	No
cg14901243	chr19	13951844	13951845	0,000273568	0,143686997	0,303886896	0,415647905	-0,11176101				N_Shore	Yes
cg04794141	chr14	92325158	92325159	0,000273861	0,143686997	0,165792697	0,281833563	-0,116040865	TC2N	NM_00112	5'UTR		No
cg11321083	chr11	64638189	64638190	0,000273995	0,143686997	0,216678277	0,307798045	-0,091119768	EHD1	NM_00679	Body		Yes
cg26666978	chr21	47038530	47038531	0,000274095	0,143686997	0,175075749	0,284354286	-0,109278537					Yes
cg07555125	chr5	#########	#########	0,000275946	0,144340301	0,949003195	0,869872676	0,079130519	PCDHA1;PC	NM_03141	Body;Body;	Island	No
cg10565662	chr8	2670187	2670188	0,000276843	0,14446629	0,812802505	0,720276961	0,092525544					No
cg03936135	chr16	86371247	86371248	0,000277095	0,14446629	0,342848987	0,500880328	-0,158031341	LOC732275	NR_024406	Body		No
cg23569941	chr2	#########	#########	0,0002776	0,144471081	0,844839097	0,915917887	-0,071078791	TMBIM1;PN	NM_02215	3'UTR;Body	S_Shelf	No
cg12019961	chr11	#########	#########	0,00027771	0,144471081	0,161229045	0,10810773	0,053121315	ATM;NPAT	NM_00005	TSS1500;Bo	N_Shore	No
cg03077671	chr19	13211225	13211226	0,000278846	0,144860395	0,081972464	0,141342523	-0,059370059	LYL1	NM_00558	Body	S_Shore	No
cg04381957	chr3	16550461	16550462	0,000279484	0,144860395	0,146045703	0,243151119	-0,097105416	RFTN1	NM_01515	5'UTR	N_Shelf	Yes
cg25223354	chr10	#########	#########	0,000279559	0,144860395	0,895333578	0,750033449	0,145300128				N_Shelf	No
cg09914404	chr17	14215441	14215442	0,000279977	0,144860395	0,230603754	0,347690883	-0,117087129	HS3ST3B1	NM_00604	Body	S_Shelf	No
cg18776609	chrX	41138825	41138826	0,000280651	0,144895124	0,82860152	0,880423089	-0,051821569				S_Shelf	No
cg22595230	chr20	3766560	3766561	0,000281083	0,144961014	0,626380172	0,795587125	-0,169206953	CENPB	NM_00181	1stExon	Island	No
cg25776343	chr10	72336096	72336097	0,00028197	0,14511682	0,173723432	0,273298209	-0,099574778					No
cg19663246	chr7	32339095	32339096	0,000281993	0,14511682	0,452540679	0,572039003	-0,119498324				S_Shore	Yes
cg16263152	chr1	#########	#########	0,000282777	0,145199154	0,216003214	0,318814649	-0,102811435	NMNAT2	NM_01503	Body		No
cg21808406	chr17	7482475	7482476	0,000283339	0,145199154	0,226404181	0,313046128	-0,086641947	CD68;CD68	NM_00104	TSS1500;TS	N_Shelf	No
cg00232387	chr19	2717526	2717527	0,000284945	0,145199154	0,864083795	0,774055828	0,090027966	DIRAS1	NM_14517	Body	Island	No
cg11291464	chr10	#########	#########	0,000285272	0,145199154	0,789238216	0,683730669	0,105507548					No
cg00100420	chr4	#########	#########	0,000285386	0,145199154	0,106873563	0,195169191	-0,088295628					No
cg07195224	chr1	#########	#########	0,000285457	0,145199154	0,132356145	0,204485127	-0,072128983	AIM2	NM_00483	TSS1500		Yes

cg14244577	chr16	70332877	70332878	0,000285672	0,145199154	0,22935699	0,297617202	-0,068260213	DDX19B;DD	NM_00724	TSS200;TSS200;TSS200		Yes
cg09300089	chr16	89539229	89539230	0,00028611	0,145199154	0,118075237	0,196539837	-0,0784646	ANKRD11	NM_01327	5'UTR		No
cg25065097	chr13	50160349	50160350	0,000286421	0,145202484	0,187479911	0,267199543	-0,079719632	RCBTB1	NM_01819	TSS1500	S_Shore	No
cg04084348	chr10	75677010	75677011	0,000287908	0,145438585	0,18486421	0,276421604	-0,091557394	PLAU;C10o	NM_00114	3'UTR;5'UTR;3'UTR		No
cg06621480	chr22	49812410	49812411	0,000288169	0,145438585	0,885233569	0,78629539	0,098938179				Island	No
cg00920314	chrX	84189177	84189178	0,00028843	0,145438585	0,821487495	0,634837501	0,186649994	UBE2DNL	NR_024062	Body	Island	No
cg25096107	chr14	#########	#########	0,000288716	0,145438585	0,939706368	0,876354479	0,063351889				N_Shelf	No
cg21703988	chr12	#########	#########	0,000289518	0,145688529	0,811383642	0,728550935	0,082832707	EP400	NM_01540	Body		No
cg22544881	chr7	#########	#########	0,000291855	0,146555623	0,192420406	0,286648805	-0,0942284	FLJ43663;F	NR_015431	Body;Body		Yes
cg19611886	chr8	#########	#########	0,00029573	0,147632374	0,169643251	0,284407211	-0,11476396				N_Shore	No
cg25367249	chr12	96127071	96127072	0,000295786	0,147632374	0,833190379	0,895287084	-0,062096704	NTN4	NM_02122	Body		No
cg08541155	chr3	#########	#########	0,000295856	0,147632374	0,221105635	0,313195938	-0,092090303					Yes
cg22082462	chr14	24540415	24540416	0,000296257	0,147677974	0,481629894	0,610241283	-0,128611389	CPNE6	NM_00603	TSS1500		Yes
cg18865685	chr1	#########	#########	0,000296711	0,147737499	0,802451616	0,615375823	0,187075793	ASTN1;AST	NM_20710	Body;Body		No
cg17529386	chr6	#########	#########	0,000299385	0,148153241	0,889967549	0,749032608	0,140934941				Island	No
cg17094065	chr12	53443583	53443584	0,000300864	0,148527168	0,494061703	0,631096364	-0,137034662	TENC1;TEN	NM_17075	TSS1500;5'	S_Shore	No
cg13897122	chr2	#########	#########	0,000301539	0,148527168	0,142841539	0,243240739	-0,1003992	IL18RAP	NM_00385	5'UTR		No
cg17749946	chr6	22570547	22570548	0,000301799	0,148527168	0,947098111	0,888778838	0,058319273	HDGFL1	NM_13857	1stExon	Island	No
cg26692003	chr3	13063165	13063166	0,000302009	0,148527168	0,893257624	0,808042145	0,085215479	IQSEC1	NM_00113	Body		No
cg14474293	chr15	95821440	95821441	0,000302625	0,14867702	0,8423478	0,894689681	-0,05234188					No
cg22525294	chr19	50249463	50249464	0,000304702	0,149204509	0,409809354	0,205202654	0,204606701	TSKS	NM_02173	Body	N_Shore	No
cg21439672	chr12	7260546	7260547	0,000305375	0,149204509	0,325578022	0,420906351	-0,095328329	C1RL;LOC28	NM_01654	Body;TSS1500		No
cg20386404	chr1	#########	#########	0,000305615	0,149204509	0,213765234	0,287634367	-0,073869133	PTPN14	NM_00540	Body		No
cg00786084	chr3	45942459	45942460	0,000305729	0,149204509	0,891644254	0,831020503	0,060623751	CCR9;CCR9	NM_03120	Body;Body		No
cg20711383	chr12	62995960	62995961	0,000306203	0,149205605	0,109002014	0,17972527	-0,070723256	C12orf61;C	NM_17589	3'UTR;1stE	N_Shore	No
cg03239580	chr6	31973051	31973052	0,000307638	0,149737417	0,17884229	0,235997784	-0,057155494	CYP21A2;C	NM_00050	TSS1500;TSS1500		No
cg24293507	chr1	10511792	10511793	0,000307922	0,149737417	0,251043607	0,331335806	-0,080292199	APITD1;CO	NM_19854	3'UTR;3'UTR		No
cg03303515	chr2	47213997	47213998	0,000308543	0,149886627	0,201869199	0,257824632	-0,055955433	TTC7A	NM_02045	Body		No
cg25717406	chr7	97588677	97588678	0,000310519	0,149900843	0,646090863	0,511158638	0,134932225					No
cg02488934	chr1	#########	#########	0,000311063	0,149900843	0,24468983	0,326321447	-0,081631618				N_Shelf	No
cg02005989	chr16	34431002	34431003	0,000311388	0,149900843	0,894035473	0,816430471	0,077605002				Island	No
cg05424831	chr7	#########	#########	0,000311805	0,149900843	0,918306208	0,836032672	0,082273536	ATXN7L1	NM_02072	Body		No
cg24231804	chr15	67316860	67316861	0,000311821	0,149900843	0,145739861	0,22104331	-0,075303449					Yes
cg26637171	chr19	55882549	55882550	0,00031338	0,14997658	0,81634758	0,681449697	0,134897883	IL11	NM_00064	TSS1500	S_Shore	No
cg22104744	chr13	#########	#########	0,000313837	0,14997658	0,215678113	0,316521328	-0,100843215	RASA3	NM_00736	Body		No
cg08759026	chr11	69061454	69061455	0,000314065	0,14997658	0,19457124	0,267091184	-0,072519944	MYEOV	NM_13876	TSS200		No
cg26152923	chr19	18284119	18284120	0,000314588	0,14997658	0,108051968	0,161185046	-0,053133078	IFI30	NM_00633	TSS1500	N_Shore	No
cg23484392	chr18	66465333	66465334	0,000315481	0,14997658	0,502380706	0,600485648	-0,098104942	CCDC102B;	NM_00109	5'UTR;1stExon;5'UTR		Yes
cg04826368	chr6	27130207	27130208	0,000315817	0,14997658	0,506312076	0,629333694	-0,123021618					Yes
cg14279856	chr6	30851086	30851087	0,000317123	0,14997658	0,883510999	0,82310596	0,06040504	DDR1;DDR1	NM_00195	TSS1500;TS	N_Shore	No
cg20107987	chr11	58873823	58873824	0,00031719	0,14997658	0,918521664	0,846609574	0,071912091	FAM111B;F	NM_00114	TSS1500;TS	N_Shore	No

Supplementary table 3: Significant methylated regions (sig-DMRs).
Minpval < 0.001, max delta beta > 5% and ≥ 5 CpGs in region.

gene_asso	group	no.probes	minpval	meanpval	maxbetafc	hg19coord	Age
SLC1A5	Body,5'UTR	7	2,3E-34	3,9E-33	-8,3E-02	hr19:47287778-4728826	No
BRCA1,NBR	5'UTR,Body	43	2,9E-29	5,6E-04	1,5E-01	hr17:41277213-4127902	No
FAM38A	Body	8	7,9E-25	2,8E-06	-9,8E-02	hr16:88849599-8885129	No
AHCYL2	TSS1500,Bo	8	4,3E-23	7,3E-14	-7,8E-02	hr7:129007565-12900840	Yes
LOC100129	Body	8	7,7E-23	8,3E-05	1,1E-01	hr16:87734816-8773606	Yes
AURKC	TSS1500,TS	12	1,1E-22	7,9E-05	2,3E-01	hr19:57741325-5774341	No
		7	1,8E-22	2,6E-17	1,9E-01	hr3:133502540-13350343	No
C11orf21,T	Body,TSS15	36	2,7E-22	5,7E-07	-7,1E-02	chr11:2321770-2323938	No
DDR1	TSS1500,TS	23	2,8E-21	8,8E-04	1,2E-01	chr6:30850405-30852092	Yes
		10	6,0E-20	1,8E-06	1,6E-01	hr14:70315994-7031758	No
SDHAP3	Body,TSS20	12	1,5E-19	7,8E-14	-2,2E-01	chr5:1594021-1595048	No
ITGAX	TSS1500,TS	5	1,7E-19	3,8E-19	-8,9E-02	hr16:31366118-3136653	Yes
SARS	1stExon,Bo	5	3,1E-19	4,3E-04	-8,6E-02	hr1:109756737-10975758	Yes
SLPI	Body,1stEx	6	1,2E-18	1,1E-11	-1,1E-01	hr20:43882990-4388374	Yes
SLC22A18A	Body,TSS15	21	3,7E-18	2,6E-07	-1,1E-01	chr11:2919689-2921176	Yes
TSKS	Body	6	1,3E-17	7,6E-17	2,2E-01	hr19:50249464-5024992	No
EIF4A1,CD6	3'UTR,TSS1	6	1,9E-17	7,2E-06	-9,2E-02	chr17:7482003-7483518	No
NCAPD2,G	3'UTR,TSS1	6	7,9E-17	7,0E-04	-6,6E-02	chr12:6641042-6642354	No
NOD2	TSS1500,TS	8	1,6E-16	2,2E-07	-1,1E-01	hr16:50730385-5073221	Yes
		9	1,8E-16	8,6E-14	1,9E-01	hr13:23309774-2331067	No
SLC16A3	5'UTR,TSS1	10	2,8E-16	8,7E-07	-8,7E-02	hr17:80189225-8019015	Yes
SPRED2	5'UTR,1stE	6	3,0E-16	8,2E-05	-1,6E-01	chr2:65593761-65594890	Yes
GFI1	5'UTR,1stE	27	4,5E-16	6,9E-04	-1,2E-01	chr1:92951355-92953907	Yes
		6	6,0E-16	6,5E-05	-6,3E-02	hr14:95982758-9598410	No
OXT	TSS1500,TS	13	9,9E-15	5,4E-05	-1,4E-01	chr20:3051954-3053196	Yes
		5	2,3E-14	5,1E-14	1,6E-01	hr2:240868184-24086863	No
LGALS3BP	1stExon,5'U	8	2,4E-14	2,5E-13	1,1E-01	hr17:76975944-7697635	No
		5	2,5E-14	1,1E-12	1,2E-01	r11:134582289-13458827	No
RTN3	TSS1500,TS	10	4,6E-14	9,1E-04	-1,2E-01	hr11:63448356-6344934	Yes

PTPN7	5'UTR,1stE>	12	6,8E-14	1,5E-04	-1,2E-01	hr1:202128682-20213118	Yes
LCK	5'UTR,TSS1	8	1,7E-13	1,2E-04	1,2E-01	chr1:32738251-32740758	No
AJAP1	Body	6	3,7E-13	3,6E-04	6,1E-02	chr1:4770161-4771682	No
C1RL,LOC28	Body,TSS15	7	6,4E-13	6,6E-13	-9,5E-02	chr12:7260546-7260888	Yes
LGALS1	TSS1500,TS	11	7,1E-13	1,1E-07	-9,5E-02	hr22:38071001-3807167	Yes
		7	3,8E-12	7,1E-06	-9,7E-02	hr10:74057347-7405809	No
IQSEC3	Body,5'UTR	5	6,1E-12	4,8E-04	7,3E-02	chr12:213776-215130	No
HDAC4	Body	7	6,9E-12	8,6E-04	-1,0E-01	hr2:240196078-24019713	Yes
CHFR	Body	7	7,2E-12	7,4E-11	6,8E-02	r12:133424221-1334247	Yes
RUNX3	Body,1stEx	16	9,3E-12	6,6E-04	1,4E-01	chr1:25290947-25292412	Yes
TYMP,SCO2	Body,TSS15	6	2,2E-11	5,6E-04	-5,5E-02	hr22:50964653-5096612	No
RGS12	TSS1500,Bo	10	2,7E-11	6,8E-04	7,7E-02	chr4:3371520-3373006	No
DPPA5	Body,TSS20	10	3,0E-11	5,0E-06	1,4E-01	chr6:74063522-74064594	No
		9	5,5E-11	1,2E-04	-1,2E-01	chr1:59280290-59281540	Yes
AIFM2	5'UTR,1stE>	14	5,7E-11	1,1E-06	-7,7E-02	hr10:71891928-7189336	Yes
BANP	Body	6	6,9E-11	5,9E-08	1,3E-01	hr16:88102758-8810362	Yes
LGALS12	TSS1500,TS	10	7,7E-11	1,3E-04	-9,9E-02	hr11:63272554-6327423	No
CHST12	5'UTR	5	8,5E-11	2,6E-04	-1,2E-01	chr7:2443748-2445331	Yes
		10	9,0E-11	2,9E-04	1,8E-01	r10:131843517-1318450	No
C7orf50,MI	Body,TSS20	14	1,0E-10	2,9E-04	5,4E-02	chr7:1061928-1063357	No
CHI3L2	TSS1500,TS	9	1,3E-10	5,0E-06	7,3E-02	hr1:111768837-11177071	No
SPO11	TSS1500,TS	11	1,4E-10	1,4E-09	1,2E-01	hr20:55904114-5590507	No
LAMA3	TSS1500,Bo	7	1,4E-10	3,9E-10	8,8E-02	hr18:21452730-2145313	No
		6	1,6E-10	4,3E-10	9,5E-02	hr1:117317838-11731823	No
BHLHE40	TSS1500	9	2,7E-10	7,3E-04	-7,7E-02	chr3:5018239-5019877	No
RNF39	3'UTR,Body	41	2,7E-10	7,8E-05	1,8E-01	chr6:30038712-30040291	No
FXYD6	5'UTR,1stE>	16	3,1E-10	3,5E-04	-8,8E-02	r11:117747030-1177485	Yes
NWD1	TSS1500,TS	6	3,9E-10	5,4E-05	-1,5E-01	hr19:16829985-1683085	Yes
BGLAP	TSS1500,TS	12	5,6E-10	8,2E-06	-8,9E-02	hr1:156211409-15621257	No
CSGALNAC1	Body,5'UTR	9	6,5E-10	8,0E-07	-1,3E-01	chr8:19539991-19540734	Yes
NKAPL	TSS1500,TS	13	6,9E-10	1,4E-04	1,7E-01	chr6:28226094-28227482	No
BCAT1	Body,1stEx	8	6,9E-10	6,4E-04	-1,2E-01	hr12:25101448-2510317	Yes
RCAN3	Body,3'UTR	6	8,2E-10	5,4E-08	1,1E-01	chr1:24861604-24862381	Yes
CX3CR1	Body,5'UTR	11	9,4E-10	9,9E-04	-9,4E-02	chr3:39321449-39323539	No

FOXK1	Body	6	1,1E-09	1,5E-06	6,8E-02	chr7:4778839-4779342	No
MAD1L1	Body	8	1,6E-09	3,3E-05	1,0E-01	chr7:2138435-2140321	Yes
LOC284837	Body,TSS20	5	2,1E-09	4,4E-09	-9,5E-02	hr21:45232232-4523260	Yes
		7	2,3E-09	1,3E-05	2,4E-01	chr7:56241697-56242801	No
TXNDC11	Body,1stEx	18	3,1E-09	4,5E-05	-1,2E-01	hr16:11834960-1183771	No
TP53I11	5'UTR,1stE	15	3,2E-09	4,8E-04	-5,3E-02	hr11:44971833-4497321	No
		40	4,0E-09	3,1E-05	8,2E-02	chr6:29520527-29521803	No
MIR1976,R	TSS1500,Bo	7	4,0E-09	3,5E-06	-1,0E-01	chr1:26880207-26881328	Yes
MOV10L1	TSS1500,TS	13	4,4E-09	5,9E-06	1,1E-01	hr22:50528179-5052907	Yes
PRSS42	Body,1stEx	15	5,0E-09	6,7E-05	7,0E-02	chr3:46874897-46876354	No
LOC728024	Body,TSS20	8	5,7E-09	3,1E-04	8,7E-02	chr8:37605359-37606579	No
C15orf26	TSS1500,TS	10	5,8E-09	2,5E-08	1,4E-01	hr15:81426347-8142682	No
TBCD	Body	7	6,5E-09	2,4E-06	8,2E-02	hr17:80872065-8087365	No
LCN6	Body	5	6,5E-09	3,8E-04	-1,5E-01	hr9:139640053-13964140	Yes
ZBED3	3'UTR,Body	6	6,6E-09	2,7E-05	-5,7E-02	chr5:76372556-76373803	No
MGAT5B	TSS1500,Bo	8	6,8E-09	7,3E-07	-7,6E-02	hr17:74868220-7486900	Yes
FIGNL2	1stExon,3'U	7	7,2E-09	9,1E-04	-2,1E-01	hr12:52214119-5221597	No
ZNF880	TSS1500,TS	10	7,7E-09	4,8E-06	5,9E-02	hr19:52872916-5287362	No
		6	1,5E-08	6,5E-07	-1,4E-01	hr2:128165741-12816627	Yes
		8	1,6E-08	3,6E-08	1,2E-01	r12:132663428-1326638	No
CPNE6	TSS1500,1s	5	1,7E-08	5,5E-08	-1,3E-01	hr14:24540174-2454077	Yes
C10orf95	Body,5'UTR	11	1,7E-08	1,2E-04	-6,7E-02	r10:104210483-1042117	Yes
COMT,TXNI	TSS1500,Bo	13	2,2E-08	8,9E-05	-7,5E-02	hr22:19928061-1992955	Yes
		5	2,7E-08	4,3E-04	1,3E-01	hr11:86142104-8614326	No
WIPI2	Body,3'UTR	9	2,9E-08	9,3E-04	1,1E-01	chr7:5270512-5271655	No
		9	3,2E-08	3,9E-04	6,9E-02	r14:106321115-1063224	No
OCT4	TSS1500	15	3,2E-08	3,7E-08	1,6E-01	chr6:31148332-31148748	No
		10	3,8E-08	2,2E-05	1,6E-01	hr2:172972681-17297481	No
DGKA	5'UTR	5	4,1E-08	6,4E-08	8,6E-02	hr12:56329296-5632990	No
NCRNA002	TSS1500,TS	10	4,2E-08	8,8E-08	1,0E-01	chr10:1205222-1205942	No
KLF14	1stExon,TS	22	4,8E-08	9,1E-05	8,3E-02	hr7:130418315-13041993	Yes
S100A2	TSS200,TSS	6	5,5E-08	9,5E-05	-7,7E-02	hr1:153538406-15353940	No
PRTN3	TSS1500,TS	6	5,9E-08	2,3E-07	-8,3E-02	chr19:840737-841082	Yes
MAP3K8	5'UTR	10	6,2E-08	8,4E-04	-7,7E-02	hr10:30723347-3072437	No

CCDC57	Body	7	7,0E-08	2,0E-04	1,1E-01	hr17:80084554-8008595	Yes
		5	7,2E-08	9,6E-06	-9,8E-02	hr17:79924020-7992477	No
		6	9,3E-08	7,6E-05	1,3E-01	r11:113660273-1136613	No
CCDC48	Body	5	9,6E-08	9,8E-06	-1,1E-01	hr3:128722515-12872349	Yes
UNC13D,W	5'UTR,1stE	9	1,0E-07	6,9E-04	-6,8E-02	hr17:73840659-7384201	Yes
FBXL18,MI	Body,TSS20	15	1,1E-07	1,6E-04	-7,4E-02	chr7:5534412-5535934	No
		5	1,2E-07	4,3E-07	8,5E-02	hr16:88453579-8845393	No
TLCD1	Body,5'UTR	5	1,5E-07	2,8E-04	-1,1E-01	hr17:27052676-2705326	No
STK38L	TSS1500,TS	13	1,5E-07	2,3E-04	-1,1E-01	hr12:27396229-2739748	Yes
UCN3	TSS1500,TS	10	1,8E-07	4,2E-06	1,0E-01	chr10:5406423-5407119	No
SORL1	Body	5	1,8E-07	7,1E-04	7,5E-02	r11:121440223-1214408	No
TRPV1	Body	5	1,8E-07	2,4E-06	5,1E-02	chr17:3493176-3493666	No
PAX8,LOC4	Body,TSS15	9	1,9E-07	2,0E-05	1,9E-01	hr2:113992762-11399403	No
SEP9	Body,5'UTR	6	3,2E-07	3,4E-06	6,9E-02	hr17:75473577-7547407	No
		7	3,3E-07	4,0E-05	2,2E-01	chr8:57350090-57351067	No
FUNDC2	TSS1500,TS	12	3,5E-07	1,5E-04	5,5E-02	hrX:154254814-15425553	No
ADORA2A	TSS1500,TS	12	3,7E-07	6,7E-04	8,2E-02	hr22:24822802-2482448	No
GPR97	TSS1500,TS	6	4,1E-07	4,0E-04	-9,9E-02	hr16:57701317-5770223	Yes
STXBP5L	TSS1500,TS	9	4,1E-07	3,4E-04	5,6E-02	hr3:120626653-12062740	Yes
SYCE1	Body,5'UTR	7	4,3E-07	8,3E-06	1,7E-01	r10:135378570-1353797	No
CSF2	TSS1500,TS	5	4,3E-07	4,0E-06	-6,5E-02	hr5:131409159-13140963	No
		6	4,8E-07	5,1E-07	7,4E-02	hr13:40762150-4076246	No
GIPR	TSS1500,TS	6	5,0E-07	7,9E-04	-1,2E-01	hr19:46170937-4617150	No
WNK4	Body	12	5,4E-07	6,8E-04	8,4E-02	hr17:40935998-4093790	No
AXIN2	Body	6	5,4E-07	6,2E-05	9,2E-02	hr17:63534340-6353556	No
PTCRA	TSS1500,TS	5	6,2E-07	1,3E-04	6,1E-02	chr6:42883504-42884350	No
KCNE1	5'UTR,1stE	10	6,6E-07	8,8E-07	1,5E-01	hr21:35831871-3583236	No
RBM46	TSS1500,TS	12	6,9E-07	2,2E-04	1,6E-01	hr4:155702172-15570342	No
CD81	Body	8	9,3E-07	4,9E-04	8,1E-02	chr11:2405835-2407267	No
LTBP1	Body,TSS15	6	9,4E-07	1,1E-06	-1,0E-01	chr2:33359198-33359688	Yes
CACNA2D4	Body	10	1,0E-06	7,3E-04	1,3E-01	chr12:1973208-1974814	No
ANKRD22	1stExon,5'U	6	1,1E-06	6,7E-04	-1,1E-01	hr10:90611604-9061223	Yes
MUC6	Body,1stEx	9	1,1E-06	4,4E-06	9,7E-02	chr11:1036307-1036938	No
GUCY1B2	Body,TSS20	8	1,2E-06	2,0E-05	-8,2E-02	hr13:51639953-5164106	Yes

		5	1,5E-06	6,3E-05	-1,8E-01	chr16:1297276-1297924	Yes
NPY	TSS1500,TS	12	1,6E-06	6,2E-04	5,2E-02	chr7:24323128-24324570	Yes
CUX1	Body	5	1,6E-06	7,8E-06	-1,0E-01	hr7:101596131-10159640	No
		7	1,7E-06	3,8E-04	1,4E-01	hr7:156400033-15640099	No
		8	1,8E-06	2,9E-04	-9,2E-02	r12:125411780-1254131	No
TRIM68	5'UTR,1stE	11	1,8E-06	6,7E-05	1,0E-01	chr11:4628823-4629912	No
RNASE2	TSS1500,1s	6	1,9E-06	9,2E-04	-9,6E-02	hr14:21422605-2142411	Yes
PANX2	Body,3'UTR	6	2,0E-06	5,1E-04	1,8E-01	hr22:50616150-5061782	Yes
L1TD1	TSS1500,TS	8	2,3E-06	5,1E-05	9,5E-02	chr1:62660188-62660861	No
		7	2,3E-06	3,9E-05	1,4E-01	chr7:4347499-4348107	No
PHF20	TSS1500,TS	13	2,4E-06	4,1E-05	5,1E-02	hr20:34359232-3436040	No
LASP1	TSS1500	6	2,6E-06	4,9E-05	-1,5E-01	hr17:37024020-3702476	No
AKAP8L	Body,5'UTR	17	3,4E-06	8,9E-04	-6,4E-02	hr19:15529452-1553087	Yes
PIK3CD	Body	5	3,5E-06	1,6E-05	6,9E-02	chr1:9775302-9776133	No
TMEM196	5'UTR,1stE	16	3,6E-06	1,5E-04	1,1E-01	chr7:19812327-19813525	No
SOCS2	TSS1500,TS	15	3,6E-06	9,2E-04	-5,7E-02	hr12:93962481-9396423	No
LYPD3	1stExon,5'U	8	3,7E-06	6,5E-04	1,4E-01	hr19:43969650-4397068	No
HDAC4	5'UTR	5	4,0E-06	2,7E-04	7,2E-02	hr2:240291068-24029165	No
RP1	5'UTR,Body	8	4,2E-06	8,0E-04	8,4E-02	chr8:55532912-55534103	No
TXNRD1,EI	5'UTR,Body	13	4,6E-06	7,9E-06	1,3E-01	r12:104697193-1046979	No
CRISP2	5'UTR,1stE	9	4,7E-06	6,0E-06	1,4E-01	chr6:49681178-49681774	No
		6	5,1E-06	3,8E-04	1,2E-01	hr6:170337747-17033882	No
RPH3AL	Body	6	5,2E-06	9,1E-06	2,2E-01	chr17:151914-152350	No
		5	5,3E-06	1,4E-05	-1,1E-01	hr22:18267969-1826863	Yes
C5orf42	Body	5	5,6E-06	8,1E-06	9,4E-02	chr5:37208976-37209440	No
P2RY14,ME	1stExon,Bo	5	5,7E-06	1,1E-04	-8,9E-02	hr3:150996078-15099659	No
MAP1LC3B	TSS1500,TS	11	6,4E-06	5,6E-04	-6,4E-02	r12:116996773-1169975	No
DHX40	TSS1500,TS	12	6,4E-06	2,7E-04	1,2E-01	hr17:57642417-5764329	Yes
FKBP5	5'UTR,1stE	7	6,9E-06	1,6E-04	-7,9E-02	chr6:35656242-35657202	Yes
ZNF331	TSS1500,TS	16	7,0E-06	3,1E-04	5,1E-02	hr19:54023732-5402483	No
TMIGD2	TSS200,TSS	5	7,3E-06	2,6E-04	8,2E-02	chr19:4302448-4303012	Yes
DIRAS1	Body,5'UTR	5	7,9E-06	2,0E-05	9,0E-02	chr19:2717293-2717932	No
UBE2DNL	TSS200,Bo	6	8,8E-06	3,1E-04	1,9E-01	chrX:84189001-84189658	No
NUDT16P	TSS1500,TS	10	8,9E-06	7,6E-05	8,6E-02	hr3:131080235-13108122	Yes

C1orf174	Body	6	9,9E-06	4,2E-04	-6,7E-02	chr1:3806715-3808127	Yes
FCER2	5'UTR,1stE	9	1,1E-05	3,1E-05	6,5E-02	chr19:7766717-7767584	Yes
BAI1	Body	5	1,1E-05	1,9E-05	1,5E-01	hr8:143580770-14358148	No
PRR19	5'UTR	5	1,3E-05	5,5E-05	8,0E-02	hr19:42811017-4281148	No
ELOVL2	Body,5'UTR	8	1,3E-05	1,5E-04	1,1E-01	chr6:11044106-11045370	Yes
PLEK	TSS1500,1s	6	1,4E-05	1,6E-04	-9,5E-02	chr2:68591915-68592737	No
ALOX15B	TSS1500,TS	6	1,5E-05	3,2E-05	-7,3E-02	chr17:7942137-7942743	Yes
		7	1,6E-05	3,2E-05	8,2E-02	hr6:164091806-16409309	No
HIST1H3I	1stExon,TS	11	1,7E-05	1,6E-04	-5,0E-02	chr6:27839548-27840257	No
AIP	TSS1500,TS	15	2,0E-05	4,8E-04	9,5E-02	hr11:67250046-6725193	Yes
MTNR1B	TSS1500,TS	11	2,3E-05	7,3E-04	1,1E-01	hr11:92702373-9270353	No
MYT1	TSS1500,TS	11	2,3E-05	5,5E-04	1,3E-01	hr20:62795341-6279641	No
		5	2,3E-05	4,7E-04	1,4E-01	hr17:15652715-1565354	No
GABRD	Body	6	2,3E-05	1,9E-04	6,9E-02	chr1:1959442-1959832	No
CCL1	1stExon,TS	6	2,5E-05	4,8E-05	-8,2E-02	hr17:32690127-3269073	Yes
MS4A3	TSS200,1st	7	2,6E-05	3,0E-05	-9,9E-02	hr11:59823993-5982454	Yes
RPS6KA2	Body	6	2,7E-05	6,3E-05	6,1E-02	hr6:167263025-16726345	No
KIF25	Body	6	2,8E-05	4,7E-05	2,1E-01	hr6:168435636-16843635	No
MGP	Body,1stEx	6	3,0E-05	3,9E-04	-9,5E-02	hr12:15038440-1503956	Yes
ADARB2	Body	5	3,1E-05	3,3E-04	6,0E-02	chr10:1401818-1402293	No
PCDHGB4,P	1stExon,Bo	6	3,2E-05	7,4E-04	1,1E-01	hr5:140768940-14076958	No
ANKRD2	Body	5	3,7E-05	6,8E-05	1,4E-01	hr10:99337800-9933824	No
LPCAT1	Body	5	4,2E-05	6,9E-04	-9,6E-02	chr5:1513791-1514608	No
		6	4,5E-05	1,6E-04	1,6E-01	hr4:190731443-19073219	No
C1S	TSS1500,TS	8	4,6E-05	9,0E-04	-1,0E-01	chr12:7167313-7168545	Yes
CST7	TSS1500,TS	7	5,1E-05	6,1E-05	-9,8E-02	hr20:24929607-2493009	Yes
		6	5,3E-05	1,7E-04	1,4E-01	hr6:160023581-16002414	No
ZNF542	TSS1500,TS	14	5,7E-05	3,1E-04	7,3E-02	hr19:56879207-5688038	No
PDZK1IP1	1stExon,5'U	7	5,9E-05	2,9E-04	-8,0E-02	chr1:47655599-47656423	Yes
USP7	3'UTR	5	6,3E-05	1,6E-04	6,2E-02	chr16:8985593-8986308	No
GPR133	Body	6	6,5E-05	1,0E-04	1,2E-01	r12:131488390-1314889	No
CD300LG	TSS1500,TS	12	6,7E-05	1,9E-04	-6,8E-02	hr17:41923945-4192457	No
MAD1L1	Body	5	7,0E-05	8,3E-04	9,1E-02	chr7:1961785-1962389	No
		8	7,1E-05	5,7E-04	5,7E-02	hr16:85478580-8547930	No

		7	7,3E-05	9,0E-04	1,6E-01	chr7:55516436-55517538	No
TRIOBP	TSS1500,TS	11	7,7E-05	8,6E-05	7,4E-02	:hr22:38092643-3809320	No
TNFRSF13B	Body,1stEx	6	8,2E-05	2,8E-04	5,7E-02	:hr17:16875129-1687595	No
NEURL4	Body	7	8,5E-05	1,2E-04	-7,8E-02	chr17:7227181-7227737	No
PDCL2	Body,5'UTR	7	8,9E-05	1,3E-04	9,7E-02	chr4:56458183-56458622	No
LOC100270	Body,TSS20	11	9,4E-05	1,0E-03	5,3E-02	chr6:26987575-26988289	No
PRDM10	5'UTR,Body	5	1,0E-04	1,4E-04	5,6E-02	r11:129817418-1298177	No
		5	1,1E-04	1,6E-04	8,0E-02	:hr15:27819306-2781992	No
UCN2,COL7	5'UTR,TSS2	5	1,1E-04	9,3E-04	-7,0E-02	chr3:48601117-48602040	No
C15orf2	1stExon,5'U	6	1,2E-04	6,4E-04	1,2E-01	:hr15:24920990-2492178	No
THEG	1stExon,5'U	6	1,2E-04	6,8E-04	1,1E-01	chr19:375746-376152	No
PLA2G4D	Body	7	1,2E-04	1,3E-04	1,1E-01	:hr15:42371511-4237196	No
PHKG1	5'UTR,1stE	10	1,3E-04	1,7E-04	-9,9E-02	chr7:56160409-56161020	Yes
		11	1,3E-04	9,8E-04	9,0E-02	:hr15:95869918-9587099	No
IFT140,TME	Body	7	1,4E-04	5,5E-04	-6,3E-02	chr16:1592816-1593766	No
ADAMTS17	Body	6	1,4E-04	2,5E-04	7,5E-02	r15:100537304-1005381	No
ODZ3	Body,3'UTR	5	1,4E-04	2,5E-04	1,3E-01	hr4:183721183-18372177	No
KRT17	5'UTR,1stE	6	1,5E-04	4,1E-04	-9,4E-02	:hr17:39780836-3978209	Yes
CCDC105	TSS1500,TS	11	1,6E-04	9,3E-04	1,1E-01	:hr19:15120922-1512191	Yes
		10	1,8E-04	6,7E-04	1,7E-01	hr6:166418799-16641994	No
TRPM4	Body	6	1,8E-04	4,6E-04	1,0E-01	:hr19:49699496-4970042	No
ZNF256	Body,5'UTR	12	1,8E-04	3,8E-04	6,0E-02	:hr19:58458572-5845935	No
PCSK6	Body	6	1,9E-04	4,5E-04	-1,8E-01	r15:102009795-1020101	No
		9	1,9E-04	9,2E-04	9,2E-02	chr5:2111458-2112561	No
CDH9	1stExon,5'U	5	2,0E-04	2,0E-04	8,1E-02	chr5:27038605-27038836	No
GARS	Body	6	2,0E-04	4,4E-04	-1,3E-01	chr7:30635214-30636176	Yes
TAF7L	Body,5'UTR	6	2,3E-04	3,3E-04	1,3E-01	hrX:100545995-10054651	No
LTBP3	Body	6	2,3E-04	4,8E-04	7,7E-02	:hr11:65314913-6531562	No
COL23A1	5'UTR,1stE	13	2,4E-04	4,9E-04	-7,6E-02	hr5:178017260-17801818	No
SYCP1	TSS200,1st	8	2,4E-04	6,3E-04	9,2E-02	hr1:115397374-11539812	No
HRNBP3	5'UTR	5	2,7E-04	4,6E-04	7,9E-02	:hr17:77375461-7737584	No
		6	3,0E-04	4,2E-04	1,0E-01	hr6:161100017-16110067	No
NCRNA001	Body,TSS20	6	3,3E-04	5,0E-04	8,5E-02	hr2:132919375-13291986	No
		5	4,8E-04	5,3E-04	1,2E-01	chr7:81240257-81240667	No

GPR132	TSS200,TSS	6	5,4E-04	8,8E-04	-5,1E-02	r14:105531855-10553225	Yes
MTMR9L	TSS1500	5	6,4E-04	7,8E-04	-5,3E-02	chr1:32708234-32708485	No
RECQL5,LO	Body,TSS15	6	7,0E-04	7,6E-04	-6,0E-02	chr17:73629082-73629463	No

Supplementary table 4: Metacore pathway enrichment analysis of gene associated DMRs Supplementary Table S3

A. Metacore Networks	*p-value*	*FDR*	*In Data*
Cell adhesion_Integrin-mediated adhesion	1,46E-03	1,59E-01	12
Cell adhesion_Leucocyte chemotaxis	3,24E-03	1,59E-01	11
Reproduction_Progesterone signaling	4,49E-03	1,59E-01	11
Cell adhesion_Synaptic contact	4,52E-03	1,59E-01	10
Reproduction_Gonadotropin regulation	7,77E-03	2,19E-01	10
Cell adhesion_Cadherins	1,17E-02	2,75E-01	9
Reproduction_FSH-beta signaling pathway	1,71E-02	3,45E-01	8
Apoptosis mediated via PI3K/AKT	2,16E-02	3,81E-01	10
Cytoskeleton_Actin filaments	2,84E-02	4,45E-01	8
Negative regulation of cell proliferation	3,57E-02	4,60E-01	8
Signal transduction_ESR1-membrane pathway	3,91E-02	4,60E-01	5
Immune response_Phagocytosis	3,92E-02	4,60E-01	9
Signal transduction_CREM pathway	5,10E-02	4,72E-01	5
Reproduction_GnRH signaling pathway	5,48E-02	4,72E-01	7
Neurophysiological process_Melatonin signaling	5,79E-02	4,72E-01	3
Androgen receptor signaling cross-talk	6,04E-02	4,72E-01	4
Chemotaxis	6,29E-02	4,72E-01	6
Muscle contraction	6,55E-02	4,72E-01	7
Cell cycle_Meiosis	6,70E-02	4,72E-01	5
Feeding and Neurohormone signaling	6,90E-02	4,72E-01	8
Neurophysiological process_Circadian rhythm	7,09E-02	4,72E-01	4
Cytoskeleton_Spindle microtubules	7,36E-02	4,72E-01	5
Development_Neurogenesis_Synaptogenesis	7,74E-02	4,75E-01	7
Regulation of cytoskeleton rearrangement	8,29E-02	4,76E-01	7
Cytoskeleton_Cytoplasmic microtubules	8,79E-02	4,76E-01	5
TGF-beta, GDF and Activin signaling	9,74E-02	4,76E-01	6
Development_Blood vessel morphogenesis	9,75E-02	4,76E-01	8
Spermatogenesis, motility and copulation	9,94E-02	4,76E-01	8
Neuropeptide signaling pathways	9,97E-02	4,76E-01	6
Development_Neurogenesis_Axonal guidance	1,01E-01	4,76E-01	8
Cell cycle_G1-S Growth factor regulation	1,07E-01	4,86E-01	7
Protein folding_Protein folding nucleus	1,17E-01	5,13E-01	3
Transcription_Chromatin modification	1,23E-01	5,28E-01	5
Phagosome in antigen presentation	1,27E-01	5,29E-01	8
Proliferation_Lymphocyte proliferation	1,39E-01	5,60E-01	7
Cell adhesion_Cell-matrix interactions	1,44E-01	5,62E-01	7
Immune response_TCR signaling	1,49E-01	5,62E-01	6
Immune response_BCR pathway	1,51E-01	5,62E-01	5
Signal transduction_ESR1-nuclear pathway	1,57E-01	5,67E-01	7
Apoptosis mediated by MAPK and JAK/STAT	1,63E-01	5,71E-01	6
Inflammation_IL-2 signaling	1,66E-01	5,71E-01	4
Proliferation_Positive regulation cell proliferation	1,70E-01	5,71E-01	7
Development_Skeletal muscle development	1,75E-01	5,73E-01	5
Apoptosis mediated by external signals via NF-kB	1,95E-01	6,11E-01	4

Signal transduction_ERBB-family signaling	2,00E-01	6,11E-01	3
Apoptosis_Apoptotic mitochondria	2,11E-01	6,11E-01	3
Cell cycle_Core	2,12E-01	6,11E-01	4
Inflammation_IL-4 signaling	2,12E-01	6,11E-01	4
Signal transduction_NOTCH signaling	2,12E-01	6,11E-01	7
Development_Ossification and bone remodeling	2,22E-01	6,23E-01	5
Cell adhesion_Platelet aggregation	2,25E-01	6,23E-01	5
Cell adhesion_Cell junctions	2,41E-01	6,53E-01	5
Translation_Regulation of initiation	2,65E-01	6,98E-01	4
Neurophysiological process_Corticoliberin signali	2,67E-01	6,98E-01	2
Melanocyte development and pigmentation	2,75E-01	7,05E-01	2
Cell adhesion_Attractive and repulsive receptors	2,92E-01	7,35E-01	5
Signal transduction_WNT signaling	3,00E-01	7,36E-01	5
Blood coagulation	3,05E-01	7,36E-01	3
Inflammation_IgE signaling	3,11E-01	7,36E-01	4
Autophagy_Autophagy	3,13E-01	7,36E-01	2
Inflammation_Jak-STAT Pathway	3,36E-01	7,78E-01	5
Apoptosis_Apoptosis stimulation by external sign	3,44E-01	7,81E-01	4
Inflammation_TREM1 signaling	3,49E-01	7,81E-01	4
Development_Neurogenesis in general	3,61E-01	7,96E-01	5
Transport_Potassium transport	3,69E-01	8,01E-01	5
Signal transduction_Leptin signaling	3,79E-01	8,09E-01	3
ERK5 in cell proliferation and neuronal survival	3,93E-01	8,28E-01	1
Protein folding_Response to unfolded proteins	4,17E-01	8,56E-01	2
Cell cycle_G2-M	4,19E-01	8,56E-01	5
Neurophysiological process_Visual perception	4,34E-01	8,58E-01	3
Inflammation_Amphoterin signaling	4,40E-01	8,58E-01	3
Inflammation_IL-6 signaling	4,45E-01	8,58E-01	3
Inflammation_Neutrophil activation	4,55E-01	8,58E-01	5
Death Domain receptors & caspases in apoptosis	4,67E-01	8,58E-01	3
Androgen receptor nuclear signaling	4,82E-01	8,58E-01	3
Platelet-endothelium-leucocyte interactions	4,83E-01	8,58E-01	4
Development_Regulation of angiogenesis	4,87E-01	8,58E-01	5
Transport_Synaptic vesicle exocytosis	4,88E-01	8,58E-01	4
Regulation of epithelial-to-mesenchymal transitio	4,95E-01	8,58E-01	5
Cell cycle_Mitosis	5,05E-01	8,58E-01	4
Neurophysiological process_Long-term potentiati	5,06E-01	8,58E-01	2
Transport_Sodium transport	5,11E-01	8,58E-01	5
Muscle contraction_Relaxin signaling	5,13E-01	8,58E-01	2
Proteolysis_ECM remodeling	5,25E-01	8,58E-01	2
Development_Hemopoiesis, Erythropoietin pathv	5,34E-01	8,58E-01	3
Inflammation_IL-10 anti-inflammatory response	5,38E-01	8,58E-01	2
Signal transduction_Nitric oxide signaling	5,44E-01	8,58E-01	2
Immune response_IL-5 signalling	5,47E-01	8,58E-01	1
Immune response_T helper cell differentiation	5,54E-01	8,58E-01	3
Transport_Calcium transport	5,61E-01	8,58E-01	4
Signal Transduction_BMP and GDF signaling	5,63E-01	8,58E-01	2
Inflammation_IL-13 signaling pathway	5,63E-01	8,58E-01	2
Reproduction_Male sex differentiation	5,75E-01	8,58E-01	5

Nuclear receptors transcriptional regulation	5,82E-01	8,58E-01	4
Immune response_Antigen presentation	5,82E-01	8,58E-01	4
Protein folding_ER and cytoplasm	6,00E-01	8,58E-01	1
Cardiac development VEGF, IP3 and integrin signa	6,01E-01	8,58E-01	3
Immune response_Th17-derived cytokines	6,03E-01	8,58E-01	2
Development_Hedgehog signaling	6,04E-01	8,58E-01	5
Transmission of nerve impulse	6,40E-01	8,58E-01	4

B. Metacore cell signaling pathways	*pValue*	*Min FDR*	*In Data*
NF-AT signaling in cardiac hypertrophy	5,90E-05	2,84E-02	6
Transcription_CREB pathway	1,56E-04	3,75E-02	5
PGE2 pathways in cancer	2,70E-04	4,34E-02	5
Ligand-independent activation of Androgen recep	6,79E-04	7,22E-02	5
Reproduction_GnRH signaling	9,43E-04	7,22E-02	5
Apoptosis and survival_BAD phosphorylation	9,59E-04	7,22E-02	4
Development_A2A receptor signaling	1,05E-03	7,22E-02	4
PDE4 regulation of cyto/chemokine expression in	1,72E-03	9,12E-02	4
hormone action in neurofibromatosis type 1	1,96E-03	9,12E-02	3
G-protein signaling_K-RAS regulation pathway	2,21E-03	9,12E-02	3
Immune response_T cell subsets: secreted signals	2,21E-03	9,12E-02	3
Th17 cells in CF	2,46E-03	9,12E-02	4
Role of HDAC and (CaMK) in control of skeletal my	2,46E-03	9,12E-02	4
Immune response_CD28 signaling	2,81E-03	9,68E-02	4
Development_Regulation of CDK5 in CNS	3,08E-03	9,90E-02	3
Role of VDR in regulation of genes involved in ost	3,84E-03	1,03E-01	4
Cell adhesion_Chemokines and adhesion	4,03E-03	1,03E-01	5
Schwann cells transformation in neurofibromatos	4,07E-03	1,03E-01	4
Immune response_BCR pathway	4,07E-03	1,03E-01	4
Cell cycle_Role of Nek in cell cycle regulation	4,52E-03	1,05E-01	3
Ovarian cancer (main signaling cascades)	4,56E-03	1,05E-01	4
Immune response_CD16 signaling in NK cells	5,96E-03	1,27E-01	4
Immune response_Regulation of T cell function by	6,32E-03	1,27E-01	3
Hedgehog and PTH signaling pathways in bone de	6,32E-03	1,27E-01	3
Growth factors of oligodendrocyte precursor cell	6,82E-03	1,32E-01	3
Immune response_Human NKG2D signaling	7,35E-03	1,36E-01	3
Cell adhesion_Integrin inside-out signaling in T ce	7,63E-03	1,36E-01	4
TCR and CD28 co-stimulation in activation of NF-k	8,48E-03	1,41E-01	3
Role of HP1 family in transcriptional silencing	8,48E-03	1,41E-01	3
growth factor signaling cascades in multiple myel	9,08E-03	1,46E-01	3
Immune response_Murine NKG2D signaling	9,71E-03	1,46E-01	3
Immune response_PIP3 signaling in B lymphocyte	9,71E-03	1,46E-01	3
Neurophysiological process_Melatonin signaling	1,04E-02	1,50E-01	3
Regulatory role of C1q in platelet activation	1,06E-02	1,50E-01	2
Ligand-independent activation of ESR1 and ESR2	1,17E-02	1,58E-01	3
Immune response_ICOS pathway in T-helper cell	1,25E-02	1,58E-01	3
Signal transduction_PTEN pathway	1,25E-02	1,58E-01	3
PTMs in IL-17-induced CIKS-independent signaling	1,25E-02	1,58E-01	3
Development_PIP3 signaling in cardiac myocytes	1,32E-02	1,60E-01	3

Development_Thromboxane A2 signaling pathwa	1,48E-02	1,60E-01	3
Immune response_IL-2 activation and signaling pa	1,48E-02	1,60E-01	3
Signal transduction_IP3 signaling	1,48E-02	1,60E-01	3
Th17 cells in CF (mouse model)	1,48E-02	1,60E-01	3
Development_GM-CSF signaling	1,56E-02	1,60E-01	3
Signal transduction_PKA signaling	1,64E-02	1,60E-01	3
Immune response_Function of MEF2 in T lympho	1,64E-02	1,60E-01	3
Immune response_NFAT in immune response	1,64E-02	1,60E-01	3
Oligodendrocyte differentiation from adult stem	1,64E-02	1,60E-01	3
Degradation of beta-catenin in the absence WNT	1,68E-02	1,60E-01	2
Development_IGF-1 receptor signaling	1,73E-02	1,60E-01	3
Cell adhesion_ECM remodeling	1,73E-02	1,60E-01	3
Additional pathways of NF-kB activation (in the cy	1,82E-02	1,60E-01	3
Translation_Regulation of EIF4F activity	1,82E-02	1,60E-01	3
Immune response_Inhibitory PD-1 signaling in T c	1,82E-02	1,60E-01	3
Immune response_HMGB1/RAGE signaling pathw	1,82E-02	1,60E-01	3
Immune response_HSP60 and HSP70/ TLR signali	1,92E-02	1,62E-01	3
AKT(PKB)/ GSK3 beta cascade in bipolar disorder	1,92E-02	1,62E-01	3
Aberrant B-Raf signaling in melanoma progressio	2,01E-02	1,66E-01	3
Transcription_Epigenetic regulation of gene expre	2,21E-02	1,75E-01	3
ESR1 action on cytoskeleton remodeling and cell	2,22E-02	1,75E-01	2
Cytoskeleton remodeling_Cytoskeleton remodeli	2,26E-02	1,75E-01	4
Development_ERK5 in cell proliferation and neur	2,41E-02	1,79E-01	2
Immune response_Immunological synapse forma	2,42E-02	1,79E-01	3
Role of Parkin in the Ubiquitin-Proteasomal Path	2,62E-02	1,91E-01	2
Immune response_IL-10 signaling pathway	2,75E-02	1,98E-01	3
Leptin signaling via JAK/STAT and MAPK cascades	2,82E-02	2,00E-01	2
Neuroprotective action of lithium	2,87E-02	2,00E-01	3
Immune response_IL-15 signaling	2,98E-02	2,06E-01	3
Apoptosis and survival_NGF signaling pathway	3,04E-02	2,06E-01	2
Immune response_CD40 signaling	3,11E-02	2,07E-01	3
Huntington's disease (general schema)	3,13E-02	2,07E-01	1
HCV-dependent regulation of membrane recepto	3,26E-02	2,12E-01	2
Mucin expression in CF airways	3,62E-02	2,32E-01	3
Neurodisease_Parkin signaling in Parkinson disea	3,72E-02	2,36E-01	2
Colorectal cancer (general schema)	3,96E-02	2,41E-01	2
Transcription factors in segregation of hepatocyti	3,96E-02	2,41E-01	2
Development_Osteopontin signaling in osteoclast	3,96E-02	2,41E-01	2
Cytoskeleton remodeling_Reverse signaling by ep	4,20E-02	2,50E-01	2
Signal transduction_PTMs in IL-23 signaling pathw	4,20E-02	2,50E-01	2
stimulation of fat cell differentiation by Bisphenol	4,45E-02	2,59E-01	2
Chemotaxis_Leukocyte chemotaxis	4,46E-02	2,59E-01	3
Development_EGFR signaling via small GTPases	4,71E-02	2,61E-01	2
Regulation of Apoptosis by Mitochondrial Protein	4,71E-02	2,61E-01	2
Nicotine signaling in glutamatergic neurons	4,71E-02	2,61E-01	2
p53 signaling in Prostate Cancer	4,71E-02	2,61E-01	2
Oxidative stress_Role of ASK1 under oxidative str	4,97E-02	2,66E-01	2
Role of CDK5 in neuronal death and survival	4,97E-02	2,66E-01	2
Apoptosis and survival_Caspase cascade	4,97E-02	2,66E-01	2

Immune response_Th17 cell differentiation	5,23E-02	2,77E-01	2
Immune response_IL-9 signaling pathway	5,50E-02	2,82E-01	2
Cell cycle_The metaphase checkpoint	5,50E-02	2,82E-01	2
Cell adhesion_Tight junctions	5,50E-02	2,82E-01	2
G-protein signaling_H-RAS regulation pathway	5,78E-02	2,93E-01	2
Development_ERBB-family signaling	6,34E-02	3,12E-01	2
Transcription_Receptor-mediated HIF regulation	6,34E-02	3,12E-01	2
Reproduction_Progesterone-mediated oocyte ma	6,63E-02	3,20E-01	2
Role of PKA in cytoskeleton reorganisation	6,63E-02	3,20E-01	2

Supplementary table 5: Relative cell type contributions in healthy and atherosclerosis group.

Cell type	Healthy ± SD (%)	Atherosclerosis ± SD (%)	P-value
Granulocytes	54.3 ± 9.44	63.66 ± 7.69	0,05
CD4+T-cells	17.23 ± 8.6	10.48 ± 6.99	0,11
CD8+T-cells	7.15 ± 4.38	1.66 ± 1.96	0,01
B-cells	2.08 ± 4.05	0.29 ± 0.75	0,26
Monocytes	12.02 ± 3.9	13.35 ± 3.59	0,49
NK-cells	4.28 ± 3.95	9.15 ± 6.8	0,11

Supplementary table 6: CpG probes located in sig-DMRs, associated with atherosclerosis in both blood and plaque tissues

DMR	Probe_ID	Mean_Athero	Mean_Healthy	Delta_Beta	P.Value	adj.P.Val	UCSC_RefG	UCSC_RefG	UCSC_RefG	Relation_to	NearestGe	Distance Nearest Gene
chr17:41277213-41279022	cg02286533	0,769319395	0,628737358	0,140582037	0,0011176	0,2138469	BRCA1;NBR	NM_00730	TSS1500;Bo	Island	BRCA1	0
chr17:41277213-41279022	cg14687474	0,875750819	0,808574884	0,067175935	0,0021927	0,2572073	BRCA1;NBR	NM_00730	TSS1500;Bo	Island	BRCA1	0
chr17:41277213-41279022	cg18372208	0,82479989	0,689299089	0,135500802	0,0031598	0,282286	BRCA1;NBR	NM_00730	TSS1500;Bo	Island	BRCA1	0
chr19:57741325-57743416	cg06643849	0,887085967	0,746513167	0,140572801	0,0003722	0,157509	AURKC;AUR	NM_00101	5UTR;TSS20	Island	AURKC	0
chr19:57741325-57743416	cg23371413	0,859450985	0,6612079	0,198243085	0,0005365	0,174345	AURKC;AUR	NM_00101	5UTR;TSS20	Island	AURKC	0
chr19:50249464-50249927	cg03337057	0,63193732	0,428366365	0,203570956	0,0001598	0,1231756	TSKS	NM_02173	Body	Island	TSKS	0
chr19:50249464-50249927	cg14969099	0,913434958	0,851881374	0,061553584	0,0017112	0,2424612	TSKS	NM_02173	Body	S_Shore	TSKS	0
chr12:213776-215130	cg01239735	0,94091499	0,868166071	0,072748919	1,50E-05	0,0634674	IQSEC3;IQS	NM_00117	Body;5UTR	Island	IQSEC3	0
chr12:213776-215130	cg19186614	0,93635907	0,886723119	0,049635951	5,75E-05	0,0920725	IQSEC3;IQS	NM_00117	Body;5UTR	Island	IQSEC3	0
chr1:117317838-117318232	cg24635754	0,901140315	0,82212893	0,079011385	0,001115	0,2137478					CD2	6333
chr15:81426347-81426820	cg14380111	0,162924309	0,054557839	0,108366469	0,0048817	0,3116616	C15orf26	NM_17352	TSS1500	N_Shore	C15orf26	0
chr15:81426347-81426820	cg18028999	0,217744873	0,079524316	0,138220557	0,0160798	0,4137776	C15orf26	NM_17352	TSS200	Island	C15orf26	0
chr15:81426347-81426820	cg18837035	0,126368711	0,048629734	0,077738977	0,0059716	0,3264231	C15orf26	NM_17352	TSS200	Island	C15orf26	0
chr15:81426347-81426820	cg25029264	0,125813054	0,037387696	0,088425358	0,0010878	0,2125824	C15orf26	NM_17352	TSS1500	N_Shore	C15orf26	0
chr15:81426347-81426820	cg26546864	0,143399842	0,064135833	0,079264009	0,0222668	0,4462743	C15orf26	NM_17352	TSS1500	N_Shore	C15orf26	0
chr15:81426347-81426820	cg27045999	0,195521202	0,080937266	0,114583936	0,0099687	0,3705789	C15orf26	NM_17352	TSS200	Island	C15orf26	0
chr15:81426347-81426820	cg27136994	0,165242794	0,063554691	0,101688103	0,0161723	0,4143803	C15orf26	NM_17352	TSS200	Island	C15orf26	0
chr11:86142104-86143260	cg09610772	0,901907031	0,808110499	0,093796531	0,0018132	0,2447598				Island	CCDC81	8255
chr11:86142104-86143260	cg15146462	0,84747013	0,712676717	0,134793413	0,0010976	0,2132524				Island	CCDC81	8435
chr8:57350090-57351067	cg07776626	0,944281917	0,871672556	0,072609361	0,004161	0,301483				N_Shore	PENK	2736
chr8:57350090-57351067	cg17761419	0,836812124	0,715576704	0,12123542	0,0121592	0,3880465				N_Shore	PENK	2762
chr21:35831871-35832364	cg00421848	0,502384639	0,357816504	0,144568135	0,000503	0,1715159	KCNE1;KCN	NM_00021	5UTR;TSS15	Island	KCNE1	0
chr11:1036307-1036938	cg00338749	0,834469069	0,757829919	0,07663915	0,0252392	0,4593692	MUC6;MUC	NM_00596	1stExon;5U	Island	MUC6	0
chr11:1036307-1036938	cg22685816	0,854610204	0,761174946	0,093435258	0,0019889	0,2501396	MUC6;MUC	NM_00596	1stExon;5U	Island	MUC6	0
chr11:1036307-1036938	cg24404533	0,923500741	0,879889364	0,043611378	0,0085879	0,3586327	MUC6	NM_00596	TSS200	Island	MUC6	59
chr7:156400033-156400990	cg13501951	0,496825541	0,37219494	0,124630601	0,0002384	0,1392075				Island	C7orf13	30748
chr7:156400033-156400990	cg18584424	0,296409984	0,185722555	0,110687428	0,039776	0,5059543				S_Shore	C7orf13	30279
chr7:4347499-4348107	cg11746156	0,904618412	0,809487485	0,095130926	0,0006496	0,1854644				N_Shore	SDK1	38878
chr8:55532912-55534103	cg10993470	0,969539334	0,934962076	0,034577258	0,0033847	0,2874134	RP1	NM_00626	Body	Island	RP1	0
chr8:55532912-55534103	cg18794475	0,921655706	0,861380453	0,060275253	0,0032318	0,2839265	RP1	NM_00626	Body	Island	RP1	0
chr12:104697193-104697983	cg05057777	0,239444148	0,144074819	0,09536933	0,0451489	0,5198753	EID3;TXNRD	NM_00100	1stExon;5U	Island	TXNRD1	0
chr12:104697193-104697983	cg09477407	0,253938478	0,136001183	0,117937295	0,0308046	0,4783498	TXNRD1;TX	NM_18272	5UTR;5UTR	Island	TXNRD1	0
chr12:104697193-104697983	cg27205904	0,343249116	0,229823996	0,113425121	0,0440633	0,5170938	TXNRD1;TX	NM_18272	5UTR;5UTR	Island	TXNRD1	0
chr6:49681178-49681774	cg01076129	0,763634389	0,650239797	0,113394592	0,0189511	0,428839	CRISP2;CRIS	NM_00114	TSS200;TSS200;TSS200		CRISP2	30
chr6:49681178-49681774	cg04595372	0,742724409	0,615226118	0,127498291	0,0165931	0,4159746	CRISP2;CRIS	NM_00114	5UTR;5UTR;1stExon;1st		CRISP2	0
chr6:49681178-49681774	cg08942800	0,855524714	0,740833292	0,114691422	0,019426	0,4312264	CRISP2;CRIS	NM_00114	TSS200;TSS200;TSS200		CRISP2	6
chr6:49681178-49681774	cg14997592	0,750574846	0,619681437	0,130893409	0,0316878	0,4816314	CRISP2;CRIS	NM_00114	5UTR;5UTR;5UTR;5UTR		CRISP2	0
chr6:49681178-49681774	cg25390787	0,751300982	0,628782992	0,12251799	0,0375233	0,4996034	CRISP2;CRIS	NM_00114	TSS200;TSS200;TSS200		CRISP2	0

chr6:49681178-49681774	cg26715042	0,718478604	0,576672823	0,141805781	0,018922	0,4287067	CRISP2;CRIS	NM_00114	TSS200;TSS200;TSS200		CRISP2	3
chr6:170337747-170338826	cg02548777	0,673131671	0,58091394	0,092217731	0,0211205	0,4407352				Island	LINC00574	135856
chr5:37208976-37209440	cg14824766	0,885725978	0,791455388	0,09427059	0,0040174	0,2984729	C5orf42	NM_02307	Body	N_Shore	C5orf42	0
chr8:143580770-143581481	cg09544050	0,737231449	0,591718752	0,145512697	0,0045055	0,3064358	BAI1	NM_00170	Body	Island	ADGRB1	0
chr19:42811017-42811489	cg01127878	0,948844589	0,903626406	0,045218183	0,0104743	0,3744954	PRR19	NM_19928	5UTR	Island	PRR19	0
chr19:42811017-42811489	cg08835342	0,9177182	0,86160739	0,05611081	0,0034667	0,2888046	PRR19	NM_19928	5UTR	Island	PRR19	0
chr19:42811017-42811489	cg14242928	0,926010527	0,84593213	0,080078397	0,0009715	0,2056228	PRR19	NM_19928	5UTR	Island	PRR19	0
chr11:92702373-92703536	cg15559898	0,238021294	0,128511248	0,109510046	0,0056749	0,3233587	MTNR1B	NM_00595	Body	Island	MTNR1B	0
chr17:15652715-15653548	cg01443020	0,366984076	0,228574459	0,138409617	2,67E-05	0,0691405				Island	TBC1D26	3616
chr6:168435636-168436353	cg08476511	0,712762724	0,56299664	0,149766083	0,0044707	0,305591	KIF25;KIF25	NM_00535	Body;Body	Island	KIF25	0
chr6:168435636-168436353	cg12003941	0,407590129	0,306693115	0,100897015	0,0123491	0,3891141	KIF25;KIF25	NM_00535	Body;Body	Island	KIF25	0
chr6:168435636-168436353	cg18319852	0,480242441	0,366737211	0,11350523	0,0189496	0,428839	KIF25;KIF25	NM_00535	Body;Body	S_Shore	KIF25	0
chr6:168435636-168436353	cg24246628	0,673486161	0,468385431	0,20510073	0,0019374	0,2484644	KIF25;KIF25	NM_00535	Body;Body	Island	KIF25	0
chr6:160023581-160024144	cg10672567	0,777401081	0,645736662	0,131664419	0,0168686	0,417322					SOD2	76220
chr6:160023581-160024144	cg15166039	0,624456588	0,514354231	0,110102357	0,0393062	0,5049873					SOD2	76521
chr6:160023581-160024144	cg26873880	0,801776902	0,658862265	0,142914637	0,011639	0,3847668					SOD2	76458
chr17:7227181-7227737	cg24517323	0,488103397	0,560654078	-0,07255068	0,0208711	0,4392202	NEURL4;NE	NM_03244	Body;Body	Island	NEURL4	0
chr4:56458183-56458622	cg20125971	0,930030522	0,845873425	0,084157097	0,0013585	0,2273349	PDCL2;PDC	NM_15240	5UTR;1stExon		PDCL2	0
chr15:27819306-27819924	cg09088406	0,879806822	0,815404267	0,064402555	0,0276491	0,4681953					GABRG3	40932
chr19:375746-376152	cg27227797	0,795194293	0,686776134	0,10841816	0,0008273	0,1980924	THEG;THEG	NM_01658	1stExon;1st	Island	THEG	0
chr15:42371511-42371967	cg04202736	0,962768096	0,905271781	0,057496316	0,0020544	0,2524519	PLA2G4D	NM_17803	Body	Island	PLA2G4D	0
chr15:42371511-42371967	cg17867243	0,837752422	0,726472697	0,111279725	0,0094388	0,3653636	PLA2G4D	NM_17803	Body	Island	PLA2G4D	0
chr4:183721183-183721778	cg05673204	0,958753202	0,869694529	0,089058672	0,0016584	0,2400078	ODZ3	NM_00108	3UTR	Island	TENM3	0
chr6:166418799-166419943	cg19251564	0,691325267	0,523816464	0,167508803	0,0016719	0,2407422				N_Shelf	LINC00602	15818
chr19:49699496-49700427	cg16749456	0,884473478	0,781162914	0,103310564	0,007162	0,3417963	TRPM4	NM_01763	Body	Island	TRPM4	0
chr5:2111458-2112561	cg03540794	0,937183976	0,90437399	0,032809986	0,0464552	0,522173				Island	IRX4	224815
chr5:2111458-2112561	cg23319696	0,892272598	0,800659345	0,091613253	0,0031169	0,2809945				Island	IRX4	224576
chr5:27038605-27038836	cg07350977	0,84085902	0,76016355	0,08069547	0,010289	0,3732852	CDH9	NM_01627	TSS200		CDH9	146
chr5:27038605-27038836	cg11155432	0,825938765	0,757612369	0,068326395	0,0396767	0,5056214	CDH9	NM_01627	TSS200		CDH9	112
chr5:27038605-27038836	cg12864235	0,898679172	0,834589074	0,064090098	0,0280909	0,4692157	CDH9	NM_01627	TSS200		CDH9	92
chr2:132919375-132919865	cg22923398	0,662532406	0,601058552	0,061473854	0,0038762	0,2957642	NCRNA001	NR_027019	TSS1500;Body		ANKRD30B	0

Supplementary table 7: Ingenuity pathway enrichment analysis of gene associated DMRs of Supplementary Table S6

Ingenuity Pathway analysis	-log(p-value)	Molecules
Oxidative Stress	2,68	SOD2,BRCA1
Antioxidant Action of Vitamin C	2,25	PLA2G4D,TXNRD1
Thioredoxin Pathway	2,18	TXNRD1,
p53 Signaling	2,05	ADGRB1,BRCA1
Superoxide Radicals Degradation	1,98	SOD2,
Vitamin-C Transport	1,77	TXNRD1,
DNA Double-Strand Break Repair by Homologous Recombination	1,74	BRCA1,
NRF2-mediated Oxidative Stress Response	1,64	SOD2,TXNRD1
DNA damage-induced 14-3-3σ Signaling	1,63	BRCA1,
GADD45 Signaling	1,61	BRCA1,
Positive Acute Phase Response Proteins	1,56	SOD2
MIF-mediated Glucocorticoid Regulation	1,46	PLA2G4D,
MIF Regulation of Innate Immunity	1,35	PLA2G4D,
